# Comparison of different surgical techniques for chronic subdural hematoma: a network meta-analysis

**DOI:** 10.3389/fneur.2023.1183428

**Published:** 2023-07-26

**Authors:** Youjia Qiu, Minjia Xie, Aojie Duan, Ziqian Yin, Menghan Wang, Xi Chen, Zhouqing Chen, Wei Gao, Zhong Wang

**Affiliations:** ^1^Department of Neurosurgery, The First Affiliated Hospital of Soochow University, Suzhou, Jiangsu, China; ^2^Suzhou Medical College of Soochow University, Suzhou, Jiangsu, China; ^3^Department of Epidemiology and Statistics, School of Public Health, Medical College, Zhejiang University, Hangzhou, China; ^4^School of Health, Brooks College (Sunnyvale), Milpitas, CA, United States

**Keywords:** chronic subdural hematoma, cSDH, burr hole craniotomy, twist drill craniotomy, mini-craniotomy

## Abstract

**Background:**

Chronic subdural hematoma (CSDH) is a neurosurgical condition with high prevalence. Many surgical approaches are recommended for treating CSDH, but there needs to be a consensus on the optimal technique. This network meta-analysis (NMA) compared the efficacy and safety of different surgical treatments for CSDH.

**Methods:**

Electronic databases, including PubMed, Embase, and the Cochrane Library, were searched for relevant studies up to February 2023. An NMA was performed to compare the outcomes of patients with CSDH treated by single-hole or double-hole craniotomy (SBHC and DBHC, respectively), twist-drill craniotomy (TDC), mini-craniotomy, and craniotomy. The NMA protocol was registered at INPLASY (registration no. 202320114).

**Results:**

The NMA included 38 studies with 7,337 patients. For efficacy outcomes, DBHC showed the highest surface under the cumulative ranking area (SUCRA) values for recurrence (96.3%) and reoperation (87.4%) rates. DBHC differed significantly from mini-craniotomy in recurrence rate (odds ratio [OR] = 0.58, 95% confidence interval [CI]: 0.35, 0.97) and from SBHC (OR = 0.48, 95% CI: 0.25, 0.91) and TDC (OR = 0.40, 95% CI: 0.20, 0.82) in reoperation rate. For operative time, TDC was superior to SBHC (mean difference [MD] = −2.32, 95% CI: −3.78 to −0.86), DBHC (MD = −3.61, 95% CI: −5.55, −1.67), and mini-craniotomy (MD = −3.39, 95% CI: −5.70, −1.08). Patients treated by TDC had a shorter hospital stay than those treated by SBHC (MD = −0.82, 95% CI: −1.51, −0.12). For safety outcomes, there were no significant differences between groups in mortality and complication rates; however, mini-craniotomy (79.8%) and TDC (78.1%) had the highest SUCRAs.

**Conclusion:**

DBHC may be the most effective surgical treatment for CSDH based on the low recurrence and reoperation rates, although all examined techniques were relatively safe.

**Systematic review registration:**

https://inplasy.com/inplasy-2023-2-0114/

## 1. Introduction

Chronic subdural hematoma (CSDH) is a common neurosurgical condition caused by the tearing of bridging veins that traverse the dural border cell layer (1). The global annual incidence of CSDH is 1.72 to 20.6 per 100,000 people and is higher among the elderly (2, 3). The median age and incidence of CSDH have been increasing in some countries (4). Approximately 60%–80% of patients have experienced a traumatic injury before CSDH (5).

Many factors contribute to the development of CSDH including the high permeability of new blood vessels, the release of inflammatory mediators, and local coagulation (6). Additionally, activation of the fibrinolytic system and formation of new capillaries promoted by angiogenic factors may lead to the continuous expansion of the hematoma area and increased intracranial pressure due to a space-occupying effect (1, 7). CSDH is usually treated by pharmacotherapy or surgery (8, 9). The former is used in asymptomatic cases or when the CSDH is small and does not cause brain compression. In contrast, the latter is preferred in patients exhibiting significant neurologic symptoms related to the hematoma (10–12).

The main surgical approaches for CSDH are craniotomy and drilling surgeries such as burr hole craniotomy (BHC), twist drill craniotomy (TDC), and craniotomy/mini-craniotomy (13). Although BHC is the most frequently used by neurosurgeons, there is no consensus on the optimal technique (2, 14). A retrospective study found that TDC was convenient and could be rapidly performed under local anesthesia, but craniotomy was more effective in patients with extensive loculated membrane formation (15, 16), although the large bone window poses a risk for elderly patients with comorbidities (17). There is a paucity of level I evidence for the optimal surgical approach for CSDH treatment, which is needed for clinical decision-making. This was addressed in the present study by performing a network meta-analysis (NMA) to compare efficacy and safety outcomes in patients with CSDH treated with different surgical techniques.

## 2. Materials and methods

The NMA adhered to the Preferred Reporting Items for Systematic Review and Meta-Analyses (PRISMA) checklist (18) and was registered at INPLASY (registration no. 202320114).

### 2.1. Inclusion and exclusion criteria

Inclusion criteria were as follows: (1) the study was a randomized controlled trial (RCT), prospective study, or retrospective study; (2) the article was in English; (3) participants were adult patients (>18 years) diagnosed with CSDH by computed tomography scan or magnetic resonance imaging; (4) patients received surgical treatments including single BHC (SBHC), double BHC (DBHC), TDC, craniotomy (>30 mm), and mini-craniotomy (≤30 mm), with the surgical procedure described in the article; and (5) the study reported recurrence rate, reoperation, favorable outcome, duration of hospitalization, and operative time as efficacy outcomes and rate of complications and mortality as safety outcomes (although not all were required in any given study). Exclusion criteria were as follows: (1) participants were < 18 years of age or had neurodegenerative, medical, or psychiatric disorders or other intracranial diseases (i.e., intracranial space-occupying lesion); (2) the article was a conference abstract, commentary, review, or protocol; and (3) the full text or dataset was unavailable.

### 2.2. Search strategy

Two investigators (YJQ and MJX) independently searched PubMed, Embase, and the Cochrane Library from inception to 1 February 2023, for relevant literature on surgical treatments for CSDH using the following search terms: “Hematoma, Subdural, Chronic,” AND “Craniotomy” OR “Drainage.” The full details of search strategies applied to the different databases are shown in Supplementary Table S1. The investigators also screened systematic reviews and meta-analyses to ensure that no relevant study was omitted.

### 2.3. Data extraction and risk-of-bias assessment

Two researchers (YJQ And MJX) independently screened article titles and abstracts, followed by the full text using the bibliographic software EndNote X9 (Thomson Reuters, United States) for preparing systematic reviews. We rigorously reviewed and evaluated the articles that met the inclusion criteria during the full-text screening phase while referring to the exclusion criteria outlined earlier. The list of excluded articles during the full-text screening process is provided in Supplementary Table S3. Disagreements were resolved through discussion with a third researcher (ZQY). The 2 researchers (YJQ and MJX) independently extracted the following data from each article: baseline characteristics; publication year; author; study design; sample size; age; sex; follow-up interval; and clinical features including unilateral or bilateral hematoma, hematoma thickness and volume, and midline shift. The risk of bias in RCTs and cohort studies was assessed with Cochrane’s Risk of Bias (19) and Risk of Bias in Non-randomized Studies of Interventions (20), respectively, with each domain classified as having “low,” “unclear,” or “high” risk of bias. Any disagreements were resolved through discussion with the third reviewer (ZQY).

### 2.4. Quality of evidence

The quality of included studies was evaluated according to the Grading of Recommendations, Assessment, Development, and Evaluation (GRADE) scale—which has 4 levels of evidence—by 2 investigators (AJD and MHW) who did not participate in data extraction. Detailed information on the classification of different domains by the GRADE Working Group can be found in Supplementary Table S4. The included studies were assessed on the following 5 domains: risk of bias, inconsistency, indirectness, imprecision, and publication bias.

### 2.5. Statistical analysis

Before conducting the NMA, the clinical methodology of the included studies was assessed to determine the appropriateness of the transitivity assumption. We also performed a pairwise meta-analysis with a random-effects model using RevMan (version 5.4). Dichotomous and continuous outcomes are presented as odds ratio (OR) and standard difference (SD) with 95% confidence interval (CI). I^2^ values were used to evaluate statistical heterogeneity, with values > 75% considered to reflect high heterogeneity (21).

We performed the NMA with a random-effects model to compare all surgical techniques based on direct and indirect evidence using STATA v17.0, which generated a network graph in which each node represented an intervention and the size of the node represented the number of subjects. The thickness of lines between the nodes indicated the sample size of included studies. The Chi-squared Q test and I^2^ statistic were used to evaluate heterogeneity between trials in the NMA. Consistency between direct and indirect evidence was assessed using the node splitting approach, and *p*-values < 0.05 were considered as indicating inconsistency (22). To evaluate the convergence of the model, we utilized the tract and density plot, as well as the Brooks-Gelman-Rubin diagnosis plot. When the overlap region of each MCMC chain in the tract encompasses the majority of the chain’s fluctuation range, the density plot exhibits a normal distribution, and the bandwidth remains stable and approaches zero, we determined that the model had achieved a high degree of convergence.

To rank the performance of different surgical drainage treatments in terms of efficacy and safety outcomes, we calculated the surface under the cumulative ranking area (SUCRA), which represents the relative probability of an intervention being superior to the others (23). The SUCRA of each intervention ranged from 0 to 1, with a higher value (close to 1) indicating the optimal choice. A 2-tailed test was used for all analyses, and *p* < 0.05 was considered as statistically significant. Publication bias was evaluated with a funnel plot, with a symmetric distribution indicating no publication bias.

## 3. Results

### 3.1. Results of search process

**Figure 1:**
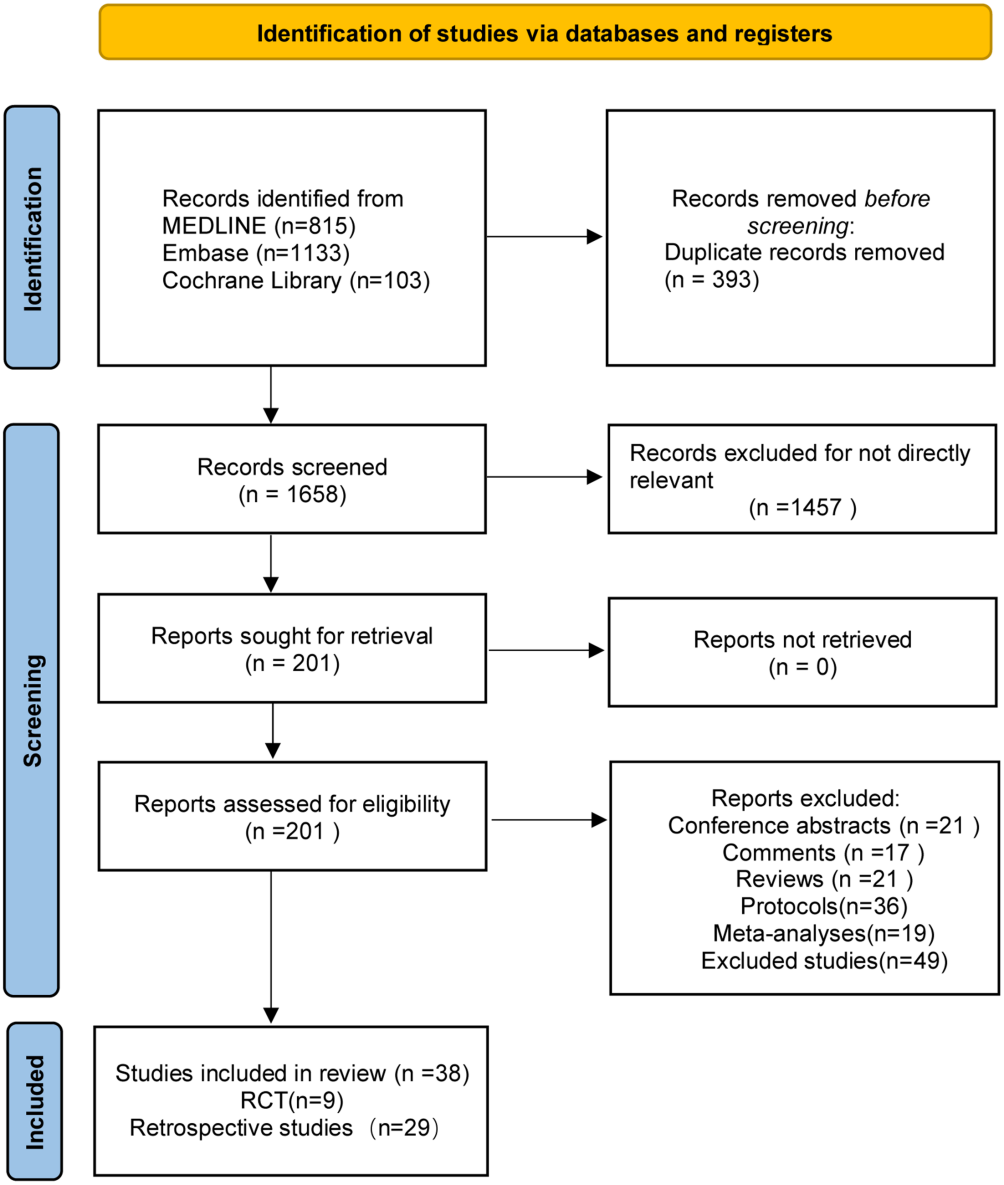
The study search, selection, and inclusion process.

The original search yielded 2,051 studies; 393 of these were removed because of duplication. A total of 1,658 titles and abstracts were screened, and 1,457 studies were excluded because of the irrelevance of the subject matter. Of 201 full articles, 163 were excluded (21 conference abstracts, 17 commentaries, 21 reviews, 36 protocols, 19 meta-analyses, and 49 studies that did not meet the inclusion criteria). Finally, 38 articles (9 RCTs and 29 retrospective studies) that used the 5 surgical treatments of interest were included in the NMA (Figure 1).

### 3.2. Characteristics of included studies

There was a total of 7,337 patients with CSDH included in the analysis. The surgical techniques were TDC (*n* = 15 studies), SBHC (*n* = 30), DBHC (*n* = 21), mini-craniotomy (*n* = 13), and craniotomy (*n* = 1). The detailed clinical characteristics, interventions, and follow-up durations are shown in Table 1.

**Table 1:** Baseline characteristics of the included trials for patients.

Study	Year	Countries	Centers	Study period (months)	Outcome events	Treatment group (no. of participants)	Male (%)	Mean age ±SD (years)
Doria et al.	2020	Italy	1	30	a, f, g, d, e	SBHC	54.4	66.5 ± 6.0
						DBHC	55.1	67.2 ± 7.1
Nayıl et al.	2014	India	1	24	a	SBHC	56.9	61.0
						DBHC	54.8	61.7
Rafi et al.	2017	Iran	1	12	b, f, g, d	SBHC	NA	NA
						DBHC	NA	NA
Goyal et al.	2018	India	1	6	a, b, c, f, g	DBHC	85.0	62.9
						TDC	85.0	60.5
Gokmen et al.	2008	Turkey	1	6	a, b, c, g	SBHC	NA	NA
						TDC	NA	NA
Xu et al.	2018	China	1	3	a, b, c, d, f, g	SBHC	85.0	66.0 ± 16.7
						TDC	80.0	66.2 ± 10.1
Duerinck et al.	2022	Belgium	4	6	a, b, c, e, f, g	DBHC	60.8	74.3 ± 13.0
						MC	70.2	73.2 ± 12.5
						TDC	62.2	74.3 ± 14.8
Sale et al.	2020	Nigeria	1	6	a, e	SBHC	81.8	52.8
						DBHC	88.1	53.1
Jang et al.	2015	Korea	1	3	a, b, c, g	SBHC	58.1	70.0
						DBHC	71.9	72.5
Fernandez et al.	2022	Spain	1	NA	a, c, f, g	SBHC	66.0	79.0
						DBHC	69.0	80.0
Heringer et al.	2017	Brasil	1	21	a, f	SBHC	NA	NA
						DBHC	NA	NA
Lee et al.	2004	Germany	1	12	b	DBHC	65.8	70.0
						MC	61.9	68.0
						CRAN	38.5	73.0
Lee et al.	2009	Korea	1	6	a, b, d, e, f	SBHC	64.0	65.2 ± 14.3
						DBHC	78.1	65.3 ± 12.1
						MC	80.0	63.7 ± 13.0
White et al.	2010	United Kingdom	1	3	a, b, c, f, g	SBHC	72.0	63.0
						MC	66.0	73.0
Hussain et al.	2017	United Kingdom	1	50	g	DBHC	NA	NA
						MC	NA	NA
Stavrinou et al.	2017	Germany	1	NA	a	BHC	NA	NA
						DBHC	NA	NA
						MC	NA	NA
Raghavan et al.	2019	United States	1	12	b, d,	SBHC	60.8	72.2 ± 13.0
						CRAN	69.2	72.7 ± 13.5
Haron et al.	2019	Australia	1	12	b, g	SBHC	NA	NA
						MC	NA	NA
Shim et al.	2019	Korea	1	6	a, d,	SBHC	73.3	74.5
						MC	86.7	73.2
Gazzeri et al.	2020	Italy	1	1	a, b, f, g	SBHC	63.9	74.9
						MC	67.6	76.4
Vemula et al.	2020	India	1	NA	a, c, g	DBHC	NA	NA
						MC	NA	NA
Zolfaghari et al.	2021	Sweden	2	12	a, b, f, g	SBHC	65.9	74.1 ± 12.9
						MC	71.1	75.6 ± 11.6
Singh et al.	2011	India	1	3	a, c, f, g	DBHC	96.1	61.2
						TDC	10.4	59.8
Gernsback et al.	2016	United States	2	NA	a	SBHC	72.6	69.0 ± 11.0
						DBHC	71.1	64.0 ± 16.0
Kansal et al.	2010	India	1	6	a	SBHC	NA	NA
						DBHC	NA	NA
Han et al.	2009	Korea	1	1	a	SBHC	28.3	62.7 ± 13.7
						DBHC	71.7	62.2 ± 15.7
Taussky et al.	2008	Switzerland	1	2	a, b, f, g	SBHC	72.0	70.0 + 12.6
						DBHC	78.0	69.0 + 11.6
Thavara et al.	2019	India	1	3	a, b, c, d, e, f, g	SBHC	76.1	61.3±13.2
						TDC	69.5	73.3±10.8
Kim et al.	2014	Korea	1	3	a, c, d, e	SBHC	47.3	70.6
						TDC	58.3	67.9
Smely et al.	1997	Germany	1	3	a, b, f, g	SBHC	63.6	70.0±15.0
						TDC	63.6	69.7±12.6
Williams et al.	2000	United States	1	6	a, b, g	SBHC	75.0	57.4
						TDC	75.0	63.0
Lin et al.	2011	China	2	6	a, b, c, f, g	SBHC	86.5	62.3±24.5
						TDC	80.7	63.1±21.1
Certo et al.	2019	Italy	1	30	a, b, f	SBHC	60.0	77.1
						TDC	63.3	75.7
Garber et al.	2016	United States	2	NA	a, b, d	DBHC	58.6	69.4
						TDC	73.3	76.7
Lee et al.	2016	Korea	1	3	a, b, d	SBHC	75.0	63.5
						TDC	48.8	67.9
Wang et al.	2017	China	1	3	a, b, c, d, e, g	SBHC	84.4	67.3 ± 12.9
						TDC	73.7	68.2 ± 18.5
Wang et al.	2016	China	1	4	a, b, c, d, f, g	SBHC	83.0	66.6 ± 13.1
						TDC	83.8	69.4 ± 12.6
Katsigiannis et al.	2017	Germany	1	3	c	SBHC	NA	NA
						DBHC	NA	NA
						MC	NA	NA

SBHC, single burr hole craniotomy; DBHC, double burr hole craniotomy; TDC, twist drill craniotomy; MC, mini-craniotomy; CRAN, craniotomy. a, recurrence; b, reoperation; c, favorable outcomes; d, hospital stay; e, operation time; f, complication; g, mortality.

### 3.3. Pairwise meta-analysis of efficacy and safety outcomes

In the pairwise meta-analysis, DBHC was significantly more effective than TDC (OR = 0.38, 95% CI: 0.18, 0.80, *p* = 0.01), as reflected by the recurrence rate. SBHC was inferior to DBHC in terms of reoperation rate (OR = 2.79, 95% CI: 1.03, 7.58, *p* = 0.04) and craniotomy (OR = 2.28, 95% CI: 1.03, 4.85, *p* = 0.03), but was associated with fewer reoperations than TDC (OR = 0.40, 95% CI: 0.18, 0.90, *p* = 0.03). More favorable outcomes were achieved with SBHC than with mini-craniotomy (OR = 1.96, 95% CI: 1.10, 3.48, *p* = 0.02). Patients treated with SBHC had a longer length of hospital stay than those treated with TDC (mean difference [MD] = 3.08, 95% CI: 1.76, 4.39, *p* < 0.0001); however, operative time was shorter with TDC than with SBHC (MD = –28.46, 95% CI: −19.01, −37.92, *p* < 0.0001), DBHC (MD = −33.00, 95% CI: −27.07, −38.93, *p* < 0.0001), and mini–craniotomy (MD = –56.32, 95% CI:–66.47, −46.18, *p* < 0.0001). Operative time was shorter with SBHC than with DBHC (MD = −27.75, 95% CI: −30.30, −25.19, *p* < 0.0001) and mini-craniotomy (MD = −39.41, 95% CI: −54.01, −24.81, *p* < 0.0001), and shorter with DBHC than with mini-craniotomy (MD = −13.4, 95% CI: −21.41, −5.38, *p* = 0.001).

For safety outcomes, the mortality rate was significantly higher with DBHC than with mini-craniotomy (OR = 2.33, 95% CI: 1.10, 4.96, *p* = 0.02). However, there were no significant differences in complication and mortality rates among other surgical treatments (Supplementary Table S2).

### 3.4. NMA of all outcomes

**Figure 2:**
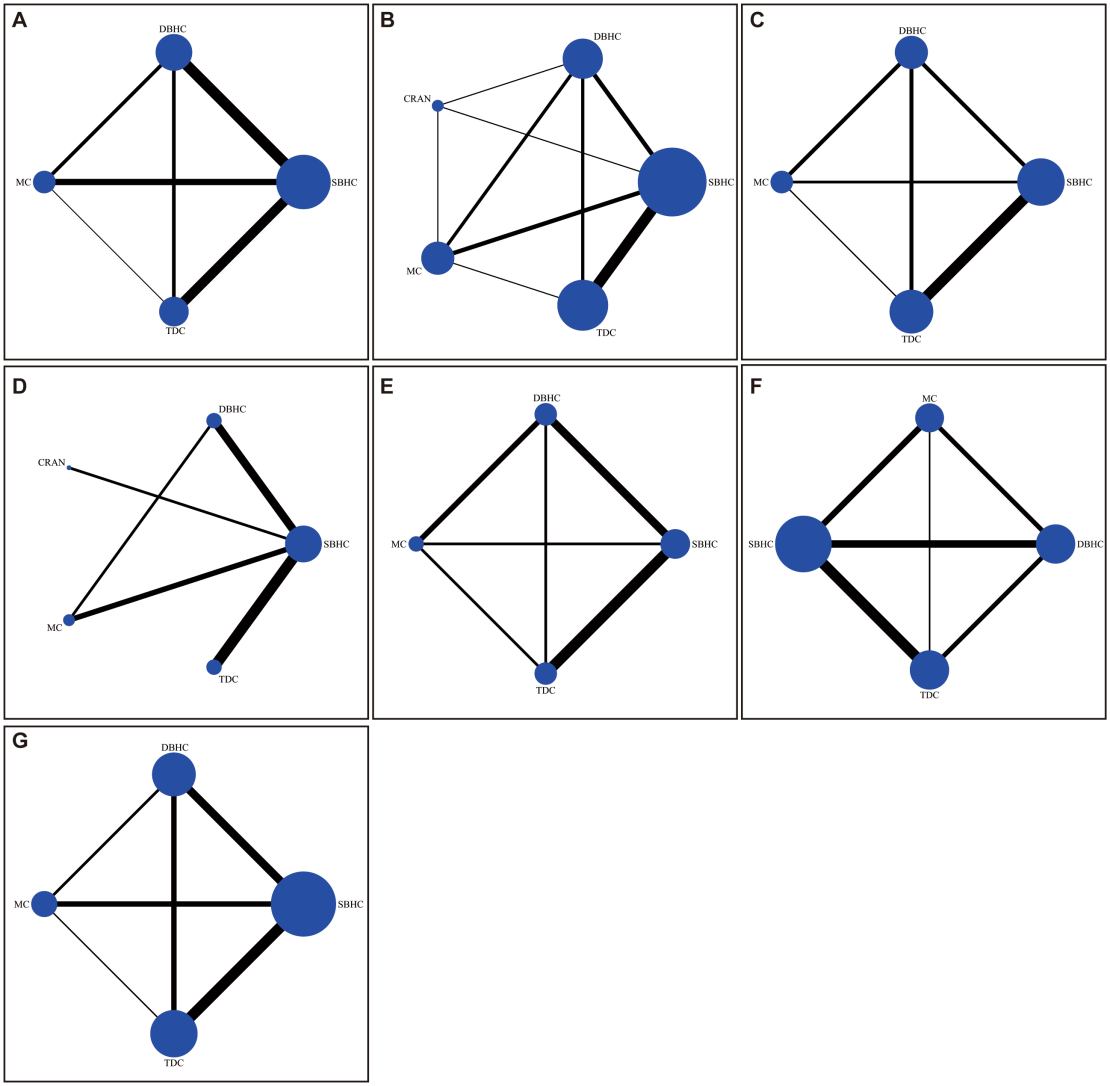
Network of trials comparing different surgical treatments for CSDH. The size of circles represented the number of participants for each intervention, and the width of lines represented the number of trials compared between treatments. **(A)** Recurrence. **(B)** Reoperation. **(C)** Favorable outcome. **(D)** Length of hospital stay. **(E)** Operative time. **(F)** Mortality. **(G)** Complication.

**Figure 3:**
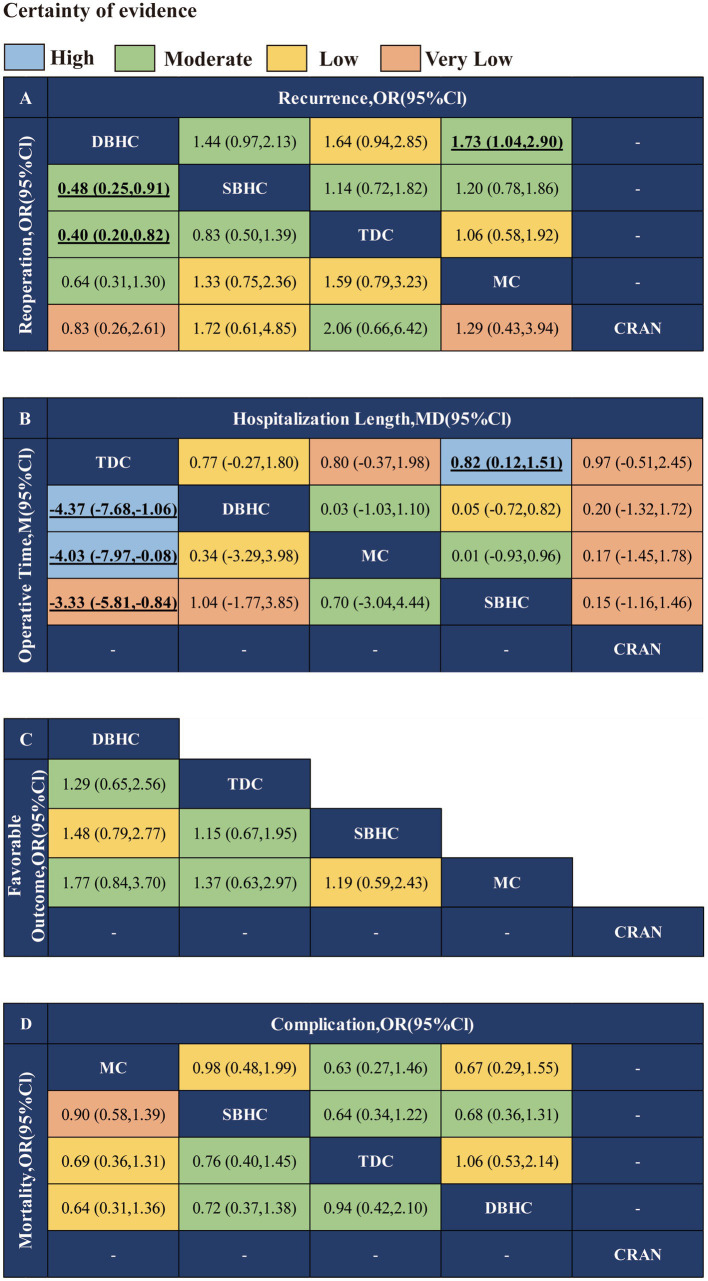
Network meta-analysis results of different surgical treatments for CSDH. **(A)** Recurrence and reoperation. **(B)** Operative time and length of hospital stay. **(C)** Favorable outcome. **(D)** Mortality and complication.

The network plot of each indicator is shown in Figure 2. DBHC was associated with a significantly lower incidence of recurrence than mini-craniotomy (OR = 0.58, 95% CI: 0.35, 0.97) and a lower incidence of reoperation than SBHC (OR = 0.48, 95% CI: 0.25, 0.91) and TDC (OR = 0.40, 95% CI: 0.20, 0.82). Operative time was shorter with TDC than with SBHC (MD = −2.32; 95% CI: −3.78, −0.86), DBHC (MD −3.61; 95% CI: −5.55, −1.67), and mini-craniotomy (MD = −3.39; 95% CI: −5.70, −1.08). Length of hospital stay was shorter with TDC than with SBHC (MD = −0.82; 95% CI: −1.51, −0.12). There were no differences in the rate of favorable outcomes, mortality, and complications across groups (Figure 3; Supplementary Figures S19–S25).

### 3.5. SUCRA and rank probability

**Figure 4:**
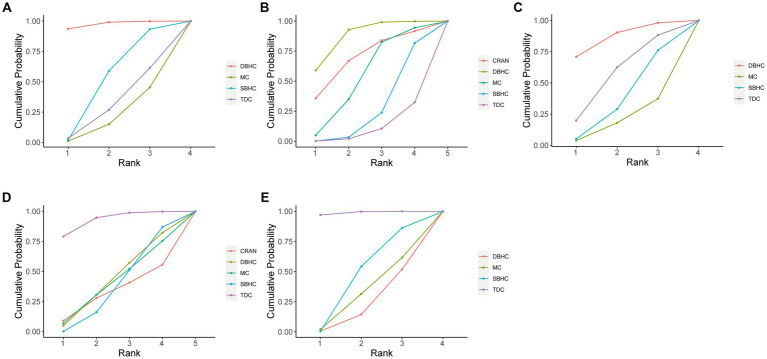
Cumulative probability of each intervention for efficacy outcomes. A larger SUCRA value indicated a better rank for the intervention. **(A)** Recurrence. **(B)** Reoperation. **(C)** Favorable outcome. **(D)** Length of hospital stay. **(E)** Operative time.

**Figure 5:**
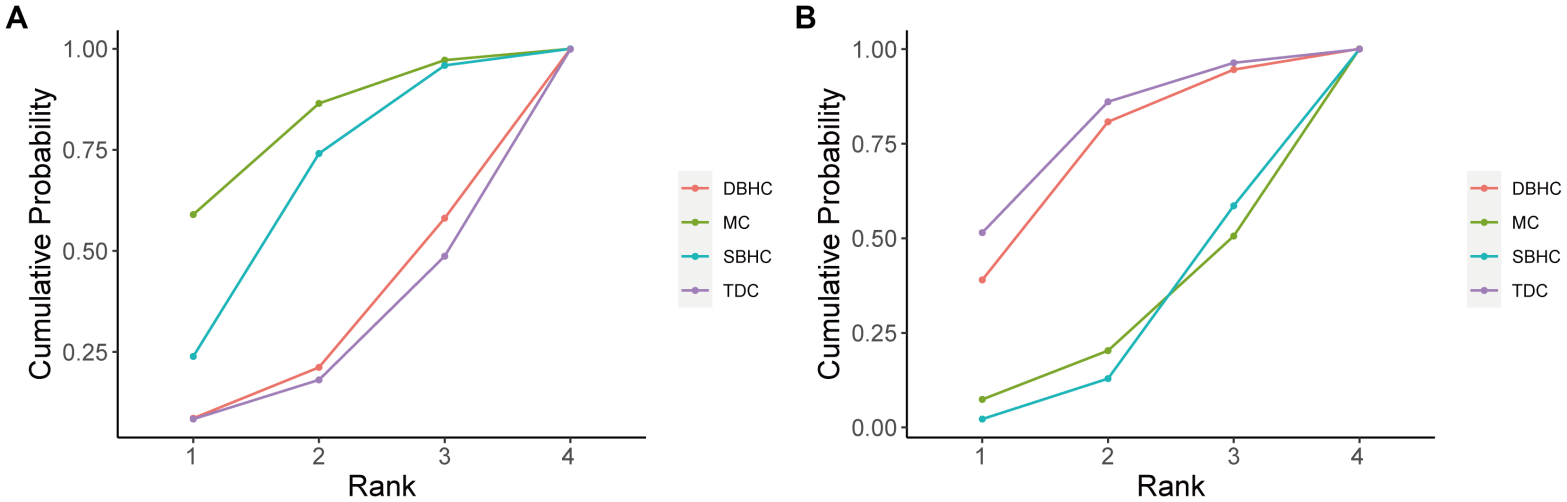
Cumulative probability of each intervention for safety outcomes. A larger SUCRA value indicated a better rank for the intervention. **(A)** Mortality. **(B)** Complication.

The probability ranking of each treatment strategy according to different indicators was analyzed by calculating SUCRA. For recurrence rate, DBHC achieved the highest SUCRA value (96.3%), followed by SBHC (52%), TDC (29.6%), and mini-craniotomy (22.1%; Figure 4). DBHC also had the highest SUCRA for rate of reoperation (87.4%) and favorable outcome (86.2%) among treatments. For operative time, TDC ranked first (99.9%), followed by SBHC (59.2%), mini-craniotomy (24.7%), and DBHC (16.2%). TDC had the highest SUCRA for length of hospital stay (93.1%). Mini-craniotomy (79.8%) and TDC (78.1%) had the highest ranking probabilities for mortality and complication rates, respectively (Figure 5).

### 3.6. Subgroup analysis

Subgroup analysis was conducted on surgical techniques with closed-system drainage. DBHC still had the highest SUCRA value in recurrence (97.1%) and reoperation (86.9%). However, no statistical difference was observed in recurrence and reoperation between DBHC and SBHC with drainage, while DBHC remained superior to TDC in reoperation rate (OR = 0.44, 95% CI: 0.20, 0.96). For length of hospitalization and operative time, TDC still had the highest ranking probability. The results of favorable outcomes, complications, and mortality among surgeries did not change when closed-system drainage was used (Supplementary Figure S47).

### 3.7. Heterogeneity, inconsistency, and convergence analysis

There was low heterogeneity in mortality rates (I^2^ = 0%) across studies, and moderate heterogeneity in recurrence (I^2^ = 53.4%), reoperation (I^2^ = 52.1%), favorable outcome (I^2^ = 54.9%), and complication (I^2^ = 69.8%) rates. Meanwhile, significant heterogeneity was detected in length of hospital stay (I^2^ = 91.0%) and operative time (I^2^ = 99.5%; Supplementary Table S5). We used a node-splitting model to assess differences between direct and indirect comparisons and determine the consistency of 3 networks with an I^2^ value > 70% (e.g., hospitalization length, complication rate, and operative time). There was no evidence of inconsistency in any outcome measure (Supplementary Figures S5–S11). We confirmed the effective convergence of the model by observing that the Potential Scale Reduction Factor values for all parameters were constrained to 1 (Supplementary Figures S26–S32). Without any discernible individual chain fluctuations, the normally distributed density map suggested excellent convergence of these models (Supplementary Figures S33–S39).

### 3.8. Quality assessment and risk of bias

The quality of evidence of included studies was evaluated according to GRADE criteria (Supplementary Table S4). In the risk of bias assessment, the study by Goyal et al. showed unclear risk of bias (24), whereas the one by D’oria et al. showed high risk of bias in random sequence generation (25). Three studies showed unclear risk of bias in allocation concealment (25–27). For the blinding of participants and personnel, the risk of bias was unclear in 2 studies (26, 28) and high in 2 others (29); and for the blinding of outcome assessments the risk of bias was unclear in 4 studies (28–30) (Supplementary Figures S1, S2). The overall quality of included retrospective studies was not extremely high (Supplementary Figures S3, S4). The distribution of data points in the funnel plots was relatively symmetric, suggesting that there was no significant publication bias affecting the results of the NMA (Supplementary Figures S12–S18).

## 4. Discussion

Surgery plays a key role in CSDH management, particularly in patients with significant clinical symptoms such as disturbance of consciousness and reduced muscle strength and limb sensation (31). However, because of a lack of consensus regarding the optimal treatment modality for CSDH, clinicians usually base their decision on clinical experience or the patient’s conditions (1). As each technique has advantages and disadvantages, we conducted a NMA to compare the efficacy and safety of the 5 most commonly used approaches (SBHC, DBHC, TDC, mini-craniotomy, and craniotomy). For efficacy outcomes, DBHC was associated with lower incidences of recurrence and reoperation, whereas TDC was superior to the other modalities in temporal outcome measures such as operative time. Although no statistically significant differences were observed in safety outcomes, TDC ranked first in terms of avoiding the occurrence of complications, and mini-craniotomy ranked first for avoiding mortality.

Recurrence and reoperation rates were the main variables for assessing the efficacy of the surgical methods. DBHC had the highest SUCRA value in recurrence rate and also showed a significantly lower recurrence rate than mini-craniotomy, consistent with a previous study (32). Two other retrospective studies concluded that the incidence of recurrence was lower with SBHC than with mini-craniotomy, although the difference was nonsignificant (33, 34). However, several studies have reported lower recurrence rates with mini-craniotomy than with BHC, mainly because of ease of visualization and ability to address intracapsular septations and organized clots (9, 35). No other differences were observed between treatments. BHC was first described in 1964 and is performed with 1 or 2 holes. However, there are no specific indications for the number of holes that are needed to achieve optimal surgical results (36–38). In our analysis, there was no significant difference between SBHC and DBHC in recurrence rate, which was in line with findings from 2 meta-analyses (32, 36). The main reasons for recurrence of CSDH are hematoma membrane remnants and reaccumulation of subdural fluid caused by the residual hematoma or rebleeding (39). Some investigators have suggested that because evacuating hematoma fluid is easier with DBHC than with SBHC, the rate of recurrence is lower with the former technique (38, 40). There is no consensus on whether the number of holes is an independent predictor of recurrence (38, 41, 42). However, it was demonstrated that hematomas could be adequately evacuated by SBHC, given that all hematoma cavities are continuous and interconnected (43). Additionally, neurosurgeons did not randomly select the number of holes in most studies and will make the decision based on the patient’s condition. For instance, they may avoid using DBHC in patients with bleeding risk factors such as coagulopathies that could affect the outcome of recurrence (44). Weigel et al. (45) reported that TDC was inferior to BHC and craniotomy in terms of recurrence rate, but we did not observe any differences in our study. Another study found that recurrence rates did not differ significantly between TDC and BHC and suggested that adequate decompression supplemented with postoperative drainage is critical for a good clinical outcome (46).

The development of severe complications and reaccumulation of severe hematoma that aggravates the patient’s clinical symptoms necessitates reoperation. In this study, DBHC was associated with a lower rate of reoperation compared with SBHC and TDC and ranked first in cumulative probability. By using imaging to precisely locate a hematoma with small septa, CSDH can be treated with 2 holes, which can more effectively evacuate the hematoma and reduce the risk of reoperation for recurrent or residual CSDH (36). However, it should be noted that in a subgroup analysis of surgical management with closed system drainage, there was no statistical difference between SBHC and DBHC. Alcalá-Cerra et al. also concluded that the use of subdural drainage could significantly prevent recurrence and reduce the rate of reoperation (47). Therefore, postoperative drainage might be one of the primary factors for patients to achieve greater clinical outcomes, rather than the number of holes (48). It was previously demonstrated that the reoperation rate was higher with TDC than with BHC, although the difference was not statistically significant (46). The high reoperation rate with TDC may be attributed to residual hematoma, as the postoperative residual hematoma volume was found to be significantly greater with TDC than with DBHC (49), possibly because the tube used in TDC is much smaller, which can increase the risk of blockage; moreover, the small hole for TDC does not allow the direction of the drainage tube to be adjusted when blockage occurs.

For temporal outcome measures, TDC ranked first for operative time owing to the convenience of the bedside procedure that uses only a simple treatment towel, electric drill, and minimally invasive cone-hole needle. Drainage can be performed immediately, thereby rapidly reducing intracranial pressure; thus, TDC can be used for emergency treatment or in high-risk patients with nonseptated CSDH (50). TDC also had a higher rank probability than SBHC for length of hospital stay, consistent with a previous study (51). However, caution is needed when comparing of the efficacy of the different surgical techniques based on time variables because of the high heterogeneity among these and other variables such as patient (e.g., volume and location of hematoma, age, and comorbidities) and hospital (e.g., surgical procedures, surgeon level, and nursing care) characteristics across retrospective studies.

Although different definitions were used in the included studies, a favorable outcome was generally considered as one that avoided reoperation, death, and severe surgical or medical complications. According to the SUCRA, DBHC had the highest probability of a favorable outcome, followed by TDC, mini-craniotomy, and SBHC. The purpose of surgical treatment for CSDH is to reduce the space-occupying effect of the hematoma through effective evacuation. The surgical procedures included in the NMA are not technically difficult for most neurosurgeons, and CSDH is not usually life-threatening. Thus, most patients can achieve a favorable outcome even if they experience postoperative complications or require reoperation.

Postoperative complications and recurrence are important considerations when selecting the optimal surgical management strategy for CSDH. In the present analysis, TDC was associated with the lowest incidence of complications, although this did not differ significantly from other treatments. A previous study found that TDC may be associated with more complications because it is performed at the bedside with a longer drainage time than BHC, which increases the risk of intracranial infection or hypostatic pneumonia (52). However, these risks have been minimized with the modification of the surgical technique (3). Additionally, although there was no significant difference in complication and recurrence rates between SBHC, DBHC, and mini-craniotomy, one study reported that mini-craniotomy was associated with medical and severe surgical complications (43). The comparison of mortality rates yielded results similar to those observed for complication rates. One study found that the mortality rate was higher with craniotomy (12.2%) than with BHC (3.7%) or TDC (5.1%), but the difference was nonsignificant (50). The finding that mortality rates were low and did not differ significantly between different procedures is consistent with the routine nature of the surgical approaches used for CSDH and the fact that the condition itself is not life-threatening.

From the perspective of surgical effectiveness and safety, we discussed the best surgical approach based on the seven aspects mentioned above. However, it is also essential to select the appropriate surgical strategy based on the levels of organization of the hematoma. For nonseptated and predominantly liquified CSDH, the use of TDC or BHC is essential (7). Meanwhile, Strong evidences support the use of craniotomy as the optimal treatment strategy for conditions such as organized chronic subdural hematomas, the existence of a solid hematoma, failure of brain reexpansion, or pronounced swelling adjacent to the hematoma (53–55).

There were several limitations to this NMA. (1) There was significant heterogeneity across studies, which was largely attributable to the fact that the NMA was based on RCTs and retrospective studies with a low strength of evidence according to GRADE criteria. (2) The effect of other factors (e.g., hematoma volume and location, follow-up period, or postoperative drug treatment) could not be evaluated, and because retrospective studies were included, potentially confounding effects of variables such as age, sex, anticoagulation, etc. on the outcomes could not be eliminated. (3) As there were few RCTs of newer surgical techniques, such as middle meningeal artery embolization and endoscopic treatment, these modalities were not included in the analysis. (4) Several outcomes reported in this study were not standardized. Singla et al. (56) addressed this issue by distinguishing between recurrence, reoperation, and first operation failure. However, most studies do not consider the influence of operation failure on the risk of reoperation, and misinterpretation of reoperation and recurrence may exaggerate the rate of CSDH recurrence (57). (5) Surgical techniques and procedures are not always performed consistently between and within centers, which could influence the outcome. (6) The aforementioned outcomes are specific to the initial treatment of CSDH and may not be applicable for recurrent CSDH. Currently, craniotomy is recommended for recurrent CSDH (47, 58, 59). However, further studies are still needed. Despite these limitations, the results of this NMA provide the best available evidence regarding the efficacy and safety of 5 commonly used surgical treatments for CSDH.

## 5. Conclusion

In conclusion, the results of the NMA showed that DBHC is superior to other surgical approaches for CSDH based on the low rates of recurrence and reoperation. Although safety outcomes did not differ significantly across surgical techniques, DBHC had the highest rank probability. There were only small differences in duration of hospitalization, favorable outcome, and complication rate between TDC and DBHC. In emergency situations or in a primary care center, TDC is easier to perform than DBHC, whereas, in areas with adequate medical resources, DBHC may be a better choice. However, additional multicenter and high-quality studies are needed to identify the surgical modality for CSDH that yields the best clinical outcome.

## Data availability statement

The original contributions presented in the study are included in the article/Supplementary material, further inquiries can be directed to the corresponding authors.

## Author contributions

ZW and WG designed the study and developed the analysis plan. YQ, MX, and ZY analyzed the data and performed the meta-analysis. YQ and MX contributed to the writing of the article. AD, MW, XC, and ZC revised the manuscript and polished the language. All authors contributed to the article and approved the submitted version.

## Funding

This work was supported by Suzhou Health Talents Training Project (grant no GSWS2019002) and Suzhou Science and Technology Development Plan Projects (SS202057).

## Conflict of interest

The authors declare that the research was conducted in the absence of any commercial or financial relationships that could be construed as a potential conflict of interest.

## Publisher’s note

All claims expressed in this article are solely those of the authors and do not necessarily represent those of their affiliated organizations, or those of the publisher, the editors and the reviewers. Any product that may be evaluated in this article, or claim that may be made by its manufacturer, is not guaranteed or endorsed by the publisher.
