# Supplementary material for: Comparison of different surgical techniques for chronic subdural hematoma: a network meta-analysis
Source: Front Neurol. 2023 Jul 26;14:1183428. doi: 10.3389/fneur.2023.1183428 (PMC10411900; doi:10.3389/fneur.2023.1183428)

Supplementary appendix

Comparison of Different Surgical Techniques for Chronic Subdural Hematoma: A Network Meta-Analysis

**Table legends**

Table S1: The detailed search strategy.

Table S2: Summary and detailed effects sizes from pair-wise meta-analysis of efficacy outcomes; from all trials using random effects models.

Table S3: Inclusion, exclusion criteria, study design and outcome assessments of the included

Table S4: Detailed certainty of evidence for each outcome in league table.

Table S5: Heterogeneity of the network meta-analysis

**Figure legends**

Figure S1 Risk of bias assessment of RCT with ROB.

Figure S2 Summary of risk of bias assessment of RCT with ROB.

Figure S3 Risk of bias assessment of retrospective studies with ROBINS-I.

Figure S4 Summary of risk of bias assessment of retrospective studies with ROBINS-I.

Figure S5: Forest plots for the inconsistency: Recurrence.

Figure S6: Forest plots for the inconsistency: Reoperation.

Figure S7: Forest plots for the inconsistency: Favorable outcome.

Figure S8: Forest plots for the inconsistency: LOS.

Figure S9 Forest plots for the inconsistency: Operation time.

Figure S10 Forest plots for the inconsistency: Complication.

Figure S11 Forest plots for the inconsistency: Mortality.

Figure S12: Funnel plot for Recurrence.

Figure S13: Funnel plot for Reoperation.

Figure S14: Funnel plot for Favorable outcome.

Figure S15: Funnel plot for LOS.

Figure S16: Funnel plot for Operation time.

Figure S17: Funnel plot for Complication.

Figure S18: Funnel plot for Mortality.

Figure S19: Forest plots of the network meta-analysis: Recurrence.

Figure S20: Forest plots of the network meta-analysis: Reoperation.

Figure S21: Forest plots of the network meta-analysis: Favorable outcome.

Figure S22: Forest plots of the network meta-analysis: LOS.

Figure S23: Forest plots of the network meta-analysis: Operation time.

Figure S24: Forest plots of the network meta-analysis: Complication.

Figure S25: Forest plots of the network meta-analysis: Mortality.

Figure S26: Convergence diagnostics of the network meta-analysis: Recurrence.

Figure S27: Convergence diagnostics of the network meta-analysis: Reoperation.

Figure S28: Convergence diagnostics of the network meta-analysis: Favorable outcome.

Figure S29: Convergence diagnostics of the network meta-analysis: LOS.

Figure S30: Convergence diagnostics of the network meta-analysis: Operation time.

Figure S31: Convergence diagnostics of the network meta-analysis: Complication.

Figure S32: Convergence diagnostics of the network meta-analysis: Mortality.

Figure S33: Trace and density of the network meta-analysis: Recurrence.

Figure S34: Trace and density of the network meta-analysis: Reoperation.

Figure S35: Trace and density of the network meta-analysis: Favorable outcome.

Figure S36: Trace and density of the network meta-analysis: LOS.

Figure S37: Trace and density of the network meta-analysis: Operation time.

Figure S38: Trace and density of the network meta-analysis: Complication.

Figure S39: Trace and density of the network meta-analysis: Mortality.

Figure S40: Forest plots for the heterogeneity: Recurrence.

Figure S41: Forest plots for the heterogeneity: Reoperation.

Figure S42: Forest plots for the heterogeneity: Favorable outcome.

Figure S43: Forest plots for the heterogeneity: LOS.

Figure S44: Forest plots for the heterogeneity: Operation time.

Figure S45: Forest plots for the heterogeneity: Complication.

Figure S46: Forest plots for the heterogeneity: Mortality.

Figure S47: Network meta-analysis results of different Surgical Techniques with drainage for Chronic Subdural Hematoma treatments.

Figure S48: Result of PRISMA checklist.

**Table S1: The detailed search strategy.**

**Pubmed：**

| Search | Query | Results |
| --- | --- | --- |
| #1 | (Hematoma, Subdural, Chronic [MeSH Terms]) | 1,725 |
| #2 | ((((((Subdural Hematoma, Chronic[Title/Abstract]) OR (Chronic Subdural Hematoma[Title/Abstract])) OR (Chronic Subdural Hematomas[Title/Abstract])) OR (Hematoma, Chronic Subdural[Title/Abstract])) OR (Hematomas, Chronic Subdural[Title/Abstract])) OR (Subdural Hematomas, Chronic[Title/Abstract])) OR (Hemorrhage, Subdural, Chronic[Title/Abstract]) | 2,855 |
| #3 | #1 OR #2 | 3,270 |
| #4 | (((haematoma*[Title/Abstract]) OR (hematoma*[Title/Abstract])) OR (haemorrhag*[Title/Abstract])) OR (hemorrhag*[Title/Abstract]) | 349,048 |
| #5 | (((subdural[Title/Abstract]) OR (subepidural[Title/Abstract])) OR (pachymening*[Title/Abstract])) OR (extracran*[Title/Abstract]) | 35,621 |
| #6 | #4 AND #5 | 13,835 |
| #7 | #3 OR #6 | 13,901 |
| #8 | (((((((minicraniotomy[Title/Abstract]) OR (Burr hole craniostomy[Title/Abstract])) OR (double burr hole craniostomy[Title/Abstract])) OR (Single burr hole craniostomy[Title/Abstract])) OR (Twist drill craniostomy[Title/Abstract])) OR (burr hole drainage[Title/Abstract])) OR (single burr hole drainage[Title/Abstract])) OR (double burr hole drainage[Title/Abstract]) | 602 |
| #9 | (Drainage [Mesh]) | 64,483 |
| #10 | (Trephining [Mesh]) | 1,667 |
| #11 | (Craniotomy [Mesh]) | 17,402 |
| #12 | #8 OR #9 OR #10 OR #11 | 81,353 |
| #13 | #7 AND # 12 | 1,814 |
| #14 | #13 AND ((((((((("Comparative Study"[Publication Type]) OR "Randomized Controlled Trial"[Publication Type]) OR "Controlled Clinical Trial"[Publication Type])) OR (((((((randomized[Title/Abstract]) OR randomised[Title/Abstract]) OR placebo[Title/Abstract]) OR randomly[Title/Abstract]) OR trial[Title/Abstract]) OR groups[Title/Abstract]) OR group[Title/Abstract]))) NOT (("Animals"[Mesh]) NOT ("Animals"[Mesh] AND "Humans"[Mesh]))))) | 482 |
| #15 | #13 AND ((((Retrospective Stud*) OR (Cohort Stud*)) OR (Comparative Stud*)) OR ((("Retrospective Studies"[Mesh]) OR "Cohort Studies"[Mesh]) OR "Comparative Study" [Publication Type])) | 797 |
| #16 | #14 OR #15 | 896 |
| #17 | #16 AND (English [Language]) | **815** |

**Embass：**

| Search | Query | Results |
| --- | --- | --- |
| #1 | 'hematoma, subdural, chronic'/exp | 21,764 |
| #2 | extracran*:ti,ab,kw | 24,956 |
| #3 | pachymening*:ti,ab,kw | 1,937 |
| #4 | subepidural:ti,ab,kw | 5 |
| #5 | subdural:ti,ab,kw | 23,822 |
| #6 | #2 OR #3 OR #4 OR #5 | 50,242 |
| #7 | haematoma*:ti,ab,kw | 17,640 |
| #8 | hematoma*:ti,ab,kw | 77,149 |
| #9 | haemorrhag*:ti,ab,kw | 85,047 |
| #10 | hemorrhag*:ti,ab,kw | 339,178 |
| #11 | bleed*:ti,ab,kw | 404,618 |
| #12 | #7 OR #8 OR #9 OR #10 OR #11 | 811,943 |
| #13 | chronic:ti,ab,kw | 1,968,546 |
| #14 | #6 AND #12 AND #13 | 5,221 |
| #15 | #1 OR #14 | 22,463 |
| #16 | minicraniotomy:ti,ab,kw | 148 |
| #17 | 'burr hole craniostomy':ti,ab,kw | 162 |
| #18 | 'double burr hole craniostomy':ti,ab,kw | 3 |
| #19 | 'single burr hole craniostomy':ti,ab,kw | 16 |
| #20 | 'twist drill craniostomy':ti,ab,kw | 103 |
| #21 | 'burr hole drainage':ti,ab,kw | 344 |
| #22 | 'single burr hole drainage':ti,ab,kw | 13 |
| #23 | 'double burr hole drainage':ti,ab,kw | 6 |
| #24 | 'drainage'/exp | 2151 |
| #25 | 'trephining'/exp | 170,552 |
| #26 | 'craniotomy'/exp | 38,334 |
| #27 | #16 OR #17 OR #18 OR #19 OR #20 OR #21 OR #22 OR #23 OR #24 OR #25 OR #26 | 172,949 |
| #28 | #15 AND #27 | 5,230 |
| #29 | #28 AND (‘randomized controlled trial’:de or ‘placebo*’:de or ‘double-blind procedure’:de or ‘single-blind procedure’:de) | 139 |
| #30 | #28 AND ('retrospective study'/exp) | 1,043 |
| #31 | #29 OR #30 | 1,169 |
| #32 | #31 AND english:la | **1,133** |

**Cochrane:**

| Search | Query | Results |
| --- | --- | --- |
| #1 | MeSH descriptor: [Hematoma, Subdural, Chronic] explode all trees | 102 |
| #2 | (extracran*):ti,ab,kw | 914 |
| #3 | (pachymening*):ti,ab,kw | 6 |
| #4 | (subepidural):ti,ab,kw | 0 |
| #5 | (subdural):ti,ab,kw | 778 |
| #6 | #1 OR #2 OR #3 OR #4 OR #5 | 1,681 |
| #7 | (haematoma*):ti,ab,kw | 1,164 |
| #8 | (hematoma*):ti,ab,kw | 6,117 |
| #9 | (haemorrhag*):ti,ab,kw | 6,122 |
| #10 | (hemorrhag*):ti,ab,kw | 32,724 |
| #11 | (bleed*):ti,ab,kw | 50,744 |
| #12 | #7 OR #8 OR #9 OR #10 OR #11 | 76,572 |
| #13 | #6 AND #12 | 883 |
| #14 | (minicraniotomy):ti,ab,kw | 5 |
| #15 | (Burr hole craniostomy):ti,ab,kw | 21 |
| #16 | (double burr hole craniostomy):ti,ab,kw | 1 |
| #17 | (Single burr hole craniostomy):ti,ab,kw | 6 |
| #18 | (Twist drill craniostomy):ti,ab,kw | 10 |
| #19 | (burr hole drainage):ti,ab,kw | 69 |
| #20 | (single burr hole drainage):ti,ab,kw | 20 |
| #21 | (double burr hole drainage):ti,ab,kw 4 | 4 |
| #22 | MeSH descriptor: [Drainage] explode all trees | 3,037 |
| #23 | MeSH descriptor: [Trephining] explode all trees | 16 |
| #24 | MeSH descriptor: [Craniotomy] explode all trees | 480 |
| #25 | #14 OR #15 OR #16 OR #17 OR #18 OR #19 OR #20 OR #21 OR #22 OR #23 OR #24 | 3,554 |
| #26 | #13 AND #25 | **103** |

**Table S2: Summary and detailed effects sizes from pair-wise meta-analysis of efficacy outcomes; from all trials using random effects models.**

**Table S2a.Recurrence**

| Comparisons | No.of studies | No.of patients | Pairwise meta-analysis odds ratio(95%CI) | Heterogeneity I^2^(variation in OR attributable to heterogeneity) | P value |
| --- | --- | --- | --- | --- | --- |
| SBHC vs DBHC | 12 | 2135 | 1.42 [0.94, 2.16] | 41 | P=0.10 |
| SBHC vs TDC | 11 | 1195 | 0.96 [0.47, 1.97] | 63 | P=0.91 |
| SBHC vs MC | 7 | 2375 | 0.74 [0.52, 1.06] | 44 | P=0.10 |
| DBHC vs TDC | 4 | 403 | **0.38 [0.18, 0.80]** | 0 | P=0.01 |
| DBHC vs MC | 4 | 515 | 0.78 [0.35, 1.73] | 53 | P=0.54 |
| TDC vs MC | 1 | 166 | 0.62 [0.27, 1.44] | NA | P=0.27 |

**Table S2b.Reoperation**

| Comparisons | No.of studies | No.of patients | Pairwise meta-analysis odds ratio(95%CI) | Heterogeneity I^2^(variation in OR attributable to heterogeneity) | P value |
| --- | --- | --- | --- | --- | --- |
| SBHC vs DBHC | 4 | 317 | **2.79 [1.03, 7.58]** | 50 | P=0.04 |
| SBHC vs TDC | 10 | 1130 | 0.75 [0.36, 1.58] | 60 | P=0.46 |
| SBHC vs MC | 4 | 1718 | 1.02 [0.67, 1.54] | 33 | P=0.92 |
| SBHC vs CRAN | 1 | 299 | **2.28 [1.07, 4.85]** | NA | P=0.03 |
| DBHC vs TDC | 3 | 303 | **0.40 [0.18, 0.90]** | 0 | P=0.03 |
| DBHC vs MC | 3 | 384 | 1.01 [0.39, 2.64] | 49 | P=0.98 |
| DBHC vs CRAN | 1 | 51 | 0.63 [0.13, 2.97] | NA | P=0.55 |
| TDC vs MC | 1 | 166 | 1.61 [0.70, 3.71] | NA | P=0.27 |
| MC vs CRAN | 1 | 134 | 0.74 [0.19, 2.92] | NA | P=0.67 |

**Table S2c.Favorable outcome**

| Comparisons | No.of studies | No.of patients | Pairwise meta-analysis odds ratio(95%CI) | Heterogeneity I^2^(variation in OR attributable to heterogeneity) | P value |
| --- | --- | --- | --- | --- | --- |
| SBHC vs DBHC | 3 | 450 | 0.70 [0.38, 1.29] | 22 | P=0.25 |
| SBHC vs TDC | 8 | 1002 | 0.72 [0.37, 1.41] | 50 | P=0.34 |
| SBHC vs MC | 2 | 286 | **1.96 [1.10, 3.48]** | 0 | P=0.02 |
| DBHC vs TDC | 3 | 301 | 1.68 [0.90, 3.13] | 0 | P=0.10 |
| DBHC vs MC | 3 | 436 | 1.55 [0.51, 4.73] | 67 | P=0.44 |
| TDC vs MC | 1 | 166 | 0.71 [0.36, 1.42] | NA | P=0.33 |

**Table S2d.Length of hospital stay**

| Comparisons | No.of studies | No.of patients | Pairwise meta-analysis mean difference (95%CI) | Heterogeneity I^2^(variation in MD attributable to heterogeneity) | P value |
| --- | --- | --- | --- | --- | --- |
| SBHC vs DBHC | 3 | 337 | 4.15 [-6.31, 14.62] | 46 | P=0.44 |
| SBHC vs TDC | 4 | 335 | **3.08 [1.76, 4.39]** | 40 | P<0.0001 |
| SBHC vs MC | 2 | 130 | **-5.40 [-9.94, -0.86]** | 95 | P=0.55 |
| SBHC vs Craniotomy | 1 | 299 | 8.91 [-20.19, 38.01] | NA | P=0.19 |
| DBHC vs MC | 1 | 62 | 9.02 [-4.22, 22.26] | NA | P=0.18 |

**Table S2e.Operation time**

| Comparisons | No.of studies | No.of patients | Pairwise meta-analysis mean difference (95%CI) | Heterogeneity I^2^(variation in MD attributable to heterogeneity) | P value |
| --- | --- | --- | --- | --- | --- |
| SBHC vs DBHC | 3 | 459 | **-27.75 [-30.30, -25.19]** | 16 | P<0.0001 |
| SBHC vs TDC | 4 | 378 | **28.46 [19.01, 37.92]** | 97 | P<0.0001 |
| SBHC vs MC | 1 | 55 | **-39.41 [-54.01, -24.81]** | NA | P<0.0001 |
| DBHC vs TDC | 1 | 161 | **33.00 [27.07, 38.93]** | NA | P<0.0001 |
| DBHC vs MC | 2 | 225 | **-13.40 [-21.41, -5.38]** | 0 | P=0.001 |
| TDC vs MC | 1 | 166 | **-56.32 [-66.47, -46.18]** | NA | P<0.0001 |

**Table S2f.Complication**

| Comparisons | No.of studies | No.of patients | Pairwise meta-analysis odds ratio(95%CI) | Heterogeneity I^2^(variation in OR attributable to heterogeneity) | P value |
| --- | --- | --- | --- | --- | --- |
| SBHC vs DBHC | 6 | 783 | 1.49 [0.78, 2.84] | 4 | P=0.12 |
| SBHC vs TDC | 8 | 1002 | 2.17 [0.97, 4.84] | 40 | P<0.0001 |
| SBHC vs MC | 4 | 1608 | 0.79 [0.27, 2.28] | 86 | P<0.0001 |
| DBHC vs TDC | 4 | 403 | 0.77 [0.47, 1.27] | 0 | P=0.31 |
| DBHC vs MC | 2 | 225 | 1.69 [0.43, 6.71] | 45 | P=0.39 |
| TDC vs MC | 1 | 166 | 1.47 [0.79, 2.73] | NA | P=0.22 |

**Table S2g.Mortality**

| Comparisons | No.of studies | No.of patients | Pairwise meta-analysis odds ratio(95%CI) | Heterogeneity I^2^(variation in OR attributable to heterogeneity) | P value |
| --- | --- | --- | --- | --- | --- |
| SBHC vs DBHC | 5 | 677 | 1.23 [0.49, 3.08] | 0 | P=0.62 |
| SBHC vs TDC | 7 | 882 | 0.75 [0.36, 1.56] | 0 | P=0.44 |
| SBHC vs MC | 4 | 2102 | 0.99 [0.54, 1.81] | 60 | P=0.59 |
| DBHC vs TDC | 3 | 301 | 0.73 [0.16, 3.30] | 41 | P=0.45 |
| DBHC vs MC | 3 | 586 | **2.33 [1.10, 4.96]** | 0 | P=0.02 |
| TDC vs MC | 1 | 166 | 0.68 [0.11, 4.15] | NA | P=0.67 |

**Table S3: Inclusion, exclusion criteria, study design and outcome assessments of the included studies.**

| **Trials** | D’ORIA et al. 2020 |
| --- | --- |
| ***Inclusion Criteria*** | Patients with unilateral CSDH, midline shift and/or neurological impairment due the subdural hematoma were admitted in our clinic and recruited into the study between March 2013 and January 2017. |
| ***Exclusion Criteria*** | Patients declined to participate in the study or were diagnosed with bilateral CSDH between March 2013 and January 2017. |
| ***Study Design*** | This was a prospective, randomized, double-blind, SBHC vs DBHC, 2 comparison group study of the efficacy and safety of surgical treatments in adults with chronic subdural hematoma. The first 120 consecutive patients were included in group I and treated by SBHC, while the following consecutive 120 patients were allocated in group II and underwent the DBHC. |
| ***Efficacy Outcomes*** | The recurrence rate after surgery, average thickness of residual hematoma, average duration of operation, mean of postoperative Mgs, mean of hospitalization length after surgery, mean thickness of residual subdural fluid collection, Mean hospitalization time, MGS and mean operative time |
| ***Safety Outcomes*** | Mortality rate and complication (including average volume of pneumocrania, infection). |

| **Trials** | Nayıl1 et al. 2014 |
| --- | --- |
| ***Inclusion Criteria*** | All patients with a CT diagnosis of subacute and chronic subdural hematomas were enrolled in the study between July 2010 to December 2011 in the Department of Neurosurgery at Sher-i-Kashmir Institute of Medical Sciences, Kashmir, India. |
| ***Exclusion Criteria*** | Patients were diagnosed multilocular collections in the chronic subdural hematoma |
| ***Study Design*** | This was a prospective, prospective randomized controlled trial. There were 130 patients who received single-burr hole and 124 patients who received double-burr hole for drainage. |
| ***Efficacy Outcomes*** | The Recurrence rate after surgery |

| **Trials** | Rafi et al. 2017 |
| --- | --- |
| ***Inclusion Criteria*** | 1) patients from both genders, 2) patients suffering from chronic subdural hematoma with burr hole treatment indication, 3) informed consent to take part in the research. |
| ***Exclusion Criteria*** | 1) those patients for whom craniotomy is prescribed in order to treat chronic subdural hematoma, 2) background disorders such as underlying brain aneurysm, active leukemia, Ventriculoperitoneal Shunt, 3) disagreement to take part in the research, and 4) existence of underlying coagulation disorder. |
| ***Study Design*** | This was a randomized, double-blind, one burr hole vs two burr holes, 2 comparison group study of the efficacy and safety of surgical treatments in adults with chronic subdural hematoma. |
| ***Efficacy Outcomes*** | Secondary operation rate, GCS 48 hours after operation, hematoma level 48 hours after operation and mean hospitalization time. |
| ***Safety Outcomes*** | Mortality rate and Complication (including average volume of pneumocrania, infection). |

| **Trials** | Goyal et al. 2018 |
| --- | --- |
| ***Inclusion Criteria*** | Persons in the age group of 18–90 years with CSDH on computed tomography (CT) scan/magnetic resonance imaging (MRI) scan |
| ***Exclusion Criteria*** | Patients younger than 18 years of age, CSDH with thick calcified membrane requiring craniotomy and excision of the thick calcified membrane, ipsilateral recurrent CSDH, bilateral CSDH, cerebrospinal fluid shunt in situ, subdural hygroma, and subdural empyema. |
| ***Study Design*** | This was a prospective, randomized, controlled study and was conducted in the Department of Neuro Surgery, Army Hospital (Research and Referral), Delhi Cantt, New Delhi, over two calendar years between May 2, 2011 and May 1, 2013. Forty consecutive cases with CSDH were included and divided randomly into two groups, one which was treated with BHE and the other with TDE. |
| ***Efficacy Outcomes*** | The primary outcome variable studied was clinically significant recurrence rate that means need for reaspiration/redo surgery which is defined as: before discharge if patient does not reach Markwalder neurological grading score (MGS) grade 0 or 1 and CT scan shows residual fluid or air more than 10 mm thick with any midline shift (MLS), or after discharge if there is a recurrence of or increase in symptoms (headache, altered mentation, and hemiparesis) and CT scan shows residual fluid or air >10 mm thick with any MLS. The secondary outcome include: Glasgow coma scale (GCS) and Markwalder grade at discharge. |
| ***Safety Outcomes*** | Development of postoperative complication (namely, seizures, infection, tension pneumocephalus, and brain injury), Operative mortality rate (death within 30 days of surgery attributable to surgical procedure). |

| **Trials** | Gokmen et al. 2008 |
| --- | --- |
| ***Inclusion Criteria*** | Between November 2002 and April 2006, 70 patients (54 male and 16 female) with unilateral hemispheric CSDH with neurological signs and / or symptoms were operated in the Department of Neurosurgery in our hospital. |
| ***Exclusion Criteria*** | NA |
| ***Study Design*** | This study was planned as a prospective, randomized, controlled trial with the permission of the local ethics commission. Thirty-two patients underwent BHC and TDC was performed for another 38 cases at random. A list to allocate patients by simple randomization was constructed with a table of random numbers. Chi-square tests, independent samples t -test and confidence intervals were used to compare the two groups statistically. |
| ***Efficacy Outcomes*** | Neurological states of the patients were classified according to Markwalder’s grading system, the Glasgow Coma Scale (GCS) was used to define the level of consciousness. The reoperation ,cured, improved morbidty rate to their duration of hospitalization and discharged for 1,3,6 months. |
| ***Safety Outcomes*** | Mortality rate and Complication rate during hospitalization and out-patient period, 1st, 3st, 6st month. |

| **Trials** | Xu et al. 2018 |
| --- | --- |
| ***Inclusion Criteria*** | Patients diagnosed with CSDH via head CT or magnetic resonance imaging (MRI) at Huai'an First  People's Hospital (Huai'an China) between January 2016 and January 2017 were enrolled in the present study. Inclusion criteria were a clear correlation of CSDH with neurologic impairment symptoms and signs confirmed via neurological examination; the requirement of hematoma drainage and decompression; and an age >18 years for either sex. |
| ***Exclusion Criteria*** | Patients with CSDH caused by systemic diseases were excluded, and surgical contraindications were excluded through appropriate biochemical examinations, electrocardiogram and chest CT. |
| ***Study Design*** | The present randomized controlled study investigated the differences in the curative effects of twist-drill craniotomy (TDC) and burr-hole craniotomy (BHC) in the treatment of chronic subdural hematoma (CSDH). A total of 40 patients were enrolled in the present study, and were randomly divided into a TDC group (n=20) and a BHC group (n=20). Three clinicians were independently responsible for the grouping, surgery and follow-up, respectively. |
| ***Efficacy Outcomes*** | The modified Rankin scale (mRS) scores of patients were recorded prior to the operation, and at 48 h and 3 months after the operation. The average length of stay at the hospital (LOS). The recurrence of CSDH is defined as the repeated accumulation of hematoma in the ipsilateral subdural hematoma cavity confirmed by imaging after the initial treatment. Repeated treatment by decompression drainage in the case of CSDH recurrence with symptoms or aggravated symptoms is known as reoperation. patients who underwent a second surgery, namely a remedial operation and reoperation, and who died during follow-up, were not deemed as being cured. |
| ***Safety Outcomes*** | Mortality rate. |

| **Trials** | Duerinck et al. 2022  (NCT02655445) |
| --- | --- |
| ***Inclusion Criteria*** | Patients aged 18 years or older with a CSDH requiring surgical drainage and without contra-indication for use of any of the 3 techniques or for general anesthesia were eligible for inclusion. |
| ***Exclusion Criteria*** | NA |
| ***Study Design*** | We set up a prospective multicenter randomized trial that allocates patients with a CSDH to 1 of 3 surgical intervention types in a 1:1:1 fashion (ClinicalTrials.gov: NCT02655445). The study was conducted between 2012 and 2019 in 4 university hospitals in Belgium (UZ Brussel/VUB, Erasme Hospital/ULB, UZ Leuven/KUL and St-Luc Hospital/UCL). The study protocol was approved by the Ethics committees of all involved hospitals. |
| ***Efficacy Outcomes*** | Clinical data included relevant medical history, neurological examination, Markwalder score, and modified Rankin scale score (mRS). Reoperation was defined as a necessary reintervention because of an inadequate clinical or radiological result of the first surgery, outcome was described as “good” when the patient did not suffer a major medical or surgical complication, did not require reoperation, and did not die as a result of the treatment. |
| ***Safety Outcomes*** | Mortality was described as related if it occurred as a direct consequence of the surgical intervention or a complication thereof. A medical complication was defined as any nonsurgical complication occurring during the hospital stay after the surgery and was considered minor if complete recovery was to be expected (eg, electrolyte disturbances or urinary tract infection). Surgical complications were defined as every complication directly related to the surgery. Epileptic seizures in the postoperative period were considered separately from medical or surgical complications. |

| **Trials** | Sale et al. 2020 |
| --- | --- |
| ***Inclusion Criteria*** | All patients with imaging diagnosis of subacute or chronic subdural hematoma were enrolled in the study. |
| ***Exclusion Criteria*** | NA |
| ***Study Design*** | This is a randomized controlled study over a period of 5 years from January 2015 to December 2019 in a neurosurgical unit in Kaduna, Nigeria that allocates patients with a CSDH to single or double burr hole surgical intervention. Numbers were allocated to each patient after the decision to operate was made. Random sequences were initially generated for each treatment arm and concealed. |
| ***Efficacy Outcomes*** | Clinical parameters used for recurrence include reappearance of the initial presenting symptoms and signs, altered level of consciousness, headaches, convulsions, or neurologic deficits. Only patients with recurrent symptoms had a brain computed tomography scan for confirmation. |
|  |  |

| **Trials** | Jang et al. 2015 |
| --- | --- |
| ***Inclusion Criteria*** | Patients who had underwent BHD following diagnosis of CSDH between January 2010 and June 2014 |
| ***Exclusion Criteria*** | Patients were excluded because they involved a prior history of treatment for cerebral infarction or cerebral hemorrhage such as subarachnoid hemorrhage, or they received ventriculo-peritoneal shunt surgery, or due to a lack of follow-up for a minimum of 3 months. |
| ***Study Design*** | This was a retrospective review of patients who had underwent BHD following diagnosis of CSDH. Comparisons were made after dividing the patients into three groups based on the surgical technique: single BHD without saline irrigation (Group A, n=31), single BHD without saline irrigation (Group B, n=32), and double BHD with saline irrigation (Group C, n=30). In each cases, surgical techniques were selected according to operator’s preferred method. |
| ***Efficacy Outcomes*** | Clinical data included hospital days, recurrence and Glasgow Outcome Scale (GOS) at discharge were investigated. From the GOS results, good recovery and moderate disability were defined as good outcomes, whereas, severe disability, persistent vegetative state, and death were defined as poor outcomes. Recurrence of CSDH was defined as an increase in the amount of hematoma in the surgical area, change in hematoma density, or effacement of cerebral sulci seen on CT exam within 3 months after surgery. Revision surgery was performed when neurologic symptoms was occurred. |
| ***Safety Outcomes*** | Mortality rate. |

| **Trials** | Fernandez et al. 2022 |
| --- | --- |
| ***Inclusion Criteria*** | all patients diagnosed with cSDH. |
| ***Exclusion Criteria*** | NA |
| ***Study Design*** | A retrospective single-centre study was performed from 2012 to 2018 in a third level University Hospital after collecting data from all patients diagnosed with cSDH and analysing those treated by a burr-hole craniostomy. Ethical approval was not necessary for the preparation of this article. |
| ***Efficacy Outcomes*** | Demographic data, previous medication, patient comorbidities, preoperative symptoms, clinical evolution after treatment and length of hospital stay were extracted from medical records and patients were neurologically classified preoperatively and postoperatively according to the Markwalder Grading System (MGS) (Tab. 1) [23] and the Glasgow Coma Scale (GCS). We also evaluated postoperative functional status one month after discharge according to the Glasgow Outcome Scale Extended (GOS-E). |
| ***Safety Outcomes*** | Mortality rate and complications. |

| **Trials** | Heringer et al. 2022 |
| --- | --- |
| ***Inclusion Criteria*** | Patients aged 18 years or older with a diagnosis of chronic subdural hematoma who underwent burr hole evacuation. |
| ***Exclusion Criteria*** | Patients with a history of trauma, anticoagulant or antiplatelet therapy, chronic alcohol use, or cerebrospinal fluid shunts were excluded. |
| ***Study Design*** | A retrospective review of the patients operated on because of a CSDH in a single center (Hospital do Servidor Público Estadual de São Paulo) between 2006 and 2015 was carried out. |
| ***Efficacy Outcomes*** | The presence of recurrence and complications and the postoperative functional status (Glasgow Outcome Scale - GOS) were evaluated in patients submitted to one or two burr holes and the use, or not, of a drain, and correlated with potential factors for recurrence, including: anthropometric data (age and gender), comorbidities, laterality of the hematoma (uni or bilateral), functional scales (American Society of Anesthesiologists (ASA) Score and GOS), one or two burr holes and the use, or not, of the drain. |
| ***Safety Outcomes*** | Comorbidities (surgical risk assessed by ASA), use of antiplatelet drugs or anticoagulants, alcoholism, and preoperative Glasgow Coma Scale (GCS) were recorded. The ASA surgical risk classification is a widely-used scale to classify surgical patients. A score of ASA 1 is a healthy patient with no organic changes; ASA 2 is a patient with mild or moderate systemic alteration; ASA 3 is a patient with severe systemic alteration with functional limitation; ASA 4 is a patient with severe systemic alteration representing life risk; and ASA 5 is a dying patient who is not expected to survive without surgery. |

| **Trials** | Lee et al. 2004 |
| --- | --- |
| ***Inclusion Criteria*** | CSDH was defined as a subdural hematoma surrounded by capsule (hematoma membrane) and consisting of dark reddish liquefied blood at operation. |
| ***Exclusion Criteria*** | NA |
| ***Study Design*** | Diagnosis of CSDH was confirmed by computed tomography (CT) in 157 patients and by magnetic resonance imaging (MRI) in 15 patients. Operation was performed under general anesthesia in 165 patients, and with local anesthesia in 7 patients. According to the experience and preference of the surgeon, 3 different surgical procedures were performed: two different burr-holes without membranectomy (Group A, n = 38), enlarged craniectomy with a size of about 30 mm (Group B, n = 121), and larger craniotomy (Group C, n = 13) (Tables 1 and 2). |
| ***Efficacy Outcomes*** | Patient’s clinical scores were classified comparing the preoperative and the postoperative status at the time of discharge from the hospital (Table 3), using the most common neurologic grading system for CSDH as proposed by Markwalder; reoperation. |
| ***Safety Outcomes*** | NA |

| **Trials** | Lee et al. 2009 |
| --- | --- |
| ***Inclusion Criteria*** | Eighty-seven patients have undergone surgery, and were analyzed retrospectively for CSDH at our institute from January 2004 to December 2008. |
| ***Exclusion Criteria*** | NA |
| ***Study Design*** | There were 65 males and 22 females (M:F ratio 3:1) in the study group. Median age was 65.2 with a range from 56 days old to 83 years old. We classified our patients into three groups according to the operative procedure; group I, one burr-hole craniostomy with closed system drainage with or without irrigation (n = 25), group II, two burr-hole craniostomy with closed system drainage with irrigation (n = 32), and group III, small craniotomy (about 3-4 cm in diameter) with irrigation and closed-system drainage (n = 30). |
| ***Efficacy Outcomes*** | The neurologic performance of the patients was evaluated with the “Markwalder’s Neurological Grading System” preoperatively and post-operatively which is the most commonly used neurological grading system for CSDH9). Days of post-operative hospitalization, reoperation and complication rate were also used for post-operative assessment. |
| ***Safety Outcomes*** | Complication rate |

| **Trials** | White et al. 2010 |
| --- | --- |
| ***Inclusion Criteria*** | All patients undergoing either burrhole drainage or minicraniectomy were included in the study. |
| ***Exclusion Criteria*** | Patients were excluded if the collection was secondary to an overdraining ventriculoperitoneal shunt. |
| ***Study Design*** | We reviewed retrospectively all adult patients with a primary chronic subdural haematoma treated in our Institute between the 1st of January 2003 and the 31st December 2005. The choice of surgical technique was made by the responsible consultant (any of the 8 consultants in the Institute). The decision to treat with minicraniectomy was largely made in the older patients with multiple loculations on CT scan. Of 246 patients, 130 underwent burrhole drainage and 116 patients were treated by minicraniectomy. |
| ***Efficacy Outcomes*** | The primary endpoint was asymptomatic recurrence that required intervention, either percutaneous aspiration or re-operation. |
| ***Safety Outcomes*** | Secondary endpoints included the Glasgow Outcome Score at three month follow-up, any documented improvement in preoperative conscious level or focal neurological deficit and mortality at three months. |

| **Trials** | Hussain et al. 2017 |
| --- | --- |
| ***Inclusion Criteria*** | Patients who underwent surgery for chronic subdural haematoma (CSDH) in our department over a period of 5 years (2010–2015). |
| ***Exclusion Criteria*** | NA |
| ***Study Design*** | The surgical methods included standard two burr holes or MC followed by subdural drain insertion. The patient notes including operation details, radiology reports and discharge summaries were reviewed using institutional data base. |
| ***Efficacy Outcomes*** | The data collected include: patient demographics, type of surgery, comorbidities, anticoagulation treatment, median length of hospital stay and discharge destination. |
| ***Safety Outcomes*** | Secondary endpoints included the Glasgow Outcome Score at three month follow-up, any documented improvement in preoperative conscious level or focal neurological deficit and mortality at three months. |

| **Trials** | Stavrinou et al. 2017 |
| --- | --- |
| ***Inclusion Criteria*** | Patients with 227 CSDH who were admitted to the Department of Neurosurgery, University Hospital of Cologne, Germany between January 2011 and December 2014. All patients were diagnosed using thin-sliced (3mm) computer tomography (CT). |
| ***Exclusion Criteria*** | NA |
| ***Study Design*** | We retrospectively analyzed 195 patients with 227 CSDH, Prior to intervention, anticoagulant and antiplatelet drugs were discontinued and their effects were actively reversed using vitamin K, prothrombin complex concentrates (PPSB-SD), plasma and/or platelets. Coagulation status was evaluated through controlling of the APTT and INR values as well as with the PFA-100 test. |
| ***Efficacy Outcomes*** | Prior to intervention, anticoagulant and antiplatelet drugs were discontinued and their effects were actively reversed using vitamin K, prothrombin complex concentrates (PPSB-SD), plasma and/or platelets. Coagulation status was evaluated through controlling of the APTT and INR values as well as with the PFA-100 test. |
| ***Safety Outcomes*** | NA |

| **Trials** | Raghavan et al. 2019 |
| --- | --- |
| ***Inclusion Criteria*** | All the patients who had undergone craniotomy or burr hole craniostomy for cSDHs and had been aged ≥18 years during the study period were included. |
| ***Exclusion Criteria*** | Any patient who had undergone previous cranial surgery or had presented with additional cranial pathological features, such as brain tumor or infection, were excluded. |
| ***Study Design*** | The institutional review board approved the present retrospective medical record review of patients with cSDHs who had presented to our institution from 2002 to 2015. Individual patient consent was not required, because the present study was a descriptive retrospective study, and all patient data were anonymized. A total of 299 consecutively treated patients were included in the present study. |
| ***Efficacy Outcomes*** | The patient demographic data (i.e., sex, age, alcohol use, tobacco use), medical comorbidities, and coagulation status (i.e., international normalized ratio, activated partial thromboplastin time, platelet count, anticoagulation therapy) were recorded and compared between the 2 groups. In addition, hematoma characterization, including preoperative width, location, etiology of cSDH, the presence of a midline shift and herniation on imaging studies, and presence of loculations determined from intraoperative observations, was evaluated. The Charlson comorbidity index and pre-intervention neurological status (i.e., Glasgow coma scale [GCS] and ability to independently ambulate) were also compared between 2 treatment groups. |
| ***Safety Outcomes*** | The primary outcomes included transfer to a nursing facility, the need for reoperation, and mortality within 30 days. |

| **Trials** | Haron et al. 2019 |
| --- | --- |
| ***Inclusion Criteria*** | NA |
| ***Exclusion Criteria*** | Patients <18 years or in whom a cerebrospinal fluid shunt system was present were excluded from the analysis. |
| ***Study Design*** | The records of all patients who underwent surgery for CSDH between July 2004 and July 2016 were retrospectively obtained from the digital medical records. Outcomes were assessed at a minimum of 12 months after treatment and the data were analysed. For continuous data on age, mean values, and standard deviations were calculated, and these were compared using a two-sample t-test. For categorical data on gender, mortality, and recurrence rates, proportions were calculated and compared using 95% confidence intervals of a proportion. For associations between recurrence and individual risk factors, a chi-squared test was applied. Putatively significant associations from this analysis were checked by comparing proportions and their 95% confidence intervals. For associations between recurrence and age, a Pearson correlation coefficient was calculated. |
| ***Efficacy Outcomes*** | Data were obtained on: the demographic features of the patients, the type of surgery performed, recurrence rates, and risk factors for recurrence. |
| ***Safety Outcomes*** | Mortality rates. |

| **Trials** | Shim et al. 2019 |
| --- | --- |
| ***Inclusion Criteria*** | Seventy-five patients who underwent surgery with chronic subdural hematoma in our hospital from January 2016 to December 2018. |
| ***Exclusion Criteria*** | Patients with risk factors of recurrence such as previous history of ventriculoperitoneal shunt placement, hepatic failure, renal insufficiency, or anticoagulant therapy were excluded. |
| ***Study Design*** | As retrospective chart review study, it was granted by the Institutional Review Board of Inje University Busan Paik Hospital. Sixty patients were diagnosed to chronic subdural hematoma with homogeneous and low density on computed tomography (CT) images that was considered liquefied hematoma and underwent one burr hole drainage, and 15 patients with heterogenous or high density on CT images that was considered solid or mixed form hematoma performed small craniotomy at frontal or parietal area and was done hematoma removal and saline irrigation. Drainage catheter was inserted into the subdural hematoma space in both burr hole drainage and small craniotomy group. The surgical method was determined according to the pattern of hematoma on brain CT images. The recurrence rate and length of hospitalization days were compared between the patients with burr hole drainage and small craniotomy. Statistical analysis was performed using t-test and p-value <0.05 was interpreted to have statistically significance. |
| ***Efficacy Outcomes*** | Recurrence and mean hospitalized days |
| ***Safety Outcomes*** | NA |

| **Trials** | Gazzeri et al. 2020 |
| --- | --- |
| ***Inclusion Criteria*** | Adult patients have been surgically treated due to symptomatic CSDH. |
| ***Exclusion Criteria*** | We excluded all patients who had been previously operated and those with insufficient medical records. Patients with missing data sets were excluded if the missing data were relevant to that particular analysis. Subdural hygromas, special clinical cases like calcified or ossified CSDHs (the so-called „armored brain”), asymptomatic CSDHs, and patients without sufficient follow-up data were also not considered for the present analysis. Moreover, patients with acute subdural hematomas, defined those within 3 days of injury or CT scan showing an uniformly hyperdense collection, were also excluded. |
| ***Study Design*** | We conducted a single center retrospective analysis on 414 patients surgically treated for CSDH over a period of 6 years. Comparisons were made after dividing the patients into 4 groups based on the surgical technique and type of drainage: Single burr hole with subdural drainage (Group Ia), single burr hole with subgaleal drainage (Group Ib), craniotomy with subdural drainage (Group IIa), and craniotomy with subgaleal drainage (Group IIb). 238 cases underwent burr hole with irrigation, while 290 cases were treated with craniotomy. Of the analysed patients, subdural drainage was inserted in 382 cases, while subgaleal drain was used only in 146 patients, for a total of 528 procedures. |
| ***Efficacy Outcomes*** | The primary endpoints of the study included recurrence of bleeding and re-operation rates within two months. |
| ***Safety Outcomes*** | Secondary endpoints involved the surgical complications (infection rate, post-operative acute hemorrhage and epileptic crisis) and perioperative mortality. |

| **Trials** | Vemula et al. 2020 |
| --- | --- |
| ***Inclusion Criteria*** | Patients who underwent either TC or BHD were included and divided into these two groups. |
| ***Exclusion Criteria*** | NA |
| ***Study Design*** | A retrospective analysis of all cSDH patients operated in our institute between January 2014 and August 2019 was done. The choice of either procedure was decided by individual surgeons’ preference. Patients with acute SDH, those who underwent large craniotomy or TC were excluded from the study. |
| ***Efficacy Outcomes*** | The clinical features, radiology, surgical details, recurrence rate, and complications were analyzed. Outcome was measured using Glasgow Outcome Score (GOS). |
| ***Safety Outcomes*** | Complications |

| **Trials** | Zolfaghari et al. 2021 |
| --- | --- |
| ***Inclusion Criteria*** | All patients over the age of 18 diagnosed with a surgically evacuated CSDH were eligible for the study. |
| ***Exclusion Criteria*** | The study had the following exclusion criteria: bilateral CSDHs, cerebral shunts, simultaneous intracranial hemorrhages, and patients with permanent residency outside of Sweden. |
| ***Study Design*** | The following multicenter retrospective comparative cohort study took place at Scania University Hospital and Karolinska University Hospital. The patients included from Scania University Hospital were treated between 2012 and 2016, while the patients included from Karolinska University Hospital were treated between 2006 and 2014. During the time periods, the surgical techniques and management of CSDH were not altered at either center. A total of 1003 patients were included in this retrospective cohort study. |
| ***Efficacy Outcomes*** | Radiological data was retrieved in the form of midline shift (mm) and largest hematoma diameter in the axial plane (mm). Surgical data was retrieved in the form of the type of anesthesia used (local with sedation or general anesthesia), drainage system duration, recurrence rate (defined as new evacuation of CSDH on the same side within 3 months of the initial evacuation), and mortality at 30 days and 1 year past surgical date. Reoperation was not registered as a complication in our study. |
| ***Safety Outcomes*** | Complications were registered according to Landriel Ibañez (classification system for complications after neurosurgical procedures) [15]. Complications were defined as any deviation from the normal postoperative course occurring within 30 days of surgery. |

| **Trials** | Singh et al. 2011 |
| --- | --- |
| ***Inclusion Criteria*** | 100 consecutive patients presenting to our neurosurgery department between 01-04-2010 and 31-03-2011 with CT scan or MRI showing significant hemispheric subdural hematoma (>10mm thick). |
| ***Exclusion Criteria*** | Thin SDH (<10mm) Small/ Unilobar SDH or post craniotomy SDH Infants or cases of birth related subdural collections Radiological doubt of hygroma/ empyema Bilateral significant lesions (for ease of analysis) Large acute clot component (organized hyperdensity)* |
| ***Study Design*** | This study was designed as a randomized prospective trial. Since complete blinding is impossible in any surgical trial, we undertook to remove bias as far as possible by third party blinding in as such that the person filling up the discharge and one-month follow up forms was blinded to the type of intervention done. |
| ***Efficacy Outcomes*** | The primary outcome variable studied was clinically significant recurrence rate, which effectively translates to need for re-aspiration/ re-do surgery. |
| ***Safety Outcomes*** | Secondary outcome variables studied were – 1. GCS and Markwalder grade at discharge 2. GOS and Markwalder grade at 1 month follow-up 3. Time of surgery- prepping up to dressing 4. Radiological assessment at admission, discharge and at follow-up – regarding hematoma thickness and midline shift.  5. Complications–respiratory, cardiac, wound infection, subdural hematoma formation, cortical damage |

| **Trials** | Gernsback et al. 2016 |
| --- | --- |
| ***Inclusion Criteria*** | Patients undergoing burr hole drainage for 261 cSDH in terms of preoperative comorbidities and post-operative drain placement. |
| ***Exclusion Criteria*** | NA |
| ***Study Design*** | We retrospectively analyzed all burr hole drainages performed by two trauma surgeons from 2007 to 2015. Surgery was performed using a 14mm perforator drill to make one or two burr holes, at the surgeon’s discretion. Typically, one burr hole was used for smaller cSDH, and two were used for larger, holohemispheric cSDH. A surgery was included if the post-operative diagnosis was chronic subdural hematoma or hygroma, and either no drain was left, or one or more external ventricular drain-type catheters were left in place. The number of drains placed was at the operating surgeon’s discretion. |
| ***Efficacy Outcomes*** | Data was collected on demographics, hematoma laterality, patient comorbidities, pre-operative medications, number of burr holes, number of drains left and recurrences. |
| ***Safety Outcomes*** | NA |

| **Trials** | Kansal et al. 2010 |
| --- | --- |
| ***Inclusion Criteria*** | A total of 267 patients with CSDH, treated at a tertiary care hospital in a neurosurgical unit during the years 2005 to 2008, were included in this study. Patients with bilateral subdural hematomas were excluded. |
| ***Exclusion Criteria*** | Those patients who had the following medical conditions were also not included: liver failure, coagulopathy, those with a ventriculoperitoneal shunt, seizure history and significant brain atrophy. |
|  |  |
| ***Study Design*** | Of these patients, 187 underwent surgery under local anesthesia and 80 were evacuated under general anesthesia. All operations were principally performed by senior residents of the neurosurgical service. In the earlier phase of the study (January 2005–March 2006), double burr holes were performed and in the second phase (April 2006–December 2008), a single burr hole was performed. The patients undergoing double burr hole drainage were labeled as Group 1 and those with a single burr hole were designated as Group 2. |
| ***Efficacy Outcomes*** | Recurrence and reoperation |
| ***Safety Outcomes*** | NA |

| **Trials** | Han et al. 2009 |
| --- | --- |
| ***Inclusion Criteria*** | From January 2002 to December 2006, 180 consecutive patients (129 men and 51 women) with CSDH underwent burr hole craniostomy with closed-system drainage at our hospital were retrospectively enrolled in this study. |
| ***Exclusion Criteria*** | Two patients at the age of 2 months and 1 year were excluded from the study. Three additional patients treated with craniotomy were excluded. |
|  |  |
| ***Study Design*** | CSDH was defined as the presence of a typical neomembrane, liquefied blood within the hematoma cavity, and at least 3 weeks had passed from the event of head trauma.Diagnosis was confirmed by computed tomography (CT) scans and/or magnetic resonance (MR) images in all patients. Operations were performed under either general or local anesthesia. All patients underwent surgical procedures of one or two burr holes over the area of maximal hematoma width and closed-system drainage with the commercial silicone catheter and bag. The selection of the number of burr hole was made by the attending neurosurgeon’s preference. Subdural hematoma was spontaneously evacuated after burr hole trephination and the drainage catheter was inserted. |
| ***Efficacy Outcomes*** | Clinical and/or radiological criteria were used for evaluation of CSDH recurrence. Also, both the clinical and/or radiological criteria were used for determining reoperation. The clinical criteria for recurrence included a change in mental status, worsening of the preexisting neurological deficit and new onset or aggravation of headache. And, the radiological criteria were the CT scans during the follow-up period postsurgically revealed an increased subdural collection and/or the brain parenchyma was compressed compared to the findings of the immediate postoperative CT scans. That is, patients underwent reoperation if neurological symptoms recurred, and/or if the patients developed new neurological symptoms, with the increase in hematoma thickness and volume on follow-up CT scans as defined above. |
| ***Safety Outcomes*** | NA |

| **Trials** | Taussky et al. 2008 |
| --- | --- |
| ***Inclusion Criteria*** | Patients with 96 chronic subdural haematomas who underwent burr hole craniostomy between 1 January 2004 and 31 December 2005, in the Department of Neurosurgery, Kantonsspital Aarau, Switzerland. |
| ***Exclusion Criteria*** | NA |
|  |  |
| ***Study Design*** | This retrospective study included 76 consecutive patients with 96 chronic subdural haematomas who underwent burr hole craniostomy between 1 January 2004 and 31 December 2005, in the Department of Neurosurgery, Kantonsspital Aarau, Switzerland. |
| ***Efficacy Outcomes*** | Total no. of operative recurrences; hospitalization length; glasgow Coma scale; markwalder score; |
| ***Safety Outcomes*** | Mortality; complications; wound infection |

| **Trials** | Thavara et al. 2019 |
| --- | --- |
| ***Inclusion Criteria*** | Patients between 18 and 90 years of age were selected. Patients with CSDH showing computed tomography (CT) scan findings of homogeneous hypodensity, homogeneous isodensity, and mixed density were selected. CT scan findings of CSDH with hyperdense gravity‑dependent fluid level were also selected. |
| ***Exclusion Criteria*** | Patients with CT evidence of multiple septations were excluded from the study. Recurrent CSDH, bilateral CSDH, and CSDH with secondary acute bleed were also excluded. |
|  |  |
| ***Study Design*** | The authors retrospectively compared the data of the patients of CSDH who had undergone Single BHC or TDC between January 2014 and December 2016. The study was conducted in the Department of Neurosurgery, Government Medical College, Thrissur, Kerala, India, and approved by the Institutional Review Board of the Institution. |
| ***Efficacy Outcomes*** | The data collected includes age, sex, duration of symptoms in days, history of trauma of any severity, clinical features, type of the procedure performed, prothrombin time (PT) in seconds, INR, activated partial thromboplastin time (APTT) in seconds, platelet counts per microliter of blood, use of antiplatelets or anticoagulants, comorbidities, history of chronic alcoholism, and duration of procedure and postoperative neurological status. Pre‑ and post‑operative eye opening and verbal and motor scores of GCS were separately noted. But for statistical calculation, total GCS score were used. Pre‑ and post‑operative motor power of the affected limbs was measured in the Medical Research Council grading. The thickness, location and side of CSDH, and presence of MLS in preoperative CT scan were noted. Improvement in postoperative CT scan and any occurrence of complications were noted. The presence of significant residual CSDH requiring reoperation was noted. Reoperation due to complication of surgery was noted. Duration of the hospital stay was also noted. |
| ***Safety Outcomes*** | Mortality; complications. |

| **Trials** | Kim et al. 2019 |
| --- | --- |
| ***Inclusion Criteria*** | Patients between 18 and 90 years of age were selected. Patients with CSDH showing computed tomography (CT) scan findings of homogeneous hypodensity, homogeneous isodensity, and mixed density were selected. CT scan findings of CSDH with hyperdense gravity‑dependent fluid level were also selected. |
| ***Exclusion Criteria*** | Patients with CT evidence of multiple septations were excluded from the study. Recurrent CSDH, bilateral CSDH, and CSDH with secondary acute bleed were also excluded. |
|  |  |
| ***Study Design*** | The authors retrospectively compared the data of the patients of CSDH who had undergone Single BHC or TDC between January 2014 and December 2016. The study was conducted in the Department of Neurosurgery, Government Medical College, Thrissur, Kerala, India, and approved by the Institutional Review Board of the Institution. |
| ***Efficacy Outcomes*** | The data collected includes age, sex, duration of symptoms in days, history of trauma of any severity, clinical features, type of the procedure performed, prothrombin time (PT) in seconds, INR, activated partial thromboplastin time (APTT) in seconds, platelet counts per microliter of blood, use of antiplatelets or anticoagulants, comorbidities, history of chronic alcoholism, and duration of procedure and postoperative neurological status. Pre‑ and post‑operative eye opening and verbal and motor scores of GCS were separately noted. But for statistical calculation, total GCS score were used. Pre‑ and post‑operative motor power of the affected limbs was measured in the Medical Research Council grading. The thickness, location and side of CSDH, and presence of MLS in preoperative CT scan were noted. Improvement in postoperative CT scan and any occurrence of complications were noted. The presence of significant residual CSDH requiring reoperation was noted. Reoperation due to complication of surgery was noted. Duration of the hospital stay was also noted. |
| ***Safety Outcomes*** | Mortality; complications. |

| **Trials** | Smely et al. 1997 |
| --- | --- |
| ***Inclusion Criteria*** | From January to September 1996, 33 consecutive patients were admitted for neurosurgical treatment of 36 CSDH. |
| ***Exclusion Criteria*** | NA |
|  |  |
| ***Study Design*** | The data were compared with a group of 33 consecutive patients treated by the BHC from January to September 1993. |
| ***Efficacy Outcomes*** | Anamnestic data and neurological performance were evaluated according to Markwalder's Grading Scale (MGS) on admission, on first postop, day, at first discharge, and similarly, during following in-patient stays and later out-patient controls. |
| ***Safety Outcomes*** | The number of surgical interventions following these procedures due to persistence or relapse of haematoma were evaluated as well as morbidity and mortality aspects in both patient groups. |

| **Trials** | Williams et al. 2000 |
| --- | --- |
| ***Inclusion Criteria*** | Patients underwent surgical evaluation for chronic subdural haematomata, which is defined as a collection of fluid within the potential subdural space radiographically consistent with liquefying blood appearing hypodense by CT scan evaluation. |
| ***Exclusion Criteria*** | NA |
|  |  |
| ***Study Design*** | The results from sixty-two patients diagnosed with chronic subdural haematoma were evaluated for technique, postoperative computerized tomography (CT) scan results, and complications. |
| ***Efficacy Outcomes*** | Recurrence and reoperation. |
| ***Safety Outcomes*** | Complication. |

| **Trials** | Lin et al. 2011 |
| --- | --- |
| ***Inclusion Criteria*** | Patients with CSDH received surgical treatment at the Department of Neurosurgery, Tianjin Huanhu Hospital & Tianjin Neurosurgical Research Institute in China. The CSDH was characterized as a subdural hematoma surrounded by capsule (hematoma membrane) consisting of dark reddish liquefied blood. |
| ***Exclusion Criteria*** | NA |
|  |  |
| ***Study Design*** | A retrospective study of 448 patients with CSDH by surgical treatment during 2005 to 2009 was conducted in order to compare the efficiency between two different primarysurgical methods, twist-drill drainage without irrigation in Group A (n=178) and one burr-hole with irrigation in Group B (n=270). The results were statistically analyzed. |
| ***Efficacy Outcomes*** | Recurrence and reoperation. |
| ***Safety Outcomes*** | Complication. |

| **Trials** | Certo et al. 2019 |
| --- | --- |
| ***Inclusion Criteria*** | Fifteen patients (9 males) suffering from CSDH. |
| ***Exclusion Criteria*** | NA |
|  |  |
| ***Study Design*** | We retrospectively analyse the clinical and radiological data of a minimally invasive, percutaneous draining system (Integra ™) used in fifteen patients (Group A; mean age: 75.7) with CSH, and compare them with those obtained from two retrospective series of patients: the first one (Group B 15 patients, mean age 77.1) treated with standard, single-burr hole technique for subdural drainage under general anaesthesia; the second one (Group C 15 patients, mean age 76.4) treated with standard, single-burr hole technique for subdural drainage under local anaesthesia and mild sedation. |
| ***Efficacy Outcomes*** | We evaluated the impact of pre-existing neurological conditions on clinical outcome pre- and post-operatively (one month after surgery) for all patients in three groups using the Unified Parkinson’s Disease Rating Scale (UPDRS) part III and Mini Mental State Exam (MMSE). We compared length of post-operative hospitalization and use of analgesics between the three groups. We also evaluated the time of drain maintenance as well as the incidence of complications. Radiological evaluation was performed comparing the maximal thickness of the subdural collection detected on pre-operative and post-operative CT scans obtained at 48 h and 21 days after the operation, respectively. |
| ***Safety Outcomes*** | Complication. |

| **Trials** | Garber et al. 2016 |
| --- | --- |
| ***Inclusion Criteria*** | we performed a dual-center retrospective chart review of two cohorts of patients treated between January 1, 2007, and December 31, 2012. The indication for all surgical procedures was the presence of a symptomatic subacute or CSDH. |
| ***Exclusion Criteria*** | Patients < 18 years or with acute SDHs were excluded from this study. |
|  |  |
| ***Study Design*** | We retrospectively reviewed clinical and radiographic features in patients who underwent bedside twist drill evacuation of a CSDH and those for a cohort of patients who underwent operative intervention via burr holes. |
| ***Efficacy Outcomes*** | The clinical features evaluated were age, sex, mechanism of injury, associated comorbidities, seizure, anticoagulation or antiplatelet therapy, admission international normalized ratio (INR), and length of hospital stay. |
| ***Safety Outcomes*** | Complication. |

| **Trials** | Lee et al. 2016 |
| --- | --- |
| ***Inclusion Criteria*** | The symptomatic CSDHs of 86 patients who had follow-up periods of more than 3 months were included in this study. |
| ***Exclusion Criteria*** | NA |
|  |  |
| ***Study Design*** | We analyzed symptomatic CSDHs in whom TDC at the pre-coronal suture entry point (PCSEP) was the primary method for hematoma drainage and BHC on the parietal was the secondary option. |
| ***Efficacy Outcomes*** | Markwalder’s grade, days of hospital stay |
| ***Safety Outcomes*** | Complication. |

| **Trials** | Wang K et al. 2017 |
| --- | --- |
| ***Inclusion Criteria*** | (a) patient age ≥ 18 years old; (b) initial unilateral CSDH without loculation confirmed by computed tomography (CT); (c) patients who received either TDC or BHC treatment; (d) patients with written consent from their next of kin. |
| ***Exclusion Criteria*** | Patients with recurrent or bilateral CSDH were excluded from this study. Patients with severe systemic diseases, such as chronic heart failure, coagulation disorders, thrombocytopenia, respiratory insufficiency, liver or renal dysfunction, were excluded. Patients dependent on anticoagulants or alcohol were also excluded. |
|  |  |
| ***Study Design*** | A prospective cohort study was conducted in the patients who suffered from symptomatic CSDH and received surgical treatment in our department from Jan 2011 to Dec 2013. Each patient was followed 3 months after the surgery. |
| ***Efficacy Outcomes*** | Markwalder’s grade, days of hospital stay |
| ***Safety Outcomes*** | Complication. |

| **Trials** | Wang Q et al. 2016 |
| --- | --- |
| ***Inclusion Criteria*** | Patients undergoing surgery for CSDH from September 2009 to November 2014 at the Department of Neurosurgery of the Second Clinical School of Yangzhou University, Jiangsu, China. |
| ***Exclusion Criteria*** | NA |
|  |  |
| ***Study Design*** | A retrospective study involving 121 patients with CSDH who underwent surgery at a single center was conducted, involving 68 patients undergoing modified TDC (TDC group) and 53 patients treated by BHC (BHC group). |
| ***Efficacy Outcomes*** | These evaluations assessed neurological symptoms (headache, weakness, cognitive decline, and consciousness) and radiological features (hematoma size and extent of midline shift). The largest width of the hematoma was considered to be the size of the hematoma and was obtained via CT or magnetic resonance imaging (MRI). Patients showing the reappearance of a hyperdense crescent-shaped hematoma or increasing hematoma cavity volume on the operated side on a CT scan within a few months after surgery were considered to have experienced recurrence; symptomatic hematomas with a significant mass effect underwent repeated surgeries. |
| ***Safety Outcomes*** | Complication. |

| **Trials** | Katsigiannis et al. 2016 |
| --- | --- |
| ***Inclusion Criteria*** | Patients with CSDH treated by drainage in the Department of Neurosurgery, University Hospital of Cologne, Germany between January 2011 and December 2014. |
| ***Exclusion Criteria*** | Patients treated outside our study period, or patients with recurrent CSDH or who had treatment other than single or two burr-hole craniostomy (BHC) or a mini craniotomy as well as those that were lost to follow up were excluded. |
|  |  |
| ***Study Design*** | We retrospectively reviewed 197 consecutive surgical cases of CSDH. Univariate and multivariate analyses were performed to identify the relationship between clinical plus radiographic factors and outcome. Imaging analysis was performed using computer-assisted 3Dvolumetric analysis. |
| ***Efficacy Outcomes*** | Duration from head trauma to operation, various comorbidities, anticoagulant and/or antiplatelet treatment, alcohol abuse as defined in the international classification of diseases (ICD-10), ventriculoperitoneal shunt in situ and preoperative neurological condition. The latter was evaluated using the classification of clinical status for patients with CSDH, known as the Markwalder Grading Scale (MGS). |
| ***Safety Outcomes*** | Routine clinical examination was carried out at 1 and 3 months after surgery, or nonregularly, upon the occurrence of new symptoms. The functional outcome at 3 months was assessed using the GOS.3In this study the dichotomized version comprising of favorable (grades 5 and 4) and unfavorable outcome (grades 3, 2 and 1) was utilized. |

**Table S4: Detailed certainty of evidence for each outcome in league table.**

| Comparison | Number of studies | Within-study bias | Reporting bias | Indirectness | Imprecision | Heterogeneity | Incoherence | Confidence rating | Reason(s) for downgrading |
| --- | --- | --- | --- | --- | --- | --- | --- | --- | --- |
| **1. Recurrence** | | | | | | | | | |
| SBHC:DBHC | 12 | No concerns | Low risk | No concerns | Some concerns | No concerns | No concerns | Moderate | ["Imprecision"] |
| SBHC:MC | 7 | Some concerns | Low risk | No concerns | Some concerns | No concerns | No concerns | Moderate | ["Within-study bias","Imprecision"] |
| SBHC:TDC | 11 | No concerns | Low risk | No concerns | Some concerns | No concerns | No concerns | Moderate | ["Imprecision"] |
| DBHC:MC | 4 | No concerns | Low risk | No concerns | No concerns | Some concerns | No concerns | Moderate | ["Heterogeneity"] |
| DBHC:TDC | 4 | No concerns | Low risk | No concerns | Major concerns | No concerns | No concerns | Low | ["Imprecision"] |
| MC:TDC | 1 | No concerns | Low risk | No concerns | Major concerns | No concerns | No concerns | Low | ["Imprecision"] |
| **2. Reoperation** | | | | | | | | | |
| SBHC:DBHC | 4 | No concerns | Low risk | No concerns | No concerns | Some concerns | No concerns | Moderate | ["Heterogeneity"] |
| SBHC:CRAN | 1 | Some concerns | Low risk | No concerns | Some concerns | No concerns | No concerns | Low | ["Within-study bias","Imprecision"] |
| SBHC:MC | 4 | Some concerns | Low risk | No concerns | Some concerns | No concerns | No concerns | Low | ["Within-study bias","Imprecision"] |
| SBHC:TDC | 10 | No concerns | Low risk | No concerns | Some concerns | No concerns | No concerns | Moderate | ["Imprecision"] |
| DBHC:CRAN | 1 | Some concerns | Low risk | No concerns | Major concerns | No concerns | No concerns | Very low | ["Within-study bias","Imprecision"] |
| DBHC:MC | 3 | No concerns | Low risk | No concerns | Some concerns | No concerns | No concerns | Moderate | ["Imprecision"] |
| DBHC:TDC | 3 | No concerns | Low risk | No concerns | No concerns | Some concerns | No concerns | Moderate | ["Heterogeneity"] |
| CRAN:MC | 1 | Some concerns | Low risk | No concerns | Major concerns | No concerns | No concerns | Very low | ["Within-study bias","Imprecision"] |
| MC:TDC | 1 | No concerns | Low risk | No concerns | Major concerns | No concerns | No concerns | Low | ["Imprecision"] |
| CRAN:TDC | 0 | Some concerns | Low risk | No concerns | Some concerns | No concerns | No concerns | Moderate | ["Within-study bias","Imprecision"] |
| **3. Favorable outcome** | | | | | | | | | |
| SBHC:DBHC | 3 | Some concerns | Low risk | No concerns | Some concerns | No concerns | No concerns | Low | ["Within-study bias","Imprecision"] |
| SBHC:MC | 2 | Some concerns | Low risk | No concerns | Some concerns | No concerns | No concerns | Low | ["Within-study bias","Imprecision"] |
| SBHC:TDC | 8 | No concerns | Low risk | No concerns | Some concerns | No concerns | No concerns | Moderate | ["Imprecision"] |
| DBHC:MC | 3 | No concerns | Low risk | No concerns | Some concerns | No concerns | No concerns | Moderate | ["Imprecision"] |
| DBHC:TDC | 3 | No concerns | Low risk | No concerns | Some concerns | No concerns | No concerns | Moderate | ["Imprecision"] |
| MC:TDC | 1 | No concerns | Low risk | No concerns | Some concerns | No concerns | No concerns | Moderate | ["Imprecision"] |
| **4. Length of hospital stay** | | | | | | | | | |
| SBHC:DBHC | 3 | No concerns | Low risk | No concerns | Some concerns | No concerns | Some concerns | Low | ["Imprecision","Incoherence"] |
| SBHC:CRAN | 1 | Some concerns | Low risk | No concerns | Major concerns | No concerns | Major concerns | Very low | ["Within-study bias","Imprecision","Incoherence"] |
| SBHC:MC | 2 | No concerns | Low risk | No concerns | Some concerns | No concerns | No concerns | Moderate | ["Imprecision"] |
| SBHC:TDC | 4 | No concerns | Low risk | No concerns | No concerns | No concerns | No concerns | High | [] |
| DBHC:MC | 1 | No concerns | Low risk | No concerns | Some concerns | No concerns | No concerns | Moderate | ["Imprecision"] |
| DBHC:CRAN | 0 | No concerns | Low risk | No concerns | Major concerns | No concerns | Major concerns | Very low | ["Imprecision","Incoherence"] |
| DBHC:TDC | 0 | No concerns | Low risk | No concerns | Some concerns | No concerns | Some concerns | Low | ["Imprecision","Incoherence"] |
| CRAN:MC | 0 | Some concerns | Low risk | No concerns | Some concerns | No concerns | Major concerns | Very low | ["Within-study bias","Imprecision","Incoherence"] |
| CRAN:TDC | 0 | Some concerns | Low risk | No concerns | Major concerns | No concerns | No concerns | Very low | ["Within-study bias","Imprecision"] |
| MC:TDC | 0 | Some concerns | Low risk | No concerns | Major concerns | No concerns | Some concerns | Very low | ["Within-study bias","Imprecision","Incoherence"] |
| **5. Operation time** | | | | | | | | | |
| SBHC:DBHC | 3 | No concerns | Low risk | No concerns | No concerns | Major concerns | Major concerns | Very low | ["Heterogeneity","Incoherence"] |
| SBHC:MC | 1 | Some concerns | Low risk | No concerns | No concerns | No concerns | No concerns | Moderate | ["Within-study bias"] |
| SBHC:TDC | 4 | Some concerns | Low risk | No concerns | No concerns | No concerns | Major concerns | Very low | ["Within-study bias","Incoherence"] |
| DBHC:MC | 2 | No concerns | Low risk | No concerns | Major concerns | No concerns | No concerns | Low | ["Imprecision"] |
| DBHC:TDC | 1 | No concerns | Low risk | No concerns | No concerns | No concerns | No concerns | High | [] |
| MC:TDC | 1 | No concerns | Low risk | No concerns | No concerns | No concerns | No concerns | High | [] |
| **6. Complication** | | | | | | | | | |
| SBHC:DBHC | 6 | No concerns | Low risk | No concerns | Some concerns | No concerns | No concerns | Moderate | ["Imprecision"] |
| SBHC:MC | 4 | Some concerns | Low risk | No concerns | Some concerns | No concerns | No concerns | Low | ["Within-study bias","Imprecision"] |
| SBHC:TDC | 8 | No concerns | Low risk | No concerns | Some concerns | No concerns | No concerns | Moderate | ["Imprecision"] |
| DBHC:MC | 2 | No concerns | Low risk | No concerns | Major concerns | No concerns | No concerns | Low | ["Imprecision"] |
| DBHC:TDC | 4 | No concerns | Low risk | No concerns | Major concerns | No concerns | No concerns | Low | ["Imprecision"] |
| MC:TDC | 1 | No concerns | Low risk | No concerns | Some concerns | No concerns | No concerns | Moderate | ["Imprecision"] |
| **7. Mortality** | | | | | | | | | |
| SBHC:DBHC | 5 | No concerns | Low risk | No concerns | Some concerns | No concerns | No concerns | Moderate | ["Imprecision"] |
| SBHC:MC | 4 | Major concerns | Low risk | No concerns | Some concerns | Some concerns | No concerns | Very low | ["Within-study bias","Imprecision","Heterogeneity"] |
| SBHC:TDC | 7 | No concerns | Low risk | No concerns | Some concerns | No concerns | No concerns | Moderate | ["Imprecision"] |
| DBHC:MC | 3 | No concerns | Low risk | No concerns | Major concerns | No concerns | No concerns | Low | ["Imprecision"] |
| DBHC:TDC | 3 | No concerns | Low risk | No concerns | Some concerns | No concerns | No concerns | Moderate | ["Imprecision"] |
| MC:TDC | 1 | Some concerns | Low risk | No concerns | Some concerns | No concerns | No concerns | Low | ["Within-study bias","Imprecision"] |

Table S5: Heterogeneity of the network meta-analysis

| Outcomes | Heterogeneity (%) |
| --- | --- |
| Recurrence | 53.4 |
| Reoperation | 52.1 |
| Favorable outcome | 54.9 |
| Length of hospital stay | 69.8 |
| Operation time | 0 |
| Complication | 91 |
| Mortality | 99.5 |

**Figure S1: Risk of bias assessment of RCT with ROB**


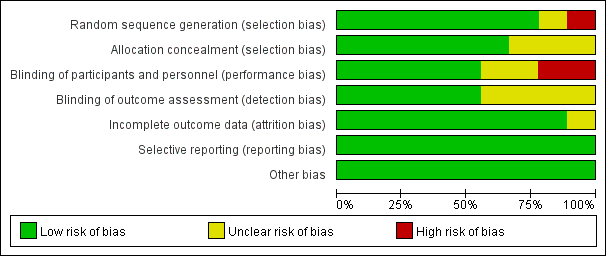


**Figure S2: Summary of risk of bias assessment of RCT with ROB.**


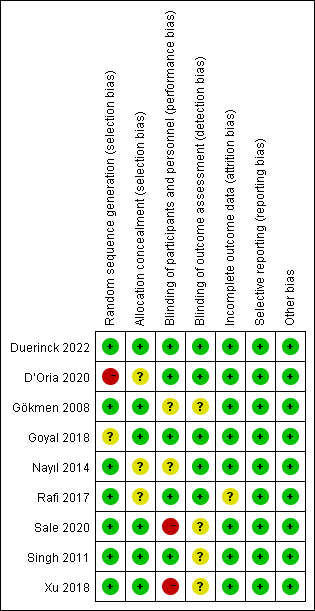


**Figure S3: Risk of bias assessment of retrospective studies with ROBINS-I.**


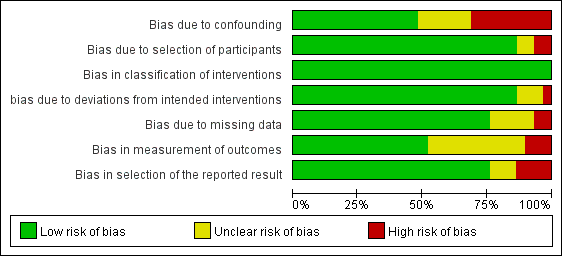


**Figure S4: Summary of risk of bias assessment of retrospective studies with ROBINS-I.**


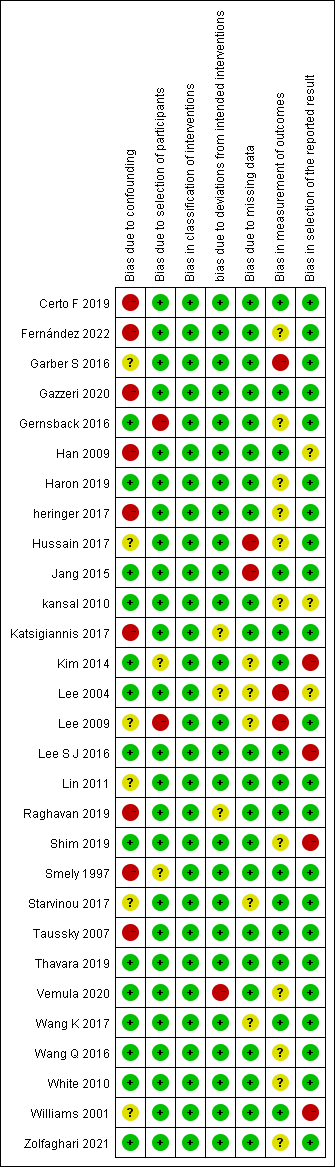


**Figure S5: Forest plots for the inconsistency: Recurrence.**


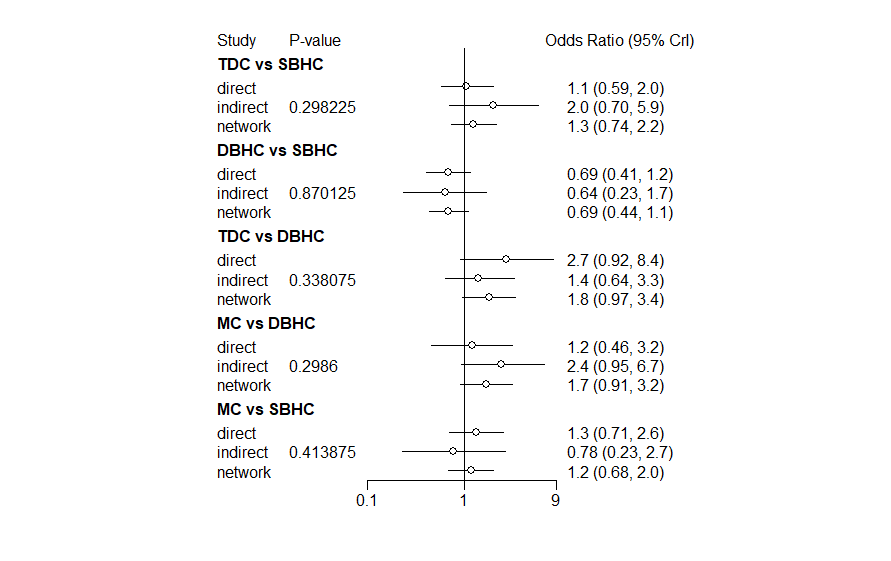


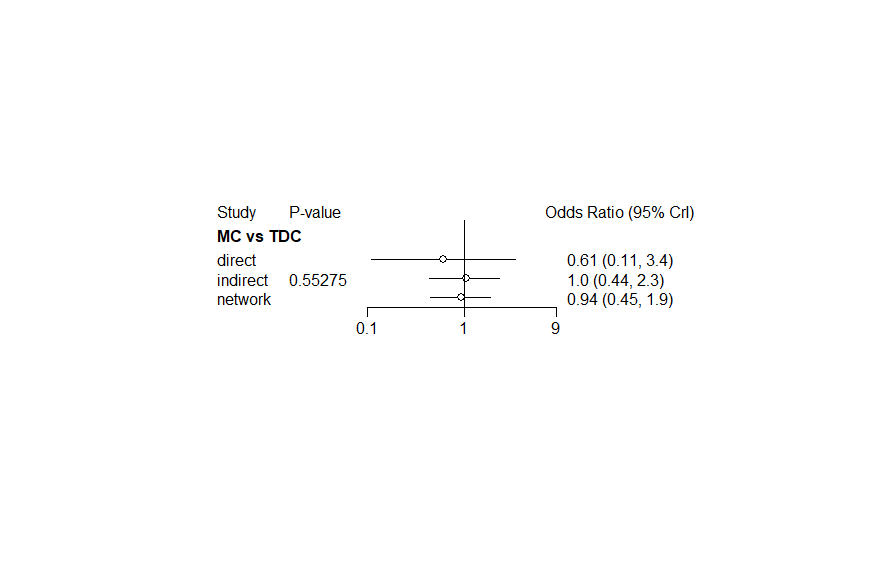


**Figure S6: Forest plots for the inconsistency: Reoperation.**


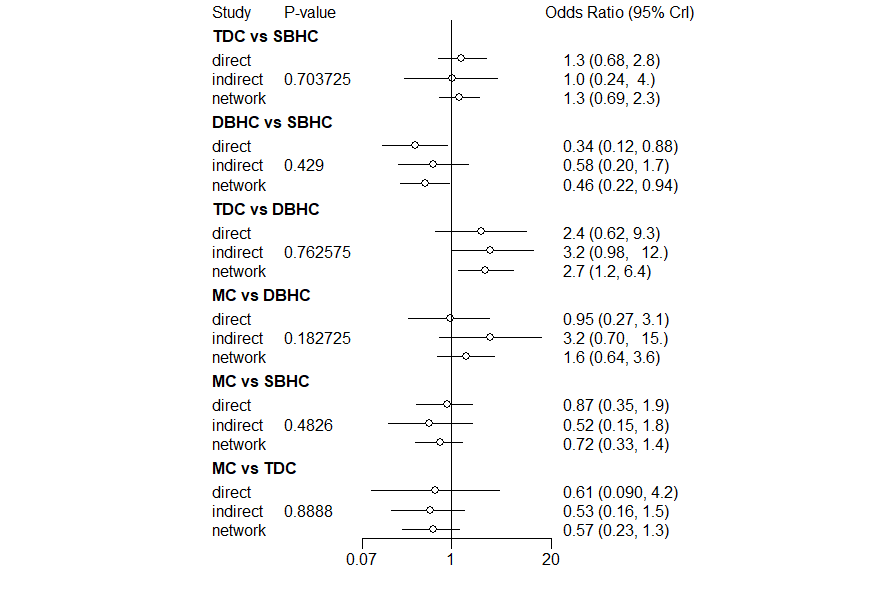

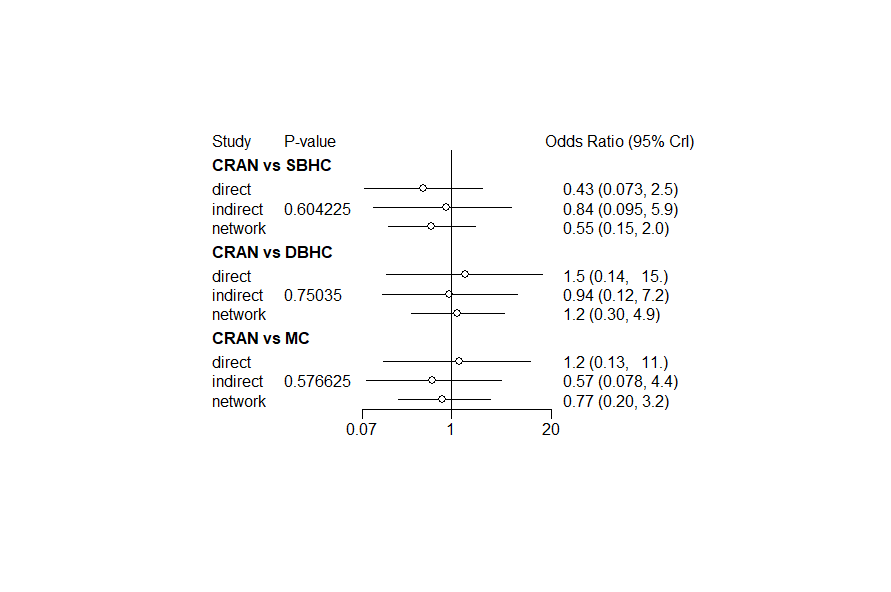


**Figure S7: Forest plots for the inconsistency: Favorable outcome.**


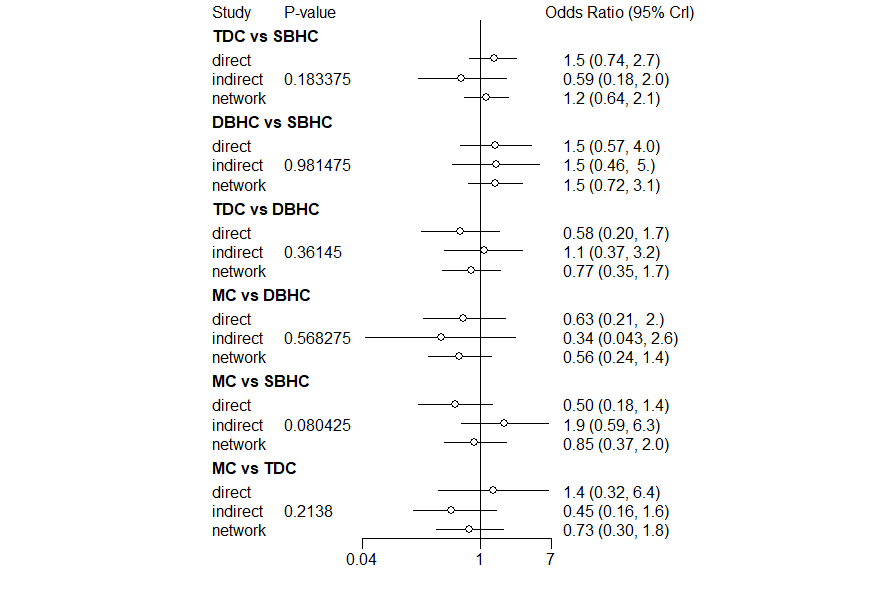


**Figure S8: Forest plots for the inconsistency: LOS.**


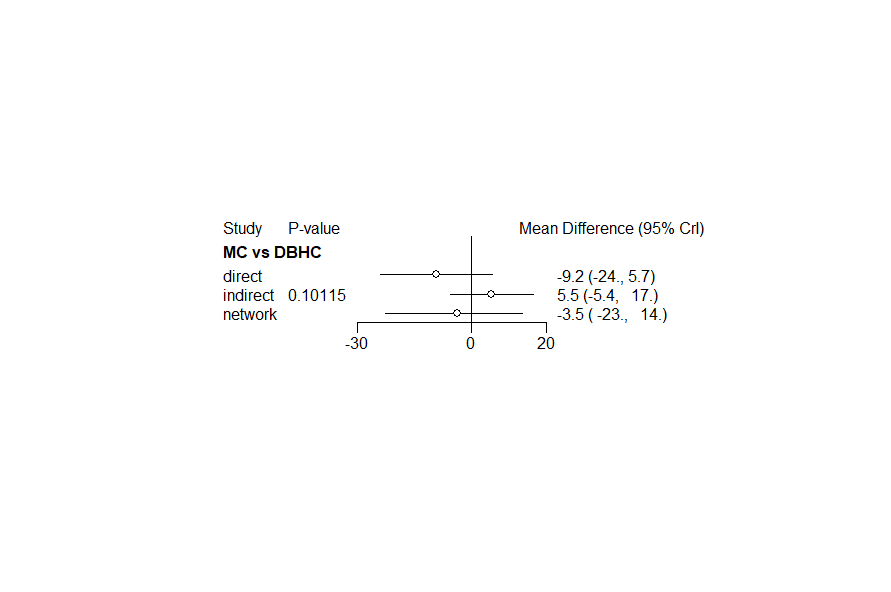


**Figure S9: Forest plots for the inconsistency: Operation time.**


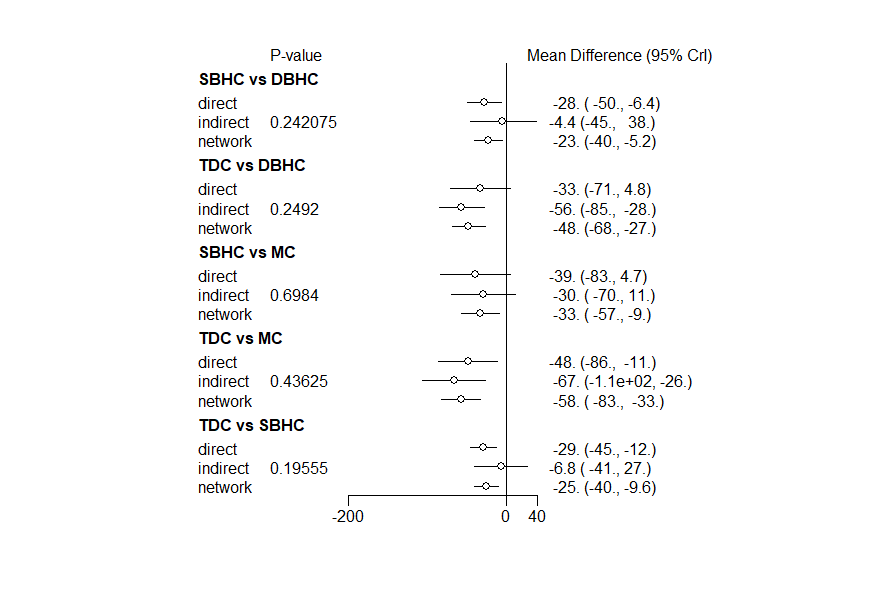
**Figure S10: Forest plots for the inconsistency: Complication.**


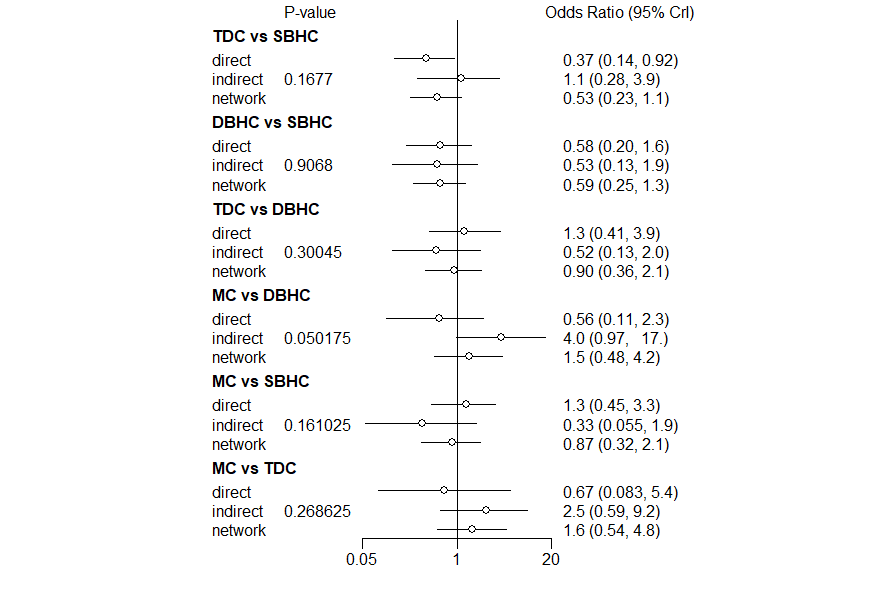


**Figure S11: Forest plots for the inconsistency: Mortality.**


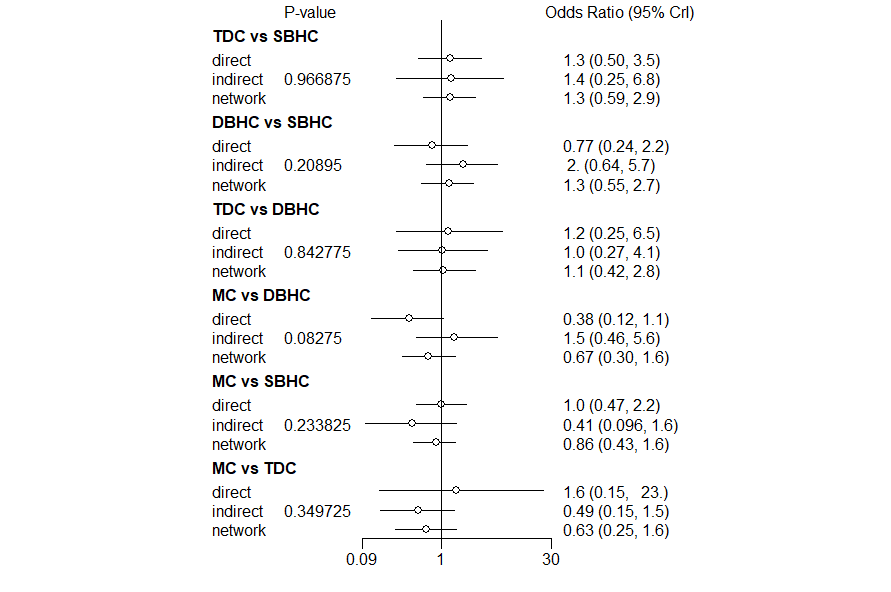


**Figure S12: Funnel plot for Recurrence.**


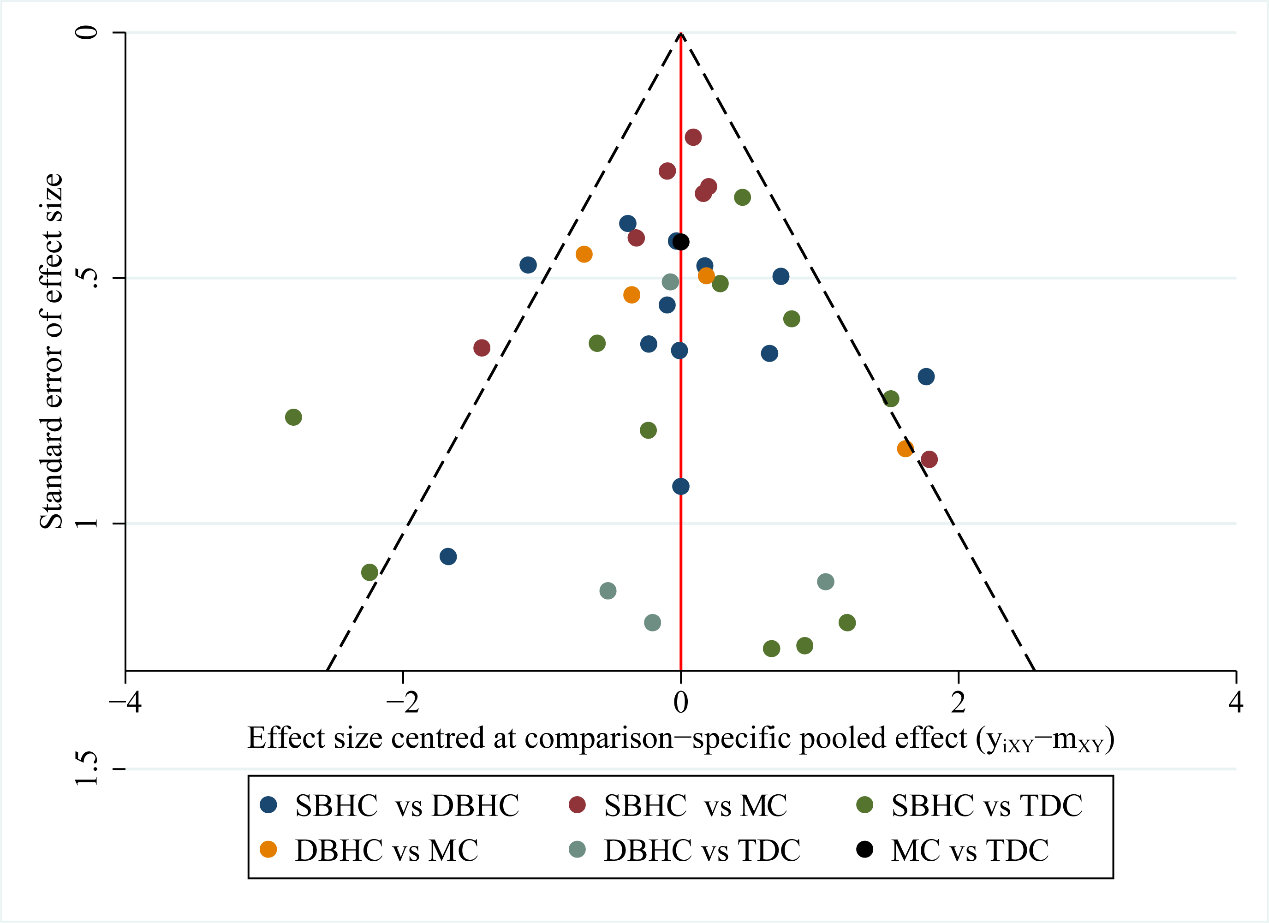


**Figure S13: Funnel plot for Reoperation.**


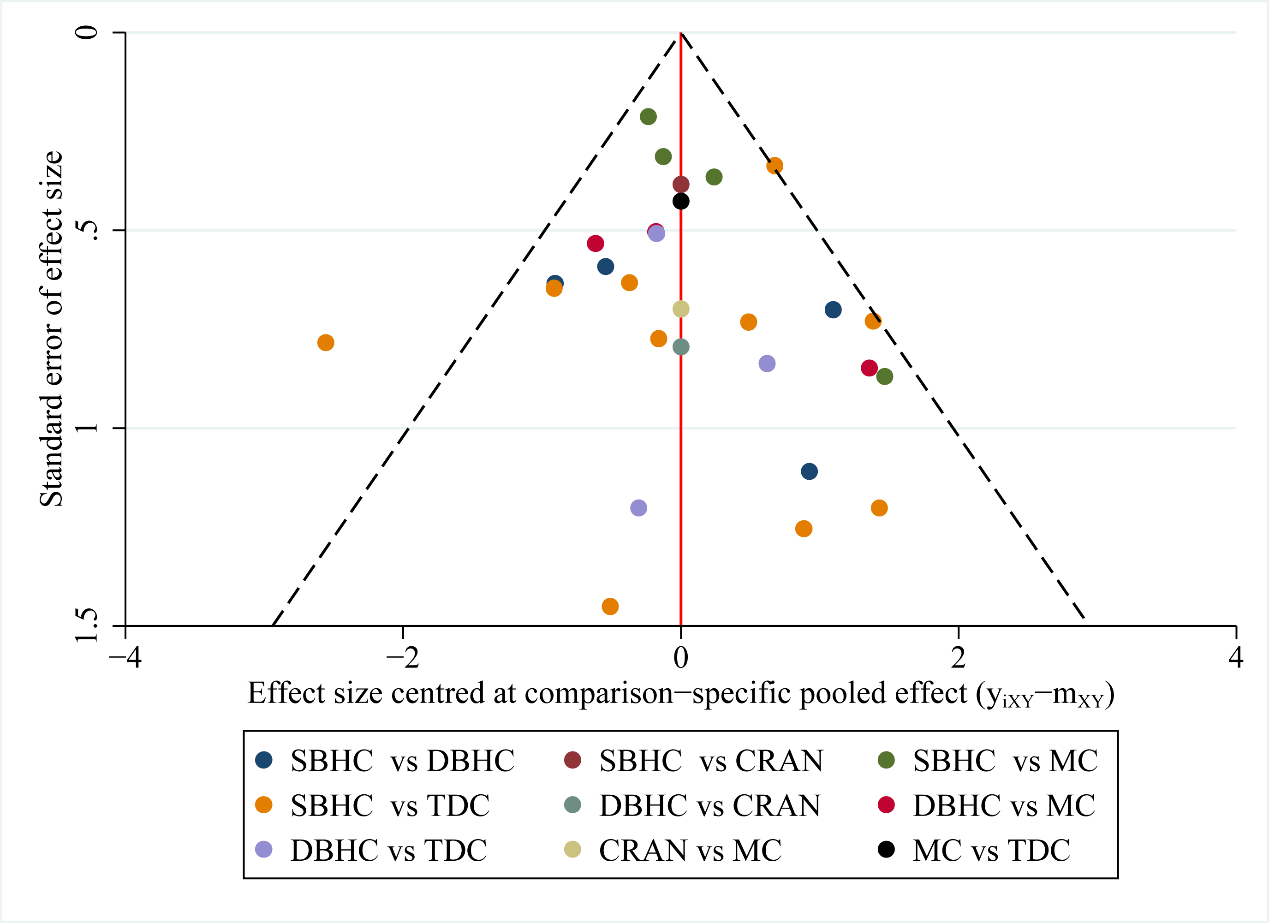


**Figure S14: Funnel plot for Favorable outcome.**


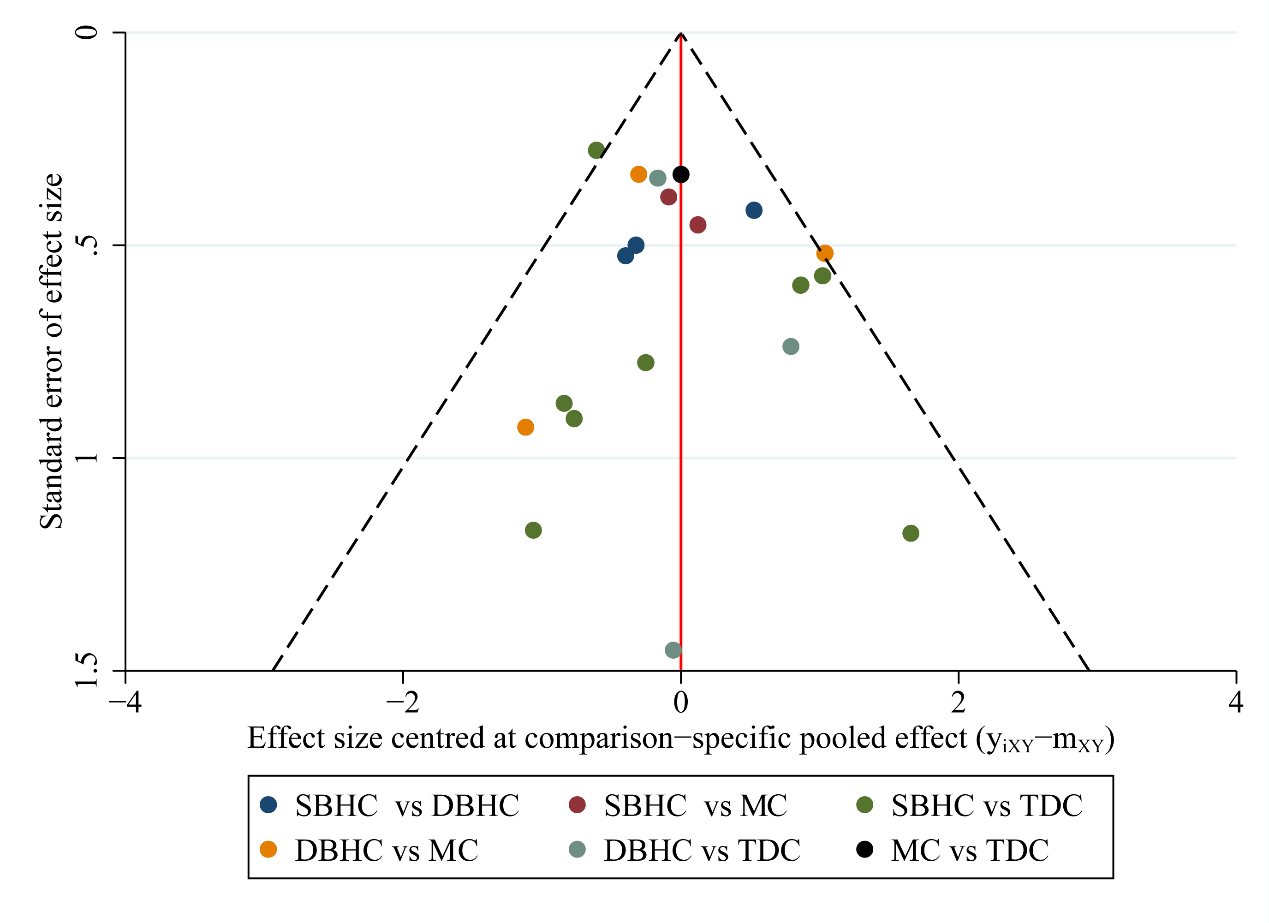


**Figure S15: Funnel plot for LOS.**


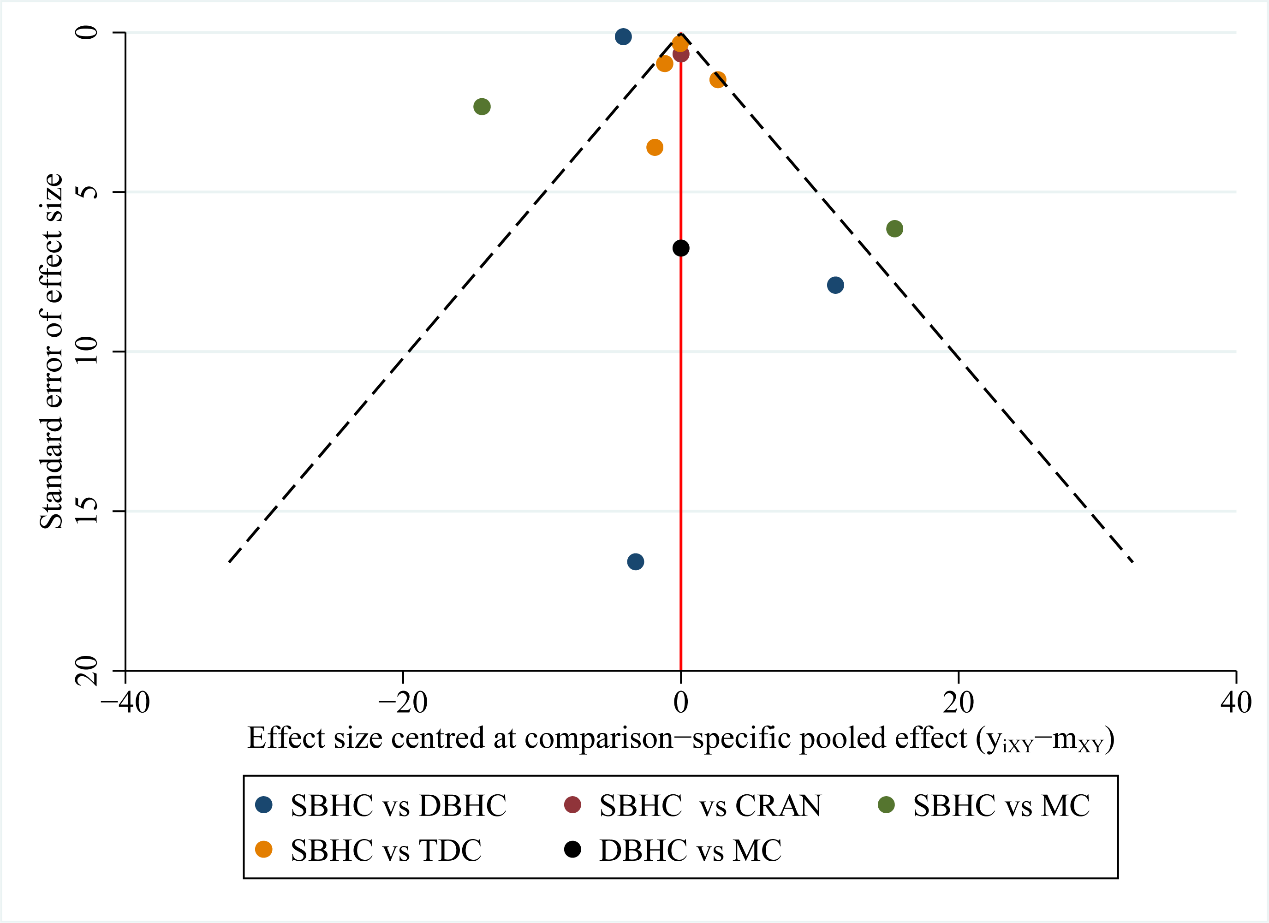


**Figure S16: Funnel plot for Operation time.**


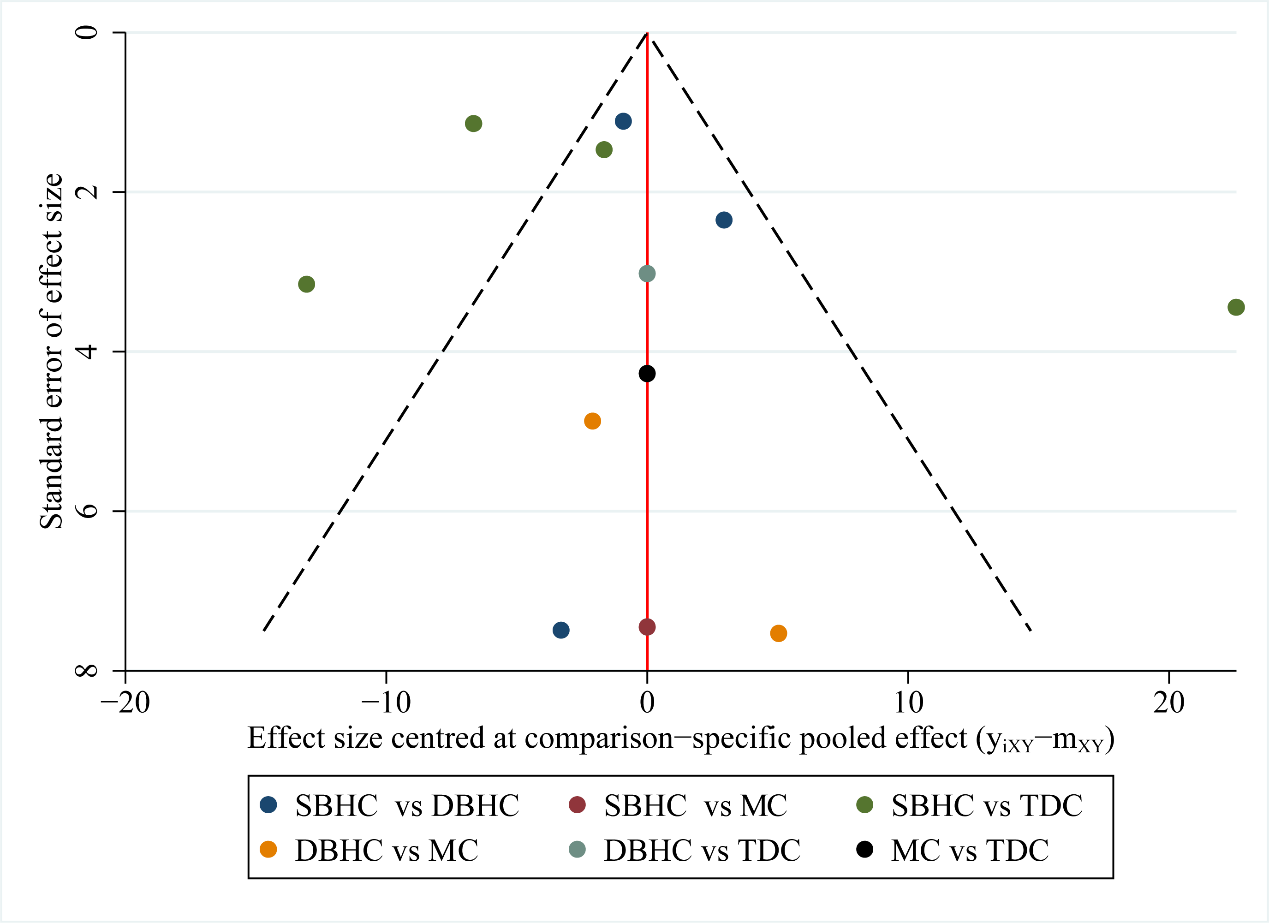


**Figure S17: Funnel plot for Complication.**


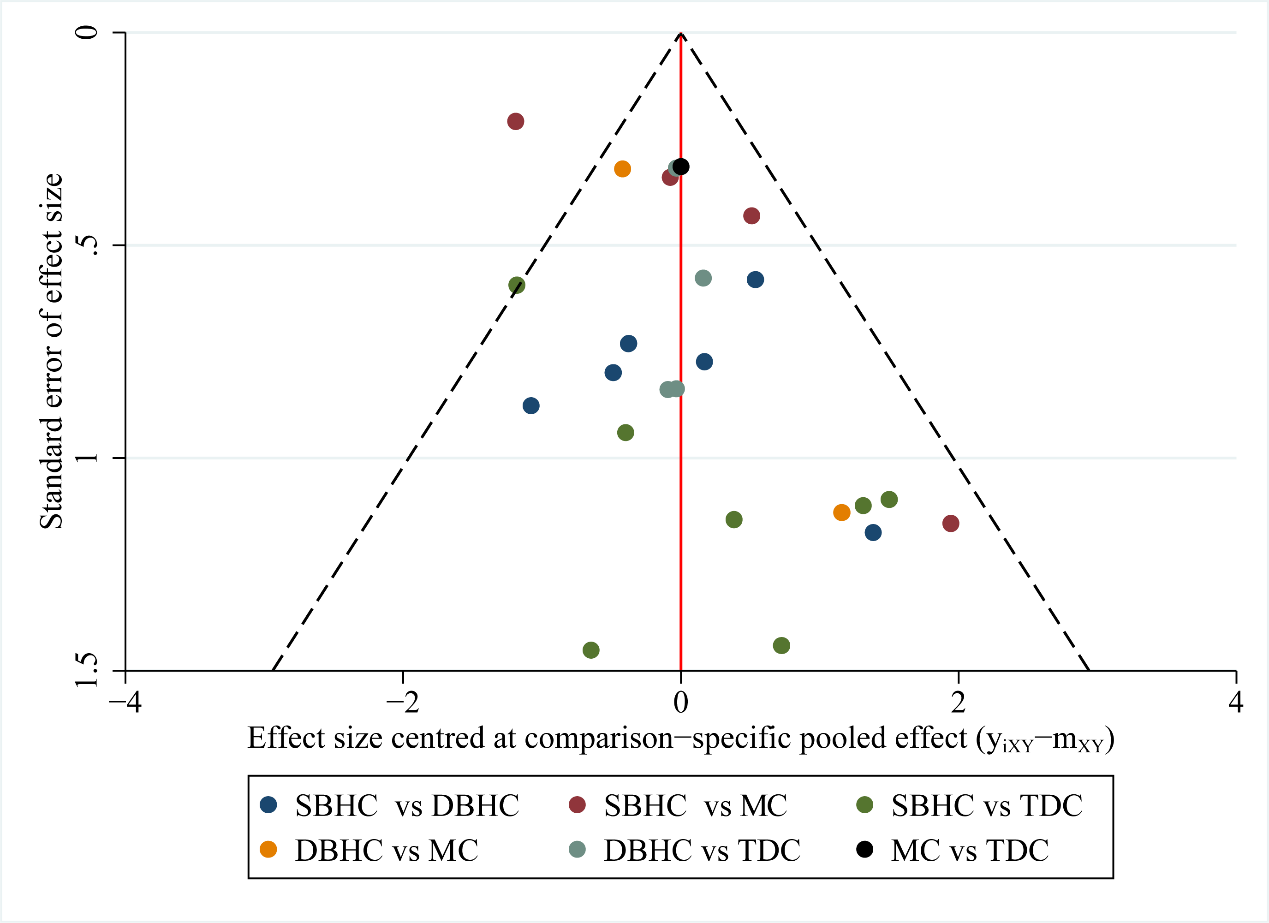


**Figure S18: Funnel plot for Mortality.**


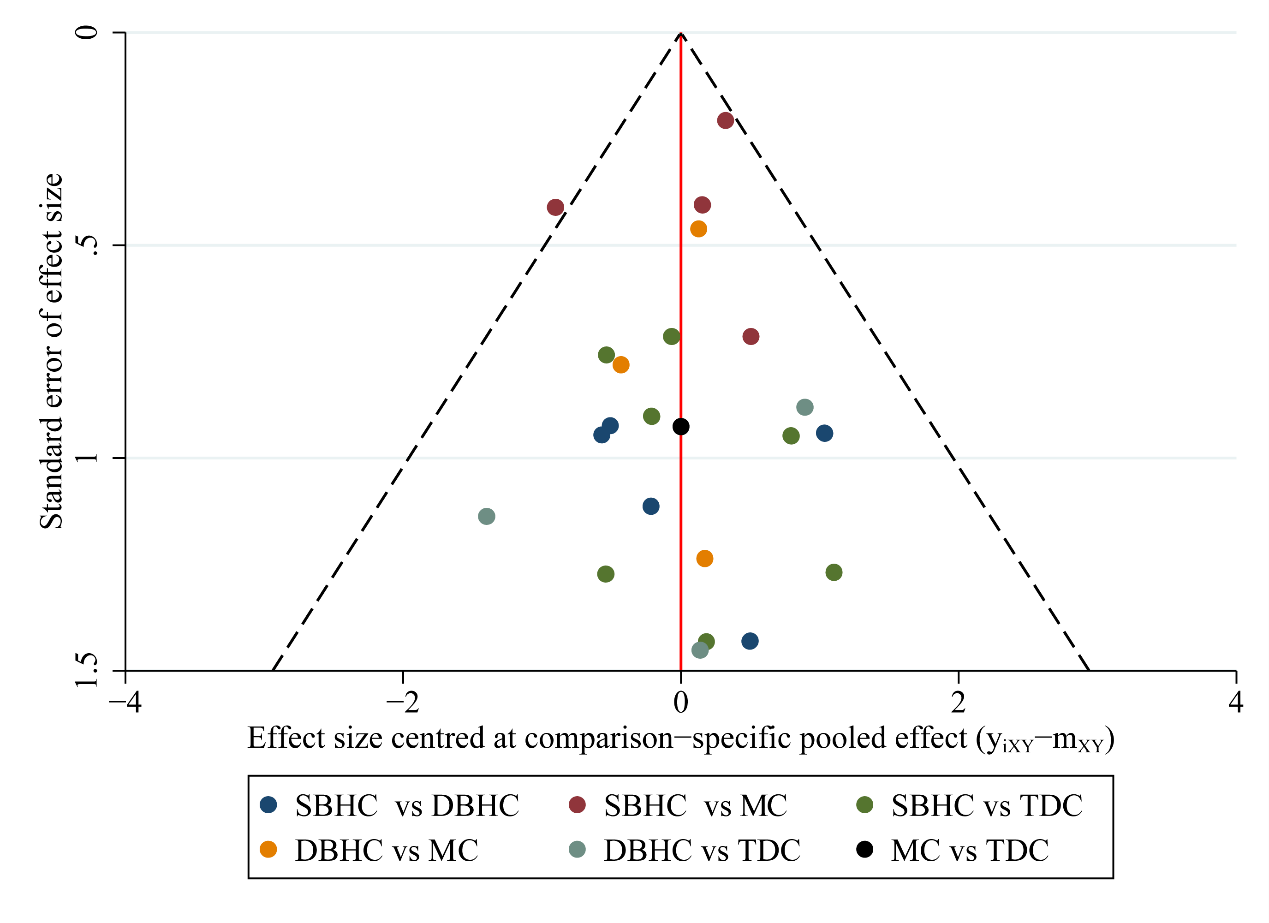


Figure S19: Forest plots of the network meta-analysis: recurrence.


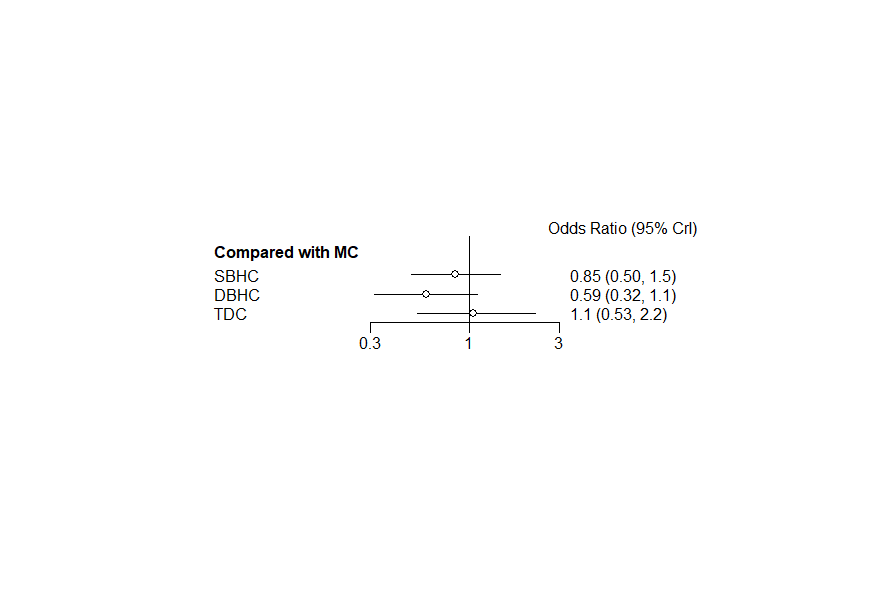

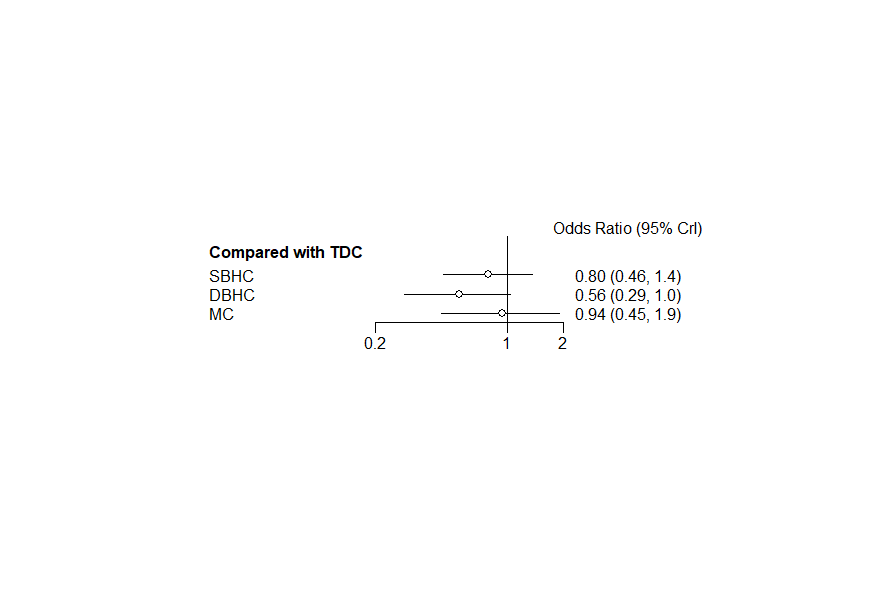

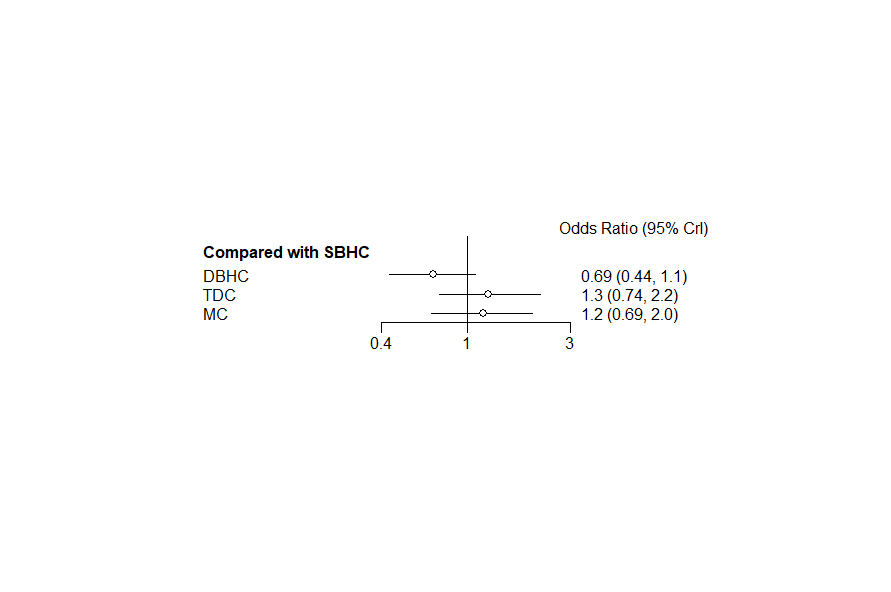

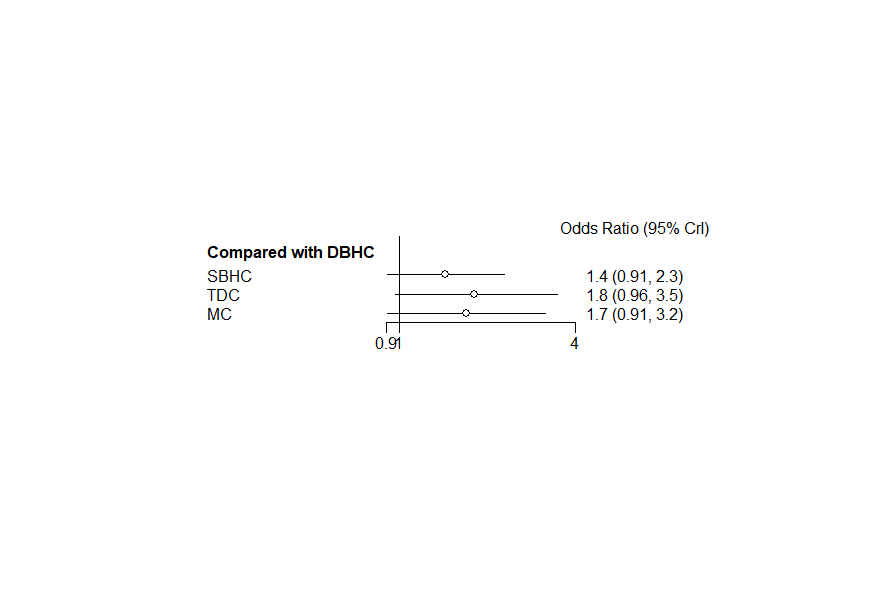


Figure S20: Forest plots of the network meta-analysis: Reoperation.


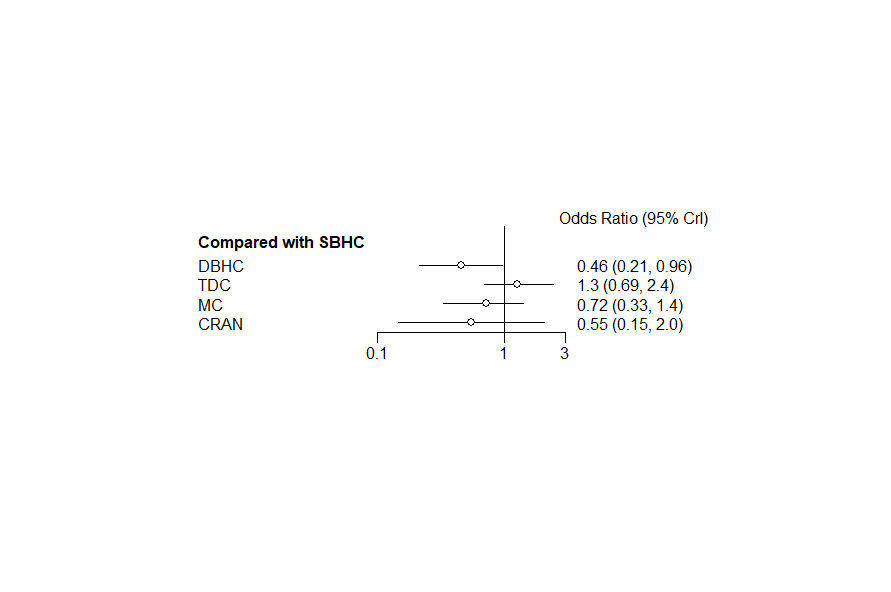

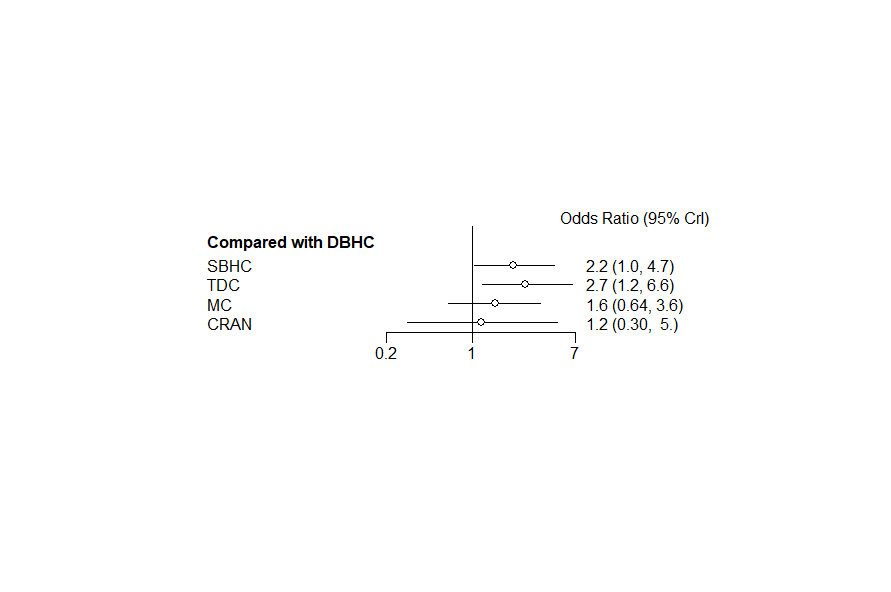

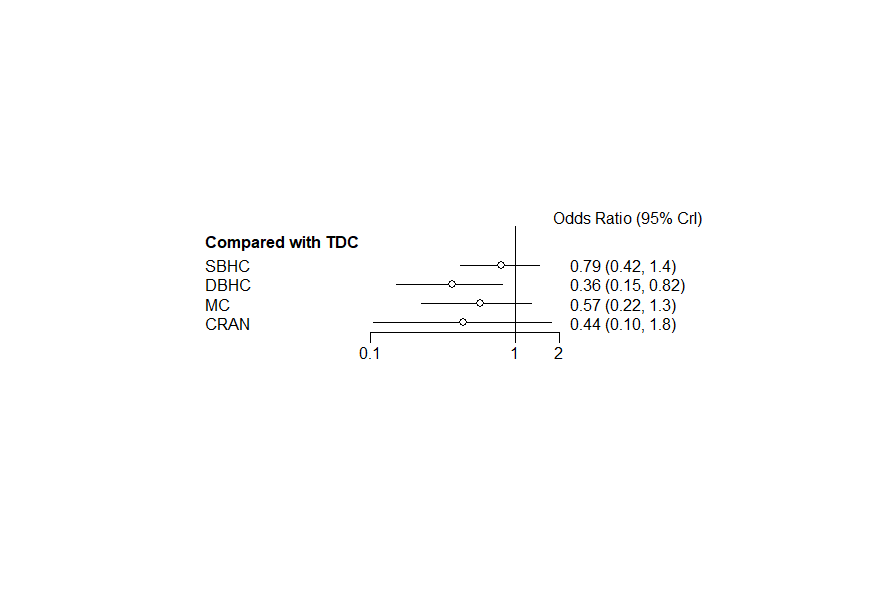

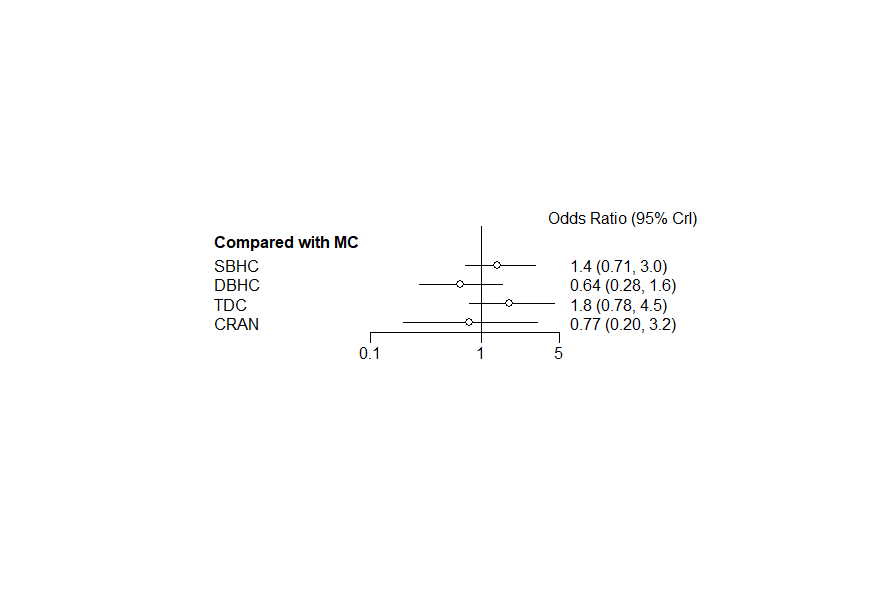

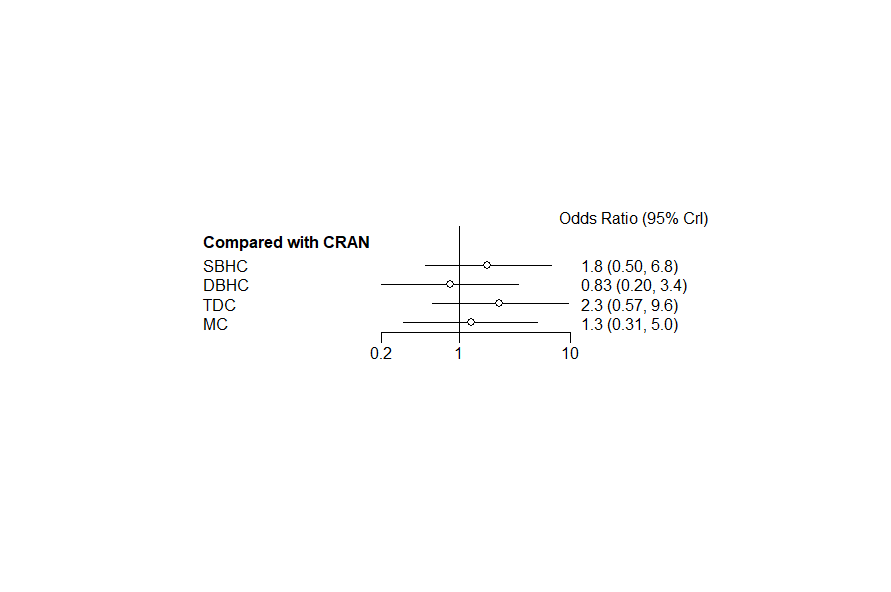


Figure S21: Forest plots of the network meta-analysis: Favorable outcome.


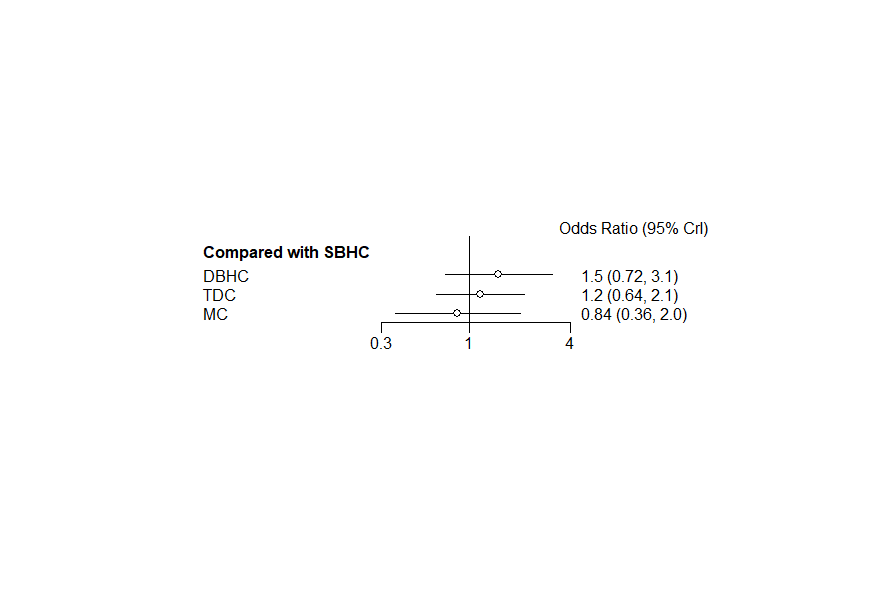

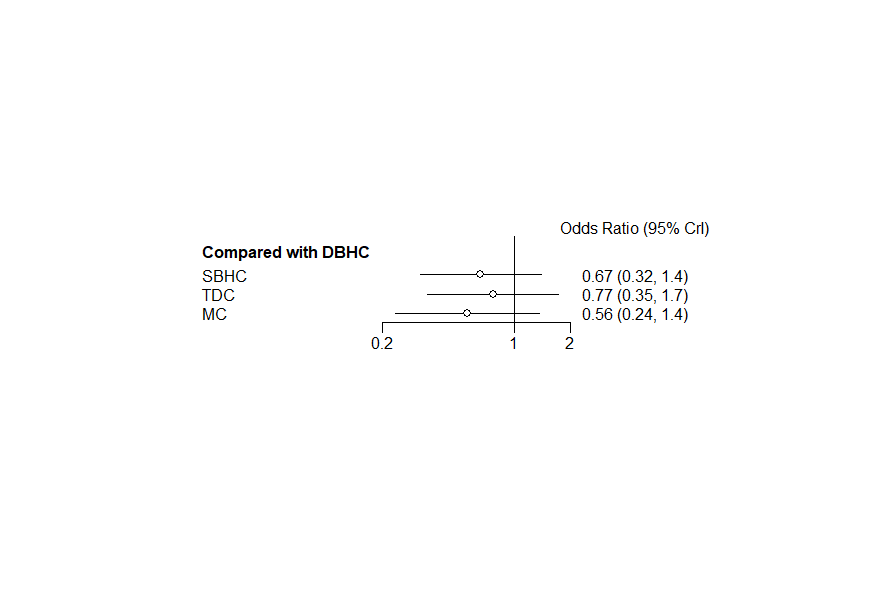

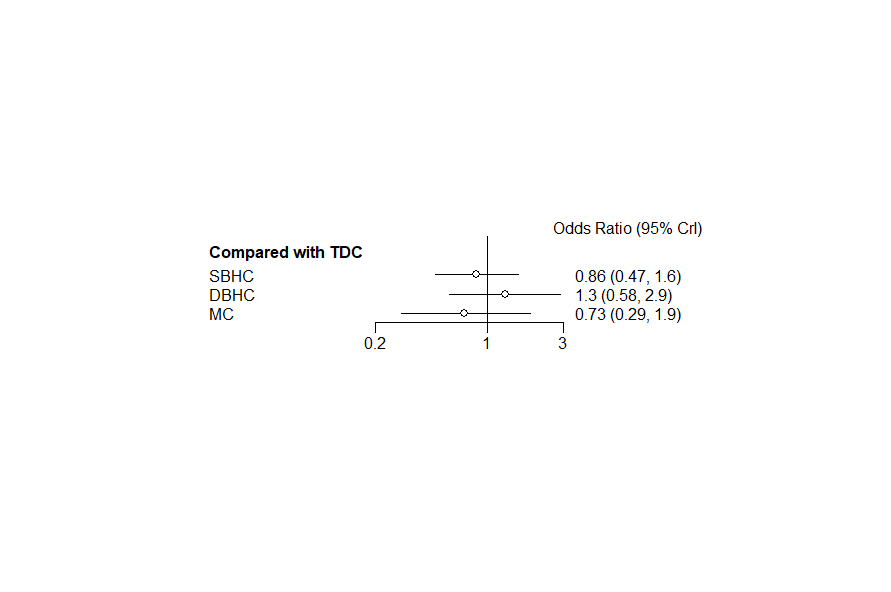

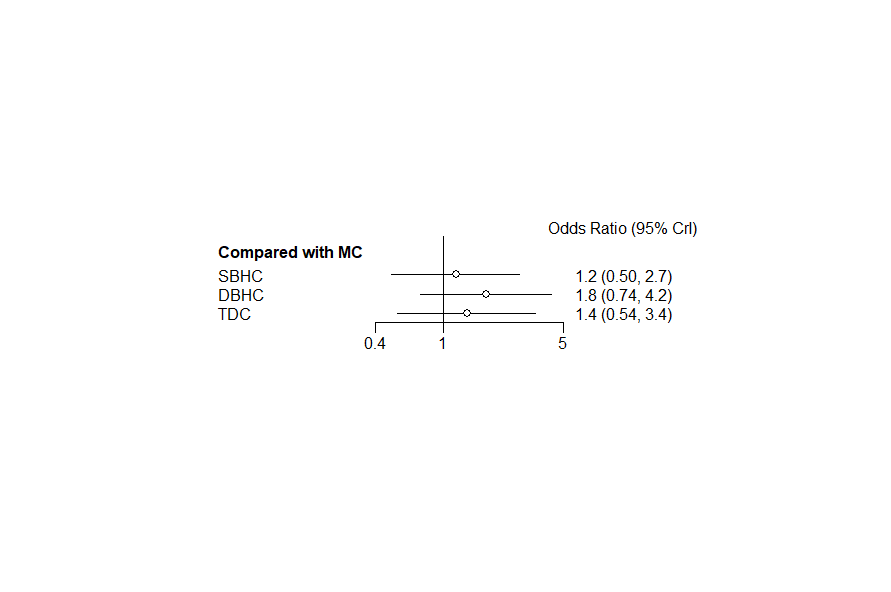


Figure S22: Forest plots of the network meta-analysis: LOS.


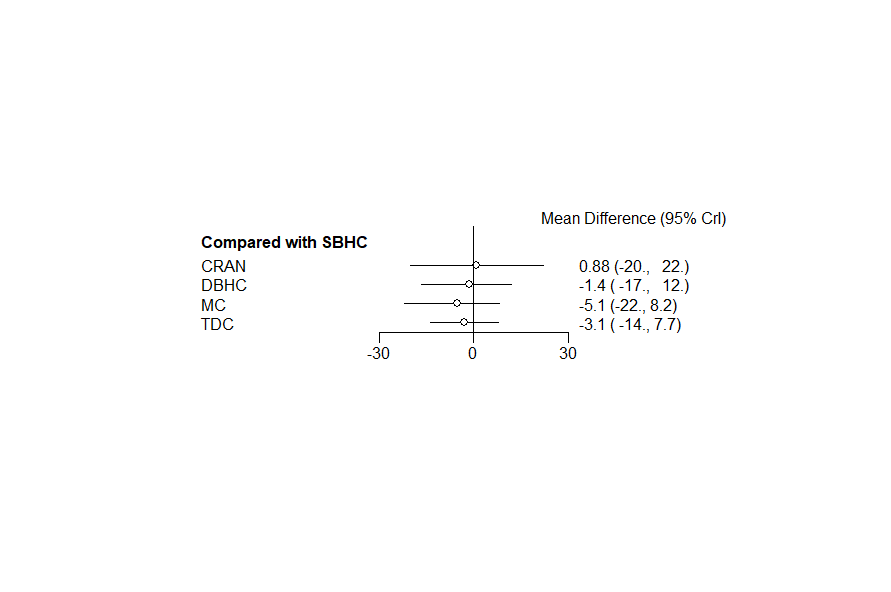

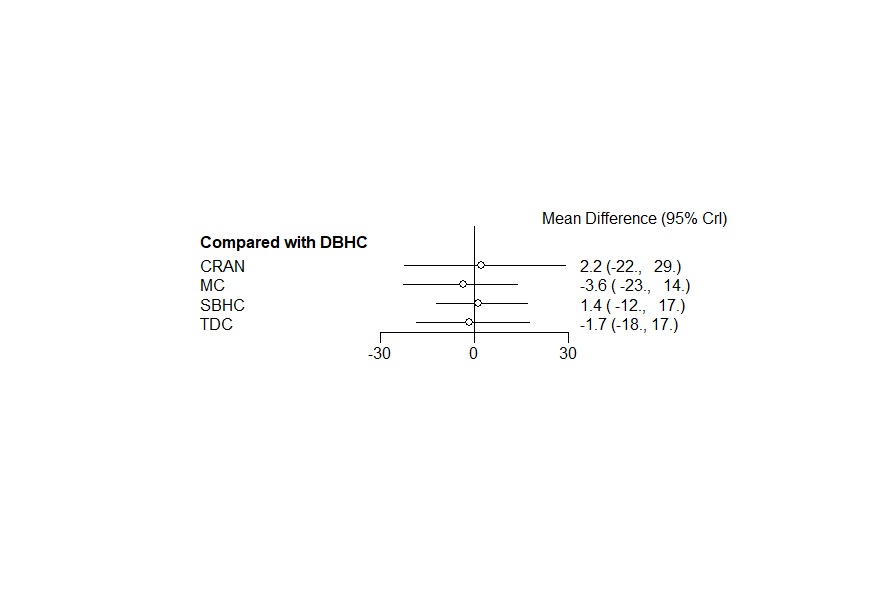

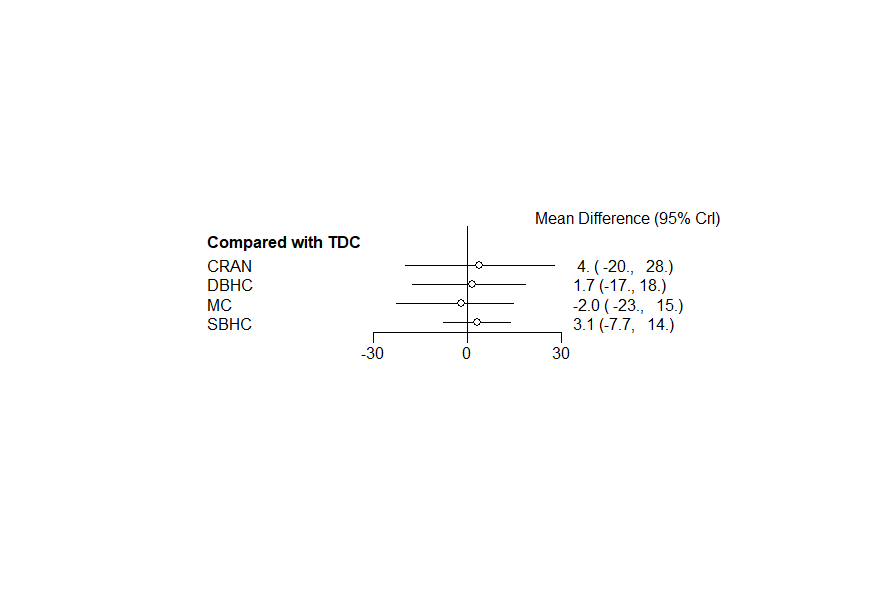

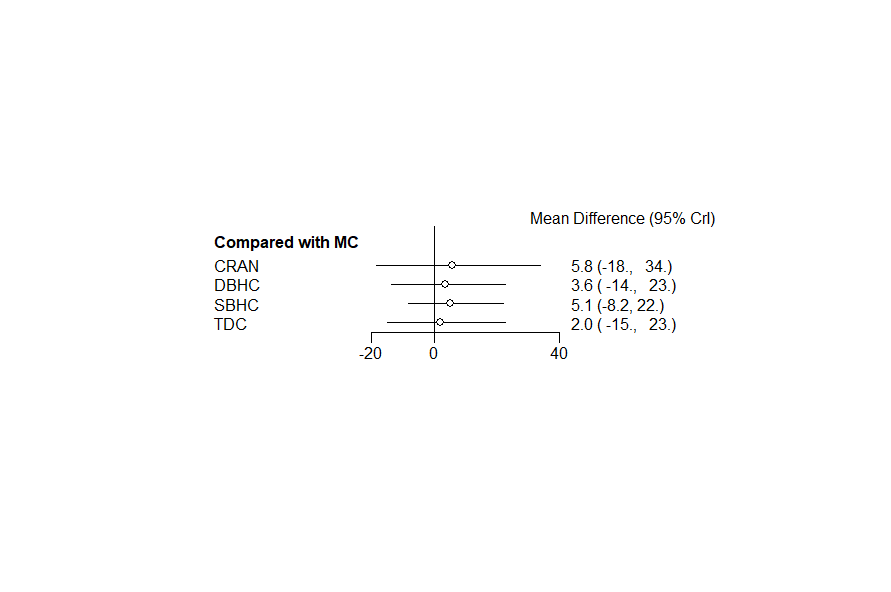

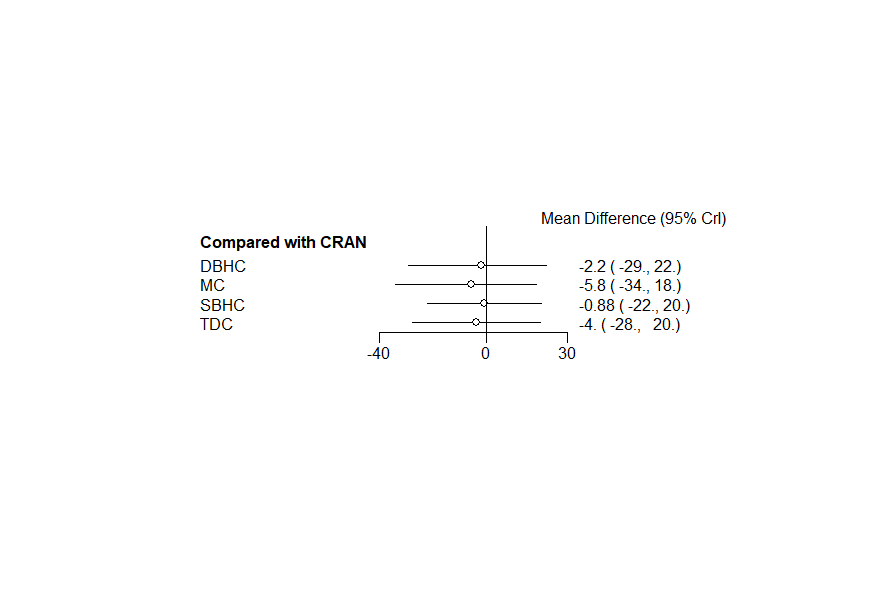


Figure S23: Forest plots of the network meta-analysis: Operation time.


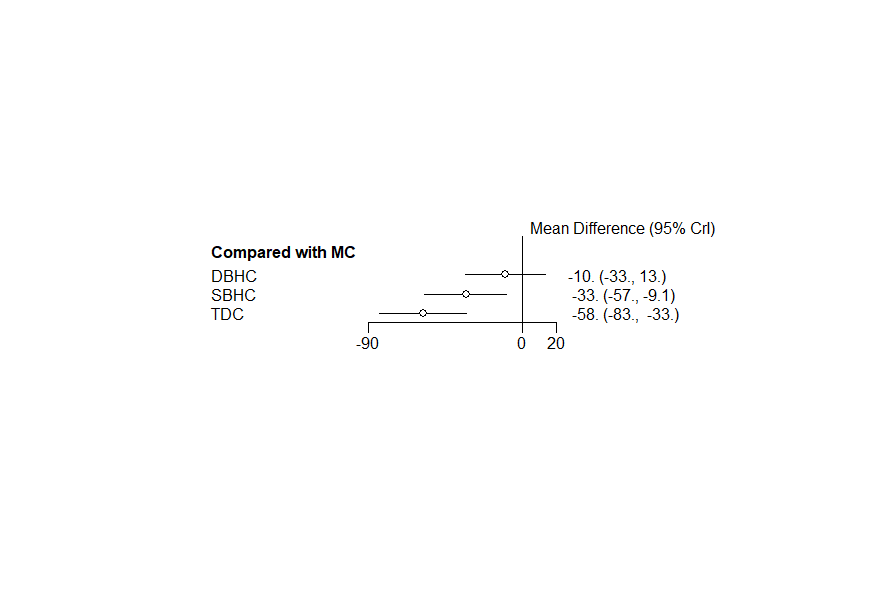

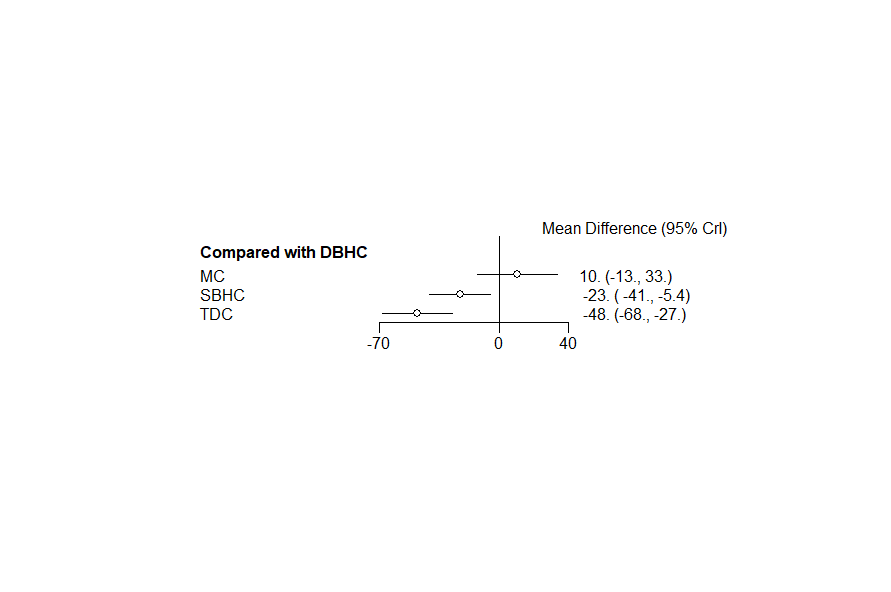

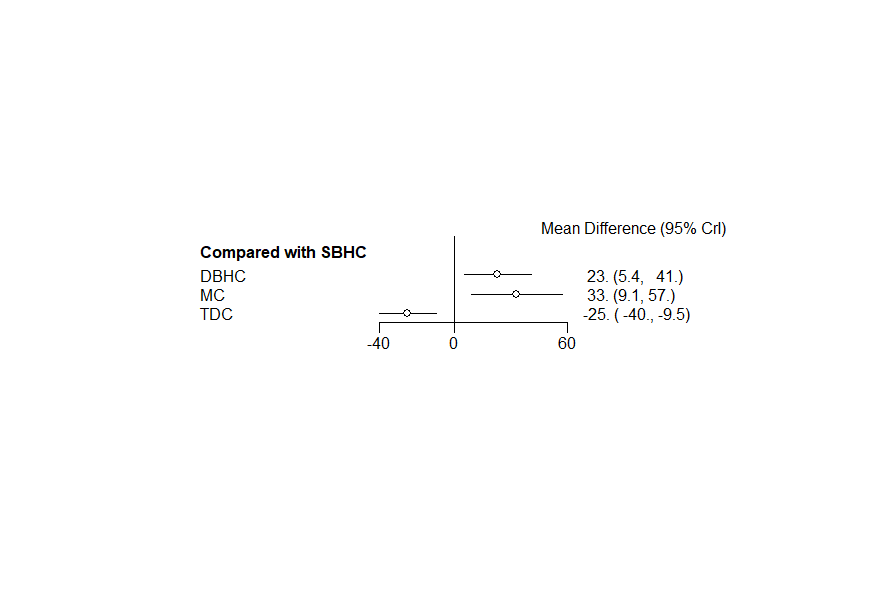

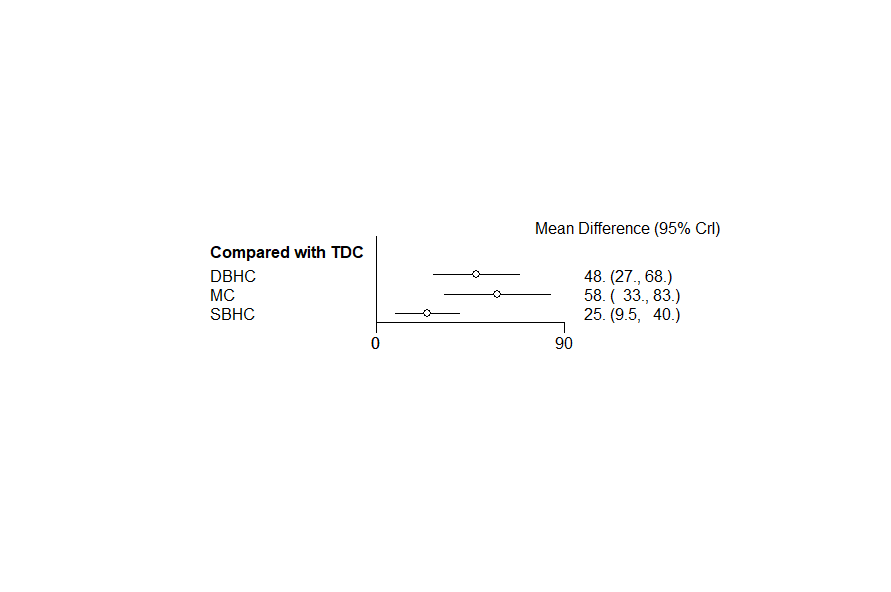


Figure S24: Forest plots of the network meta-analysis: Complication.


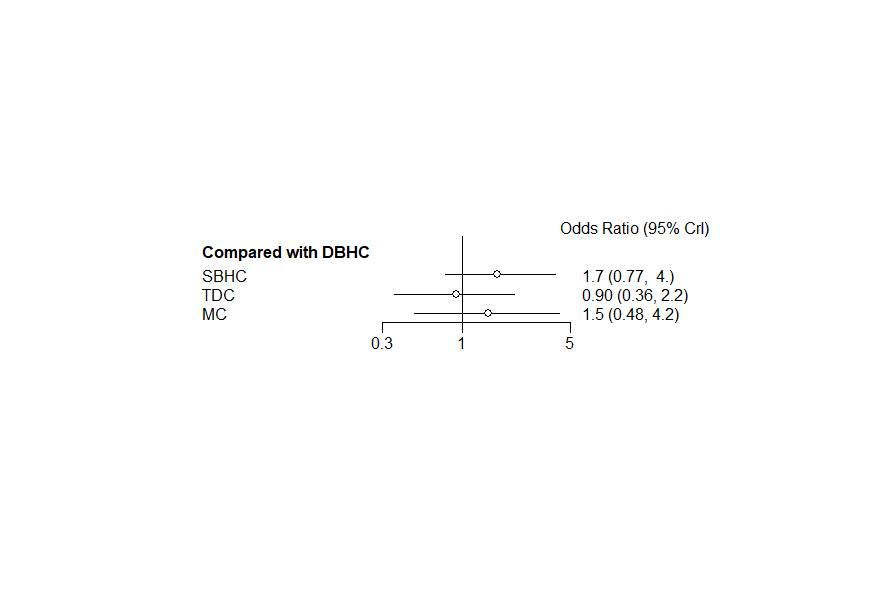

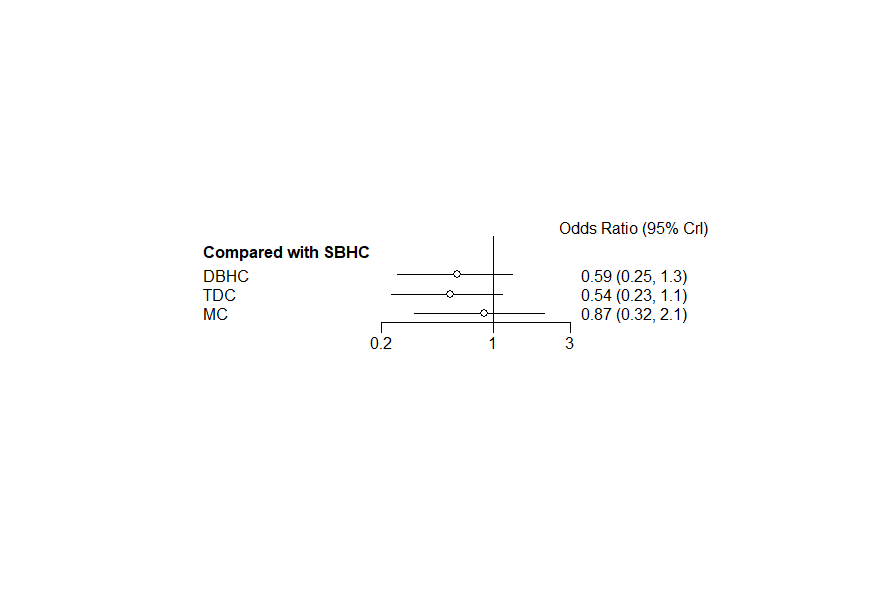

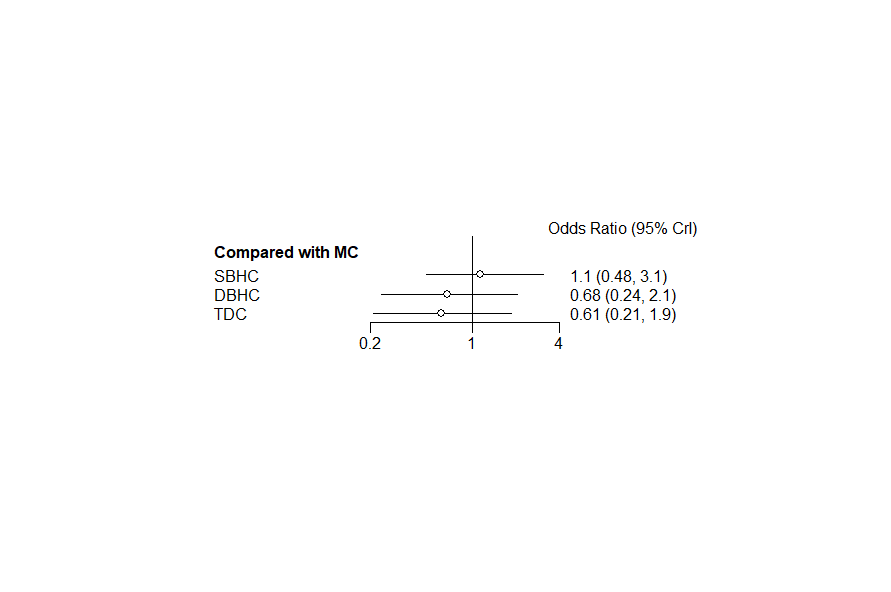

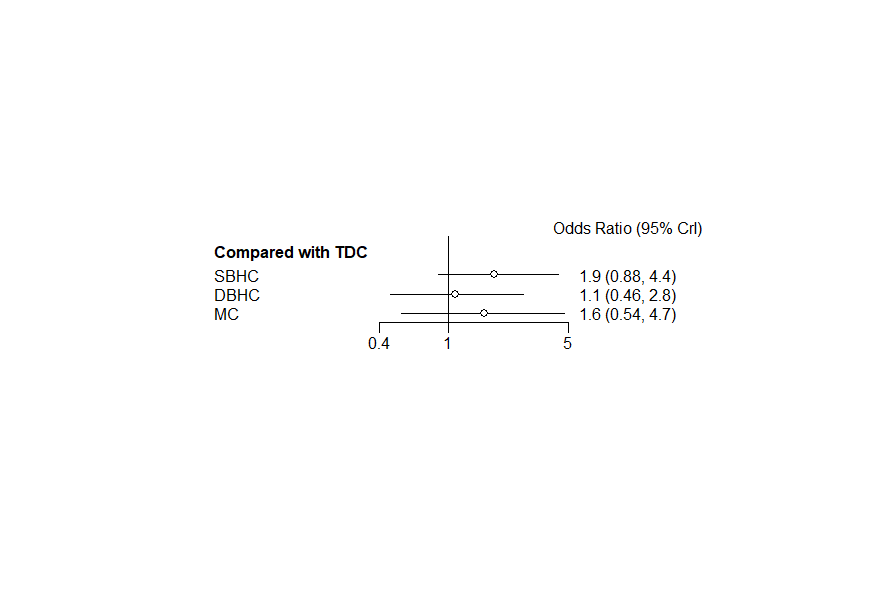


Figure S25: Forest plots of the network meta-analysis: Mortality.


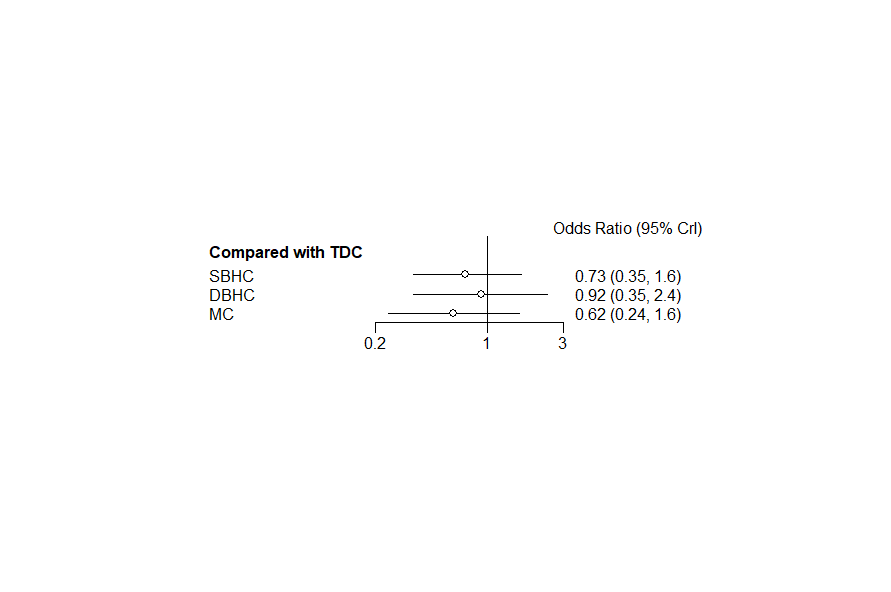

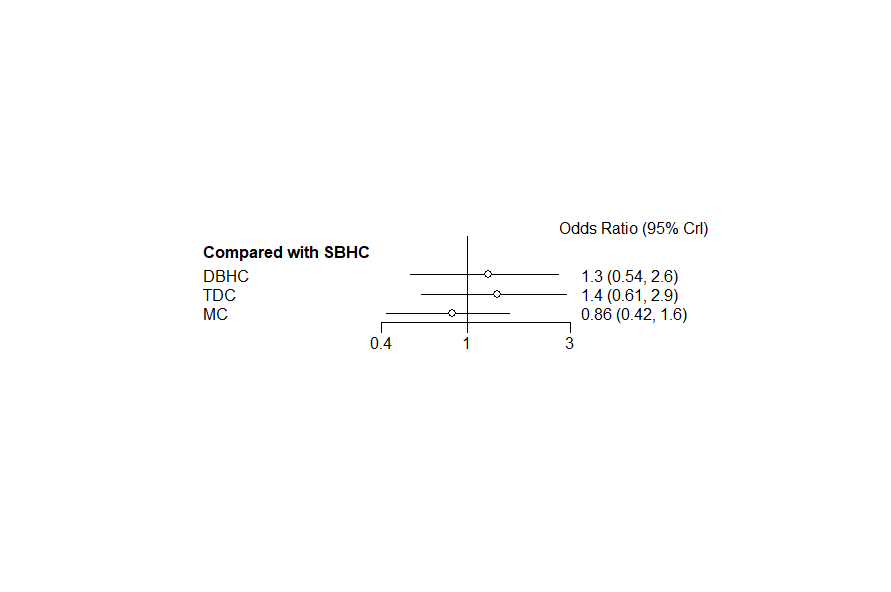

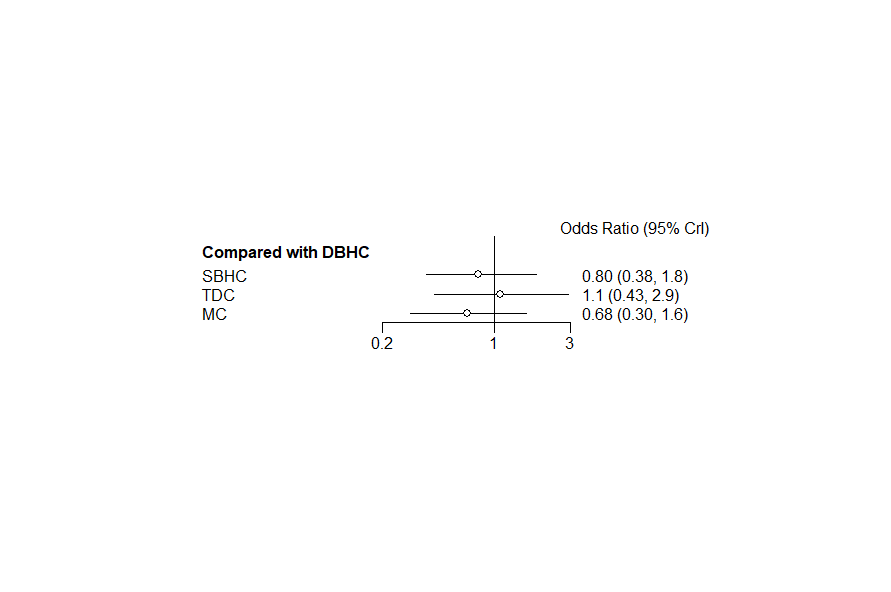

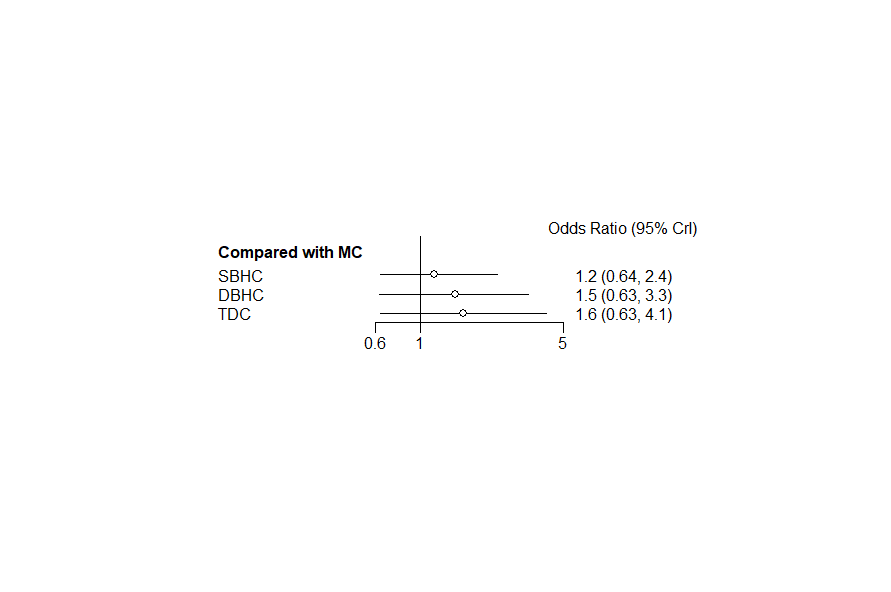


Figure S26: Convergence diagnostics of the network meta-analysis: Recurrence.


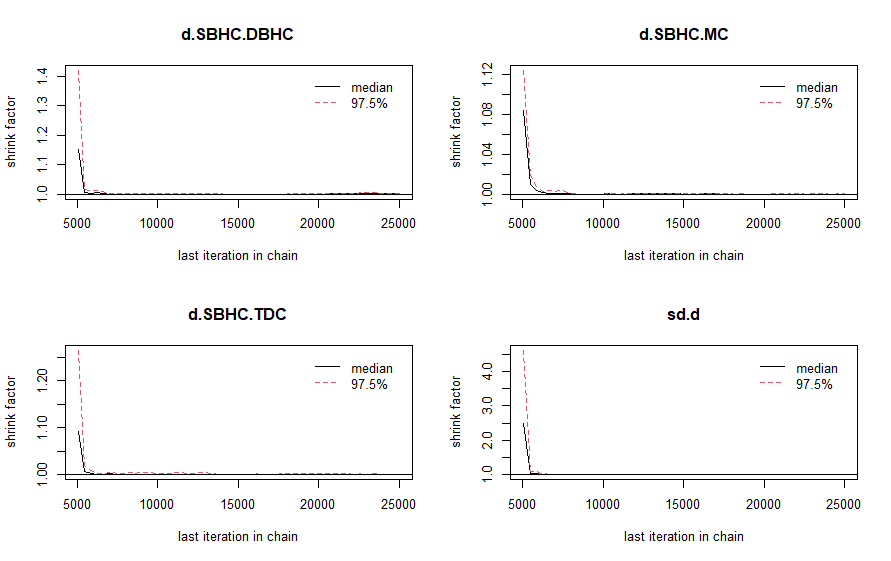


Figure S27: Convergence diagnostics of the network meta-analysis: Reoperation.


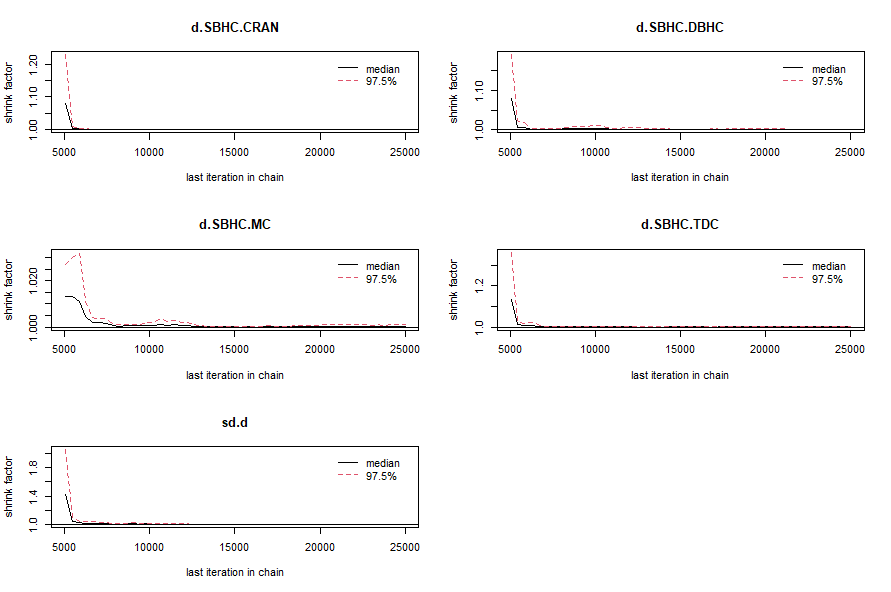


Figure S28: Convergence diagnostics of the network meta-analysis: Favorable outcome.


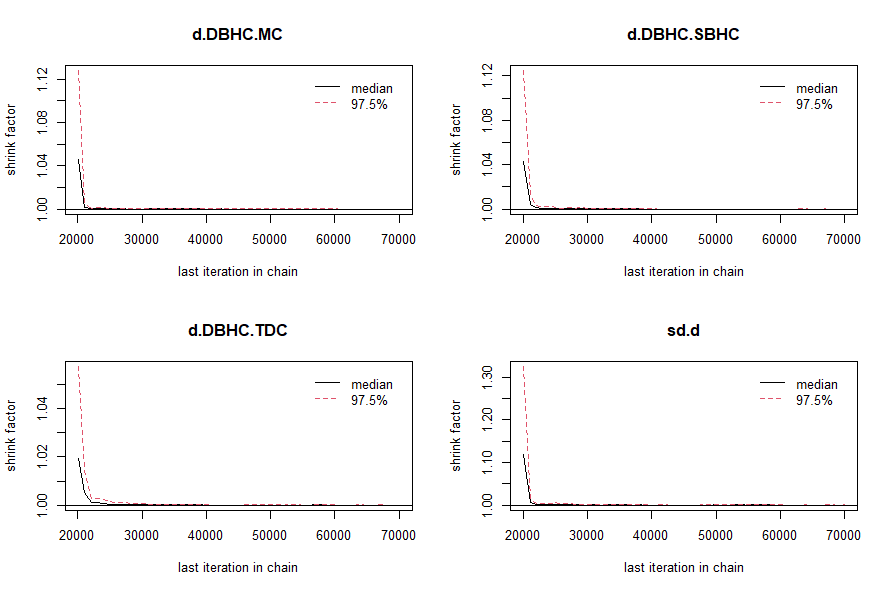


Figure S29: Convergence diagnostics of the network meta-analysis: LOS.


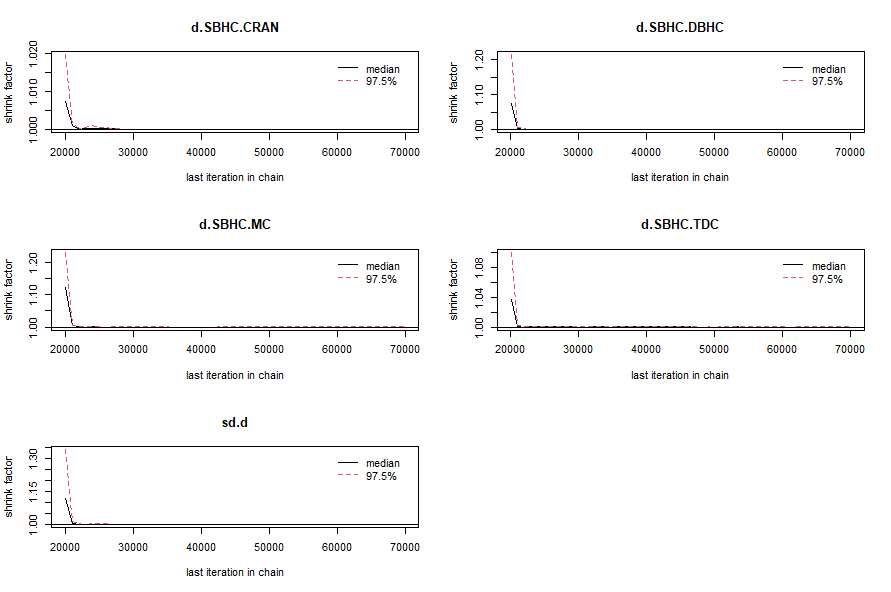


Figure S30: Convergence diagnostics of the network meta-analysis: Operation time.


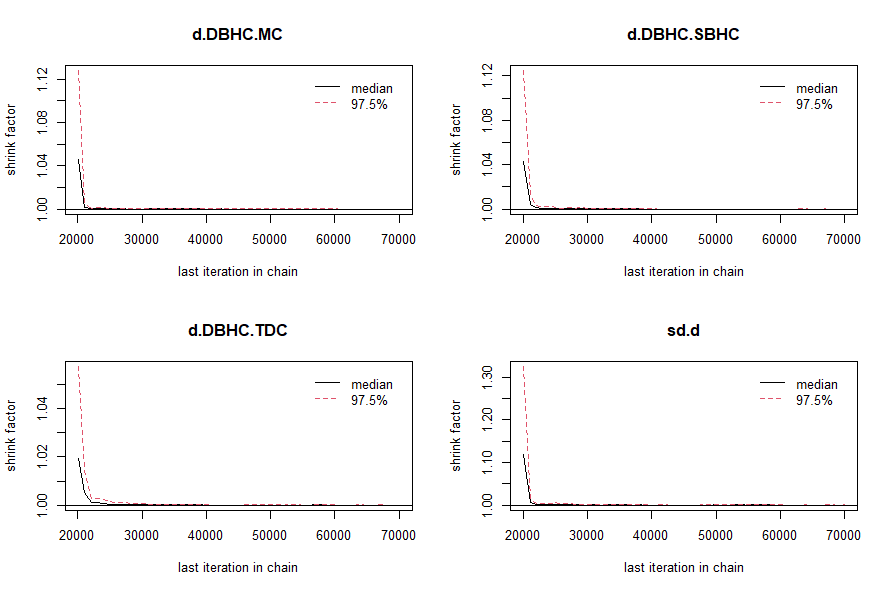


Figure S31: Convergence diagnostics of the network meta-analysis: Complication.


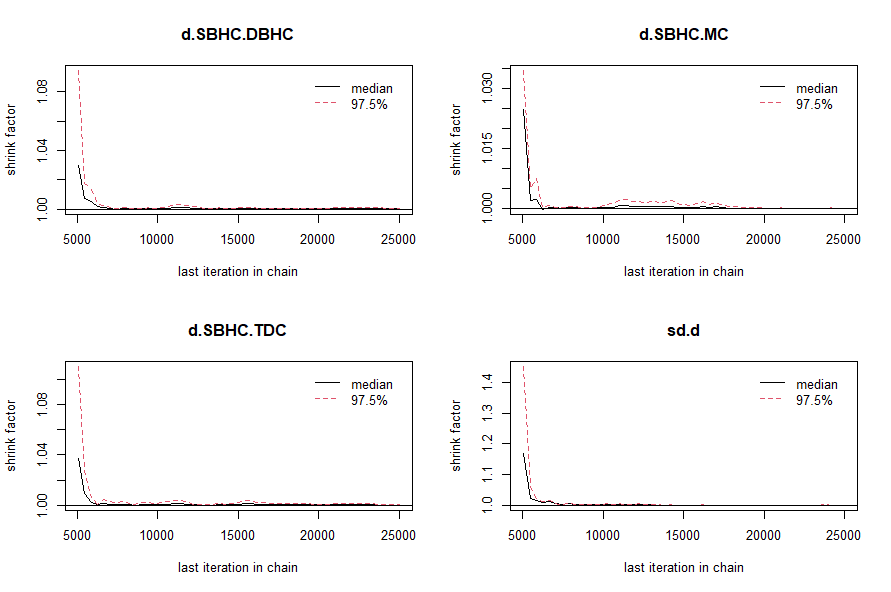


Figure S32: Convergence diagnostics of the network meta-analysis: Mortality.


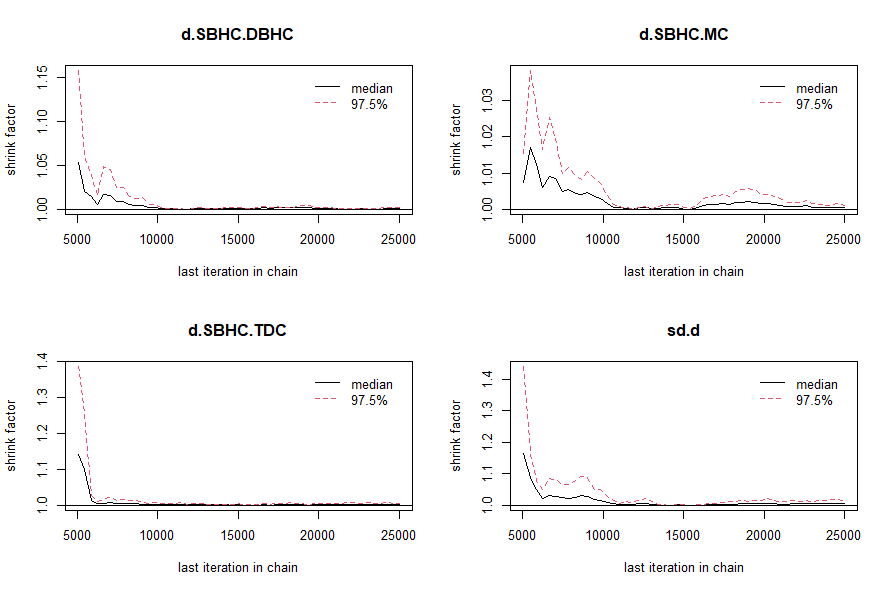


Figure S33: Trace and density of the network meta-analysis: Recurrence.


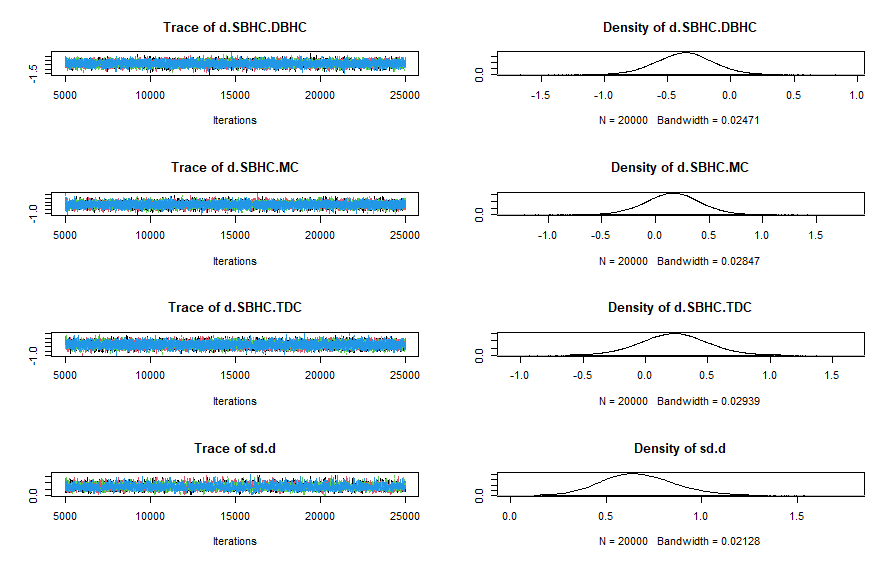


Figure S34: Trace and density of the network meta-analysis: Reoperation.


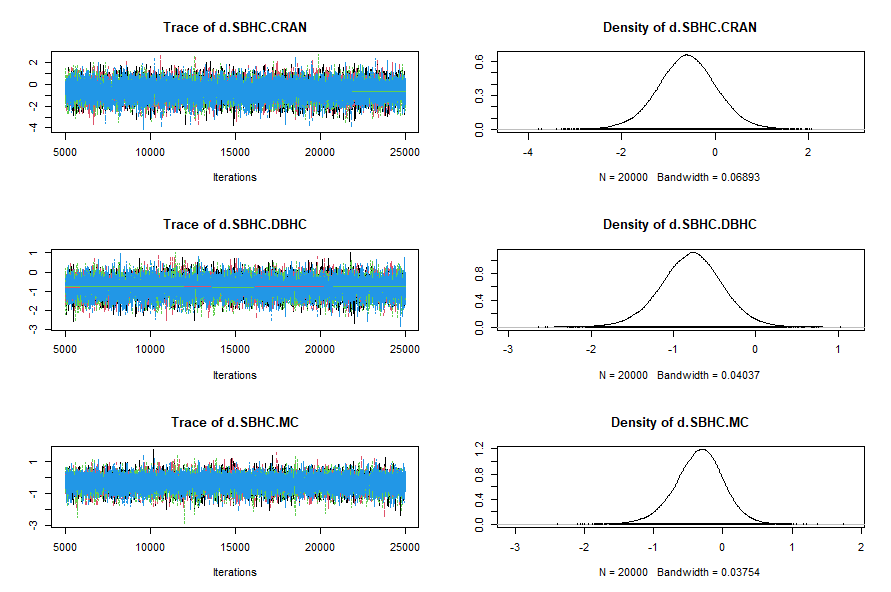

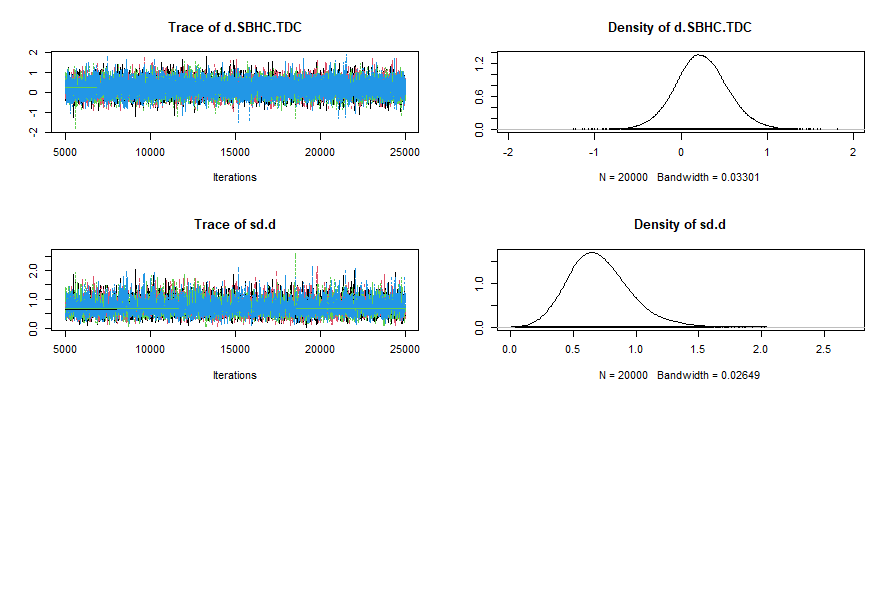


Figure S35: Trace and density of the network meta-analysis: Favorable outcome.


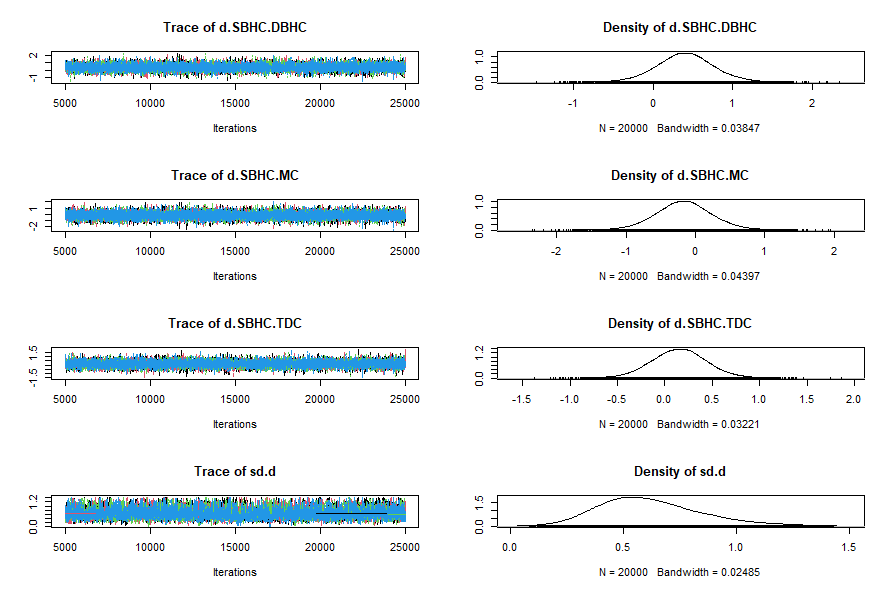


Figure S36: Trace and density of the network meta-analysis: LOS.


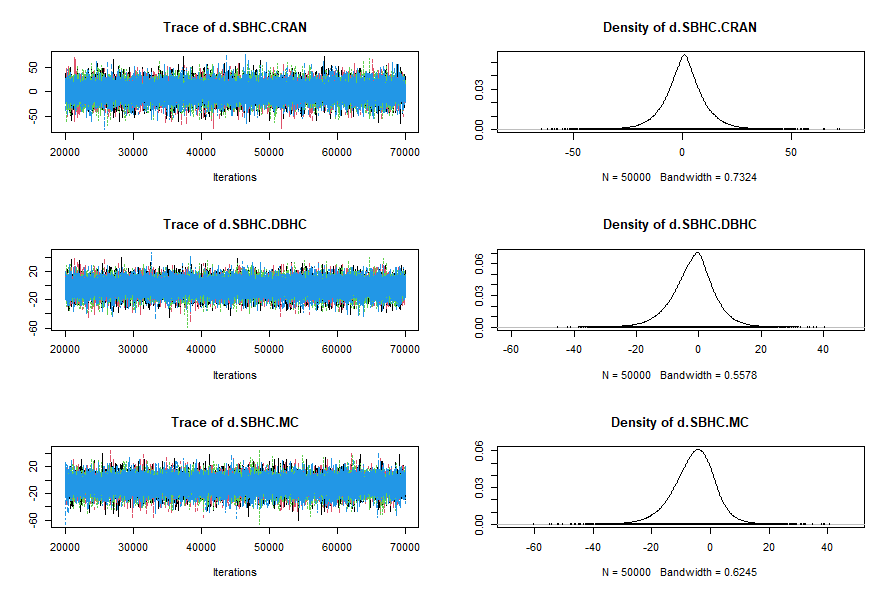

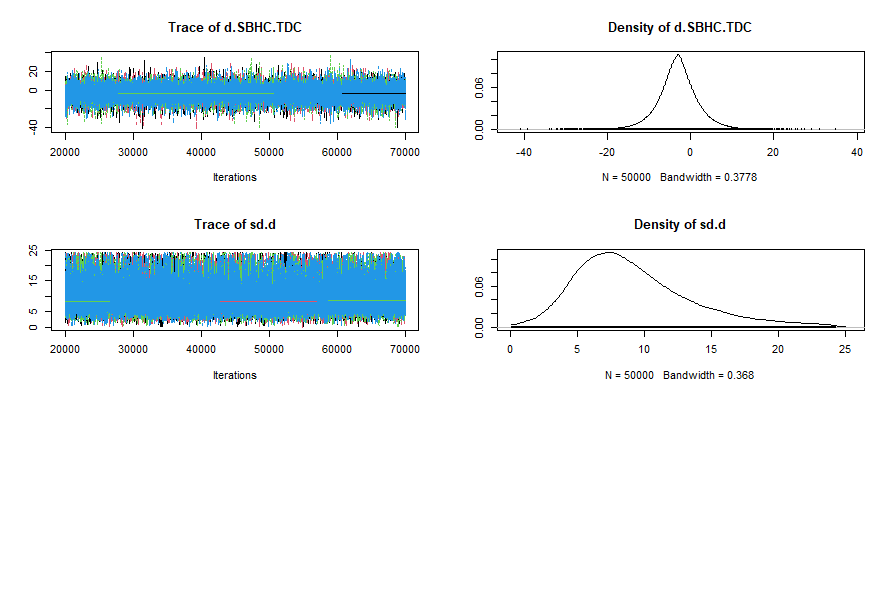


Figure S37: Trace and density of the network meta-analysis: Operation time.


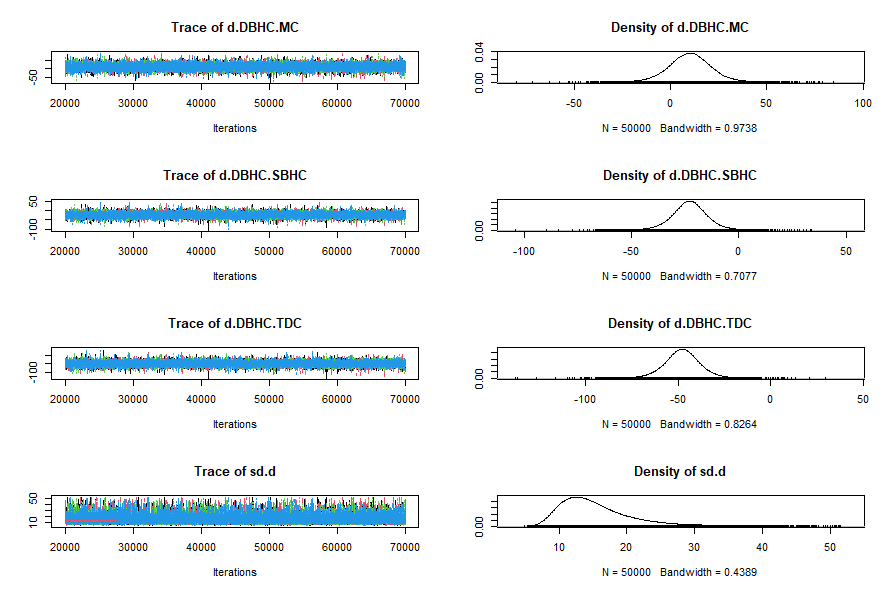


Figure S38: Trace and density of the network meta-analysis: Complication.


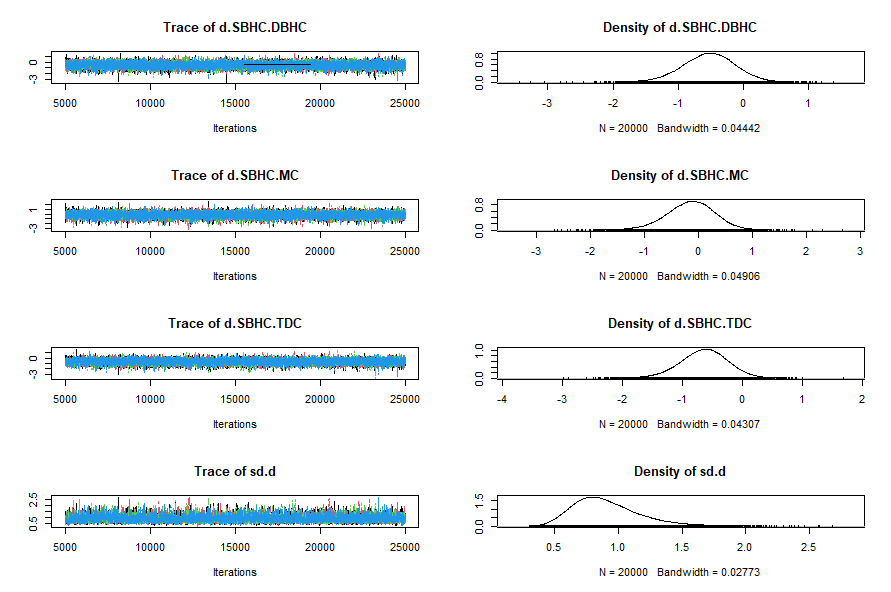


Figure S39: Trace and density of the network meta-analysis: Mortality.


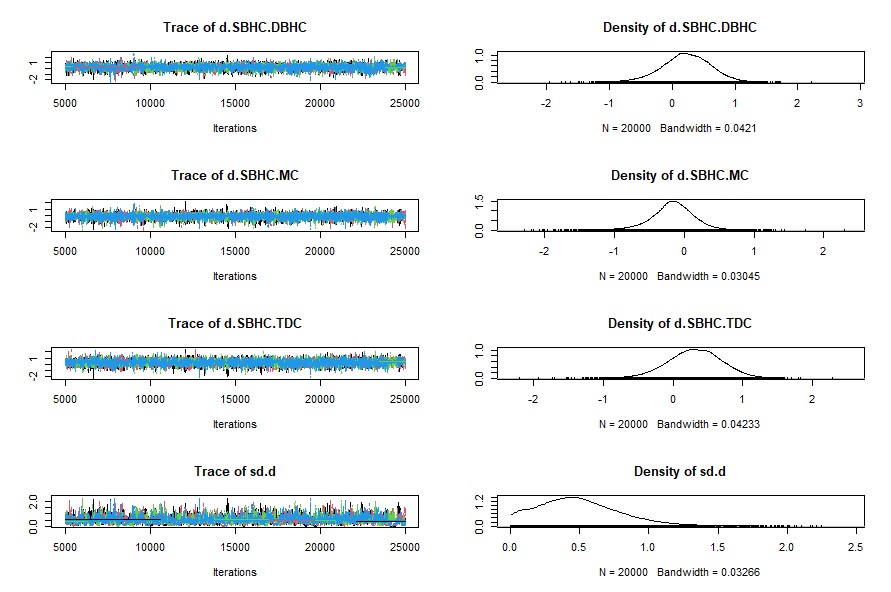


Figure S40: Forest plots for the heterogeneity: Recurrence.


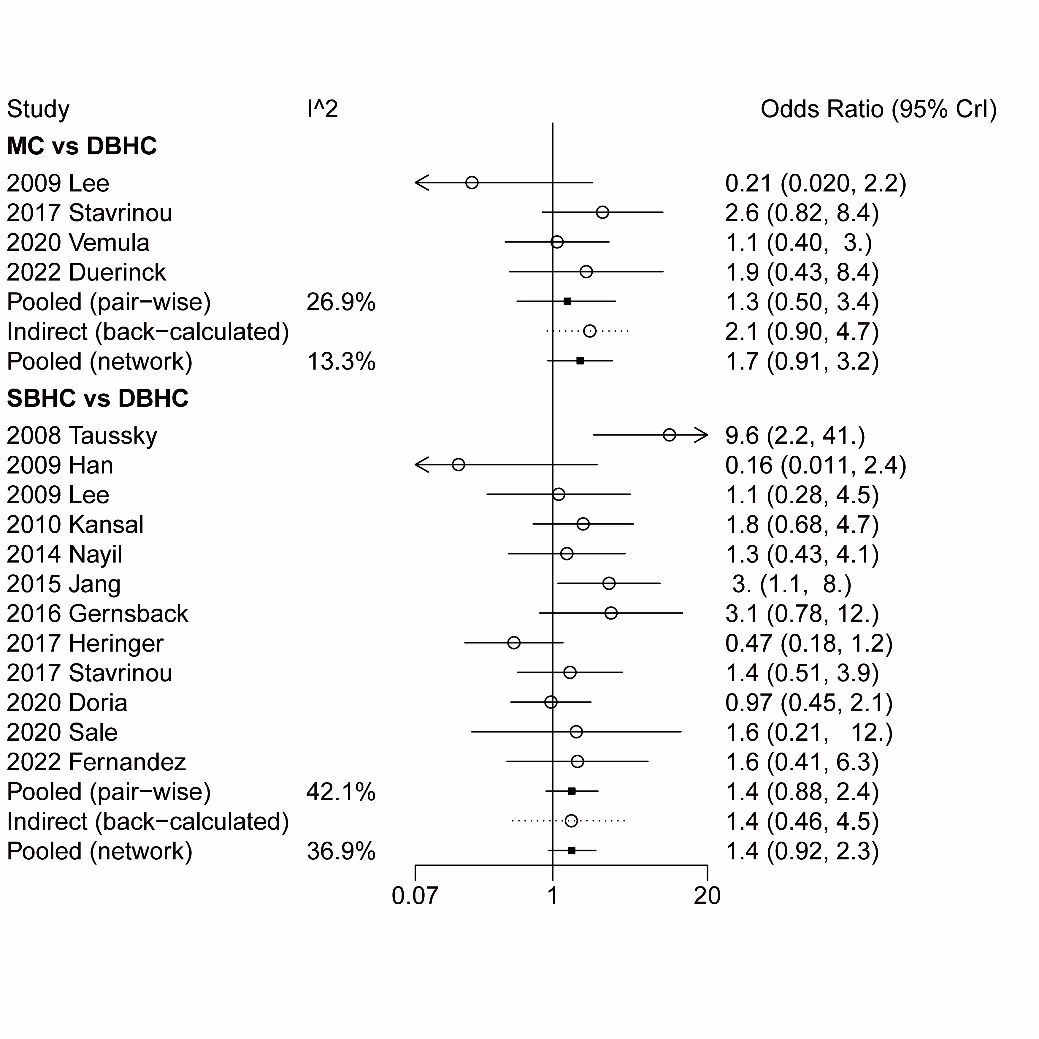

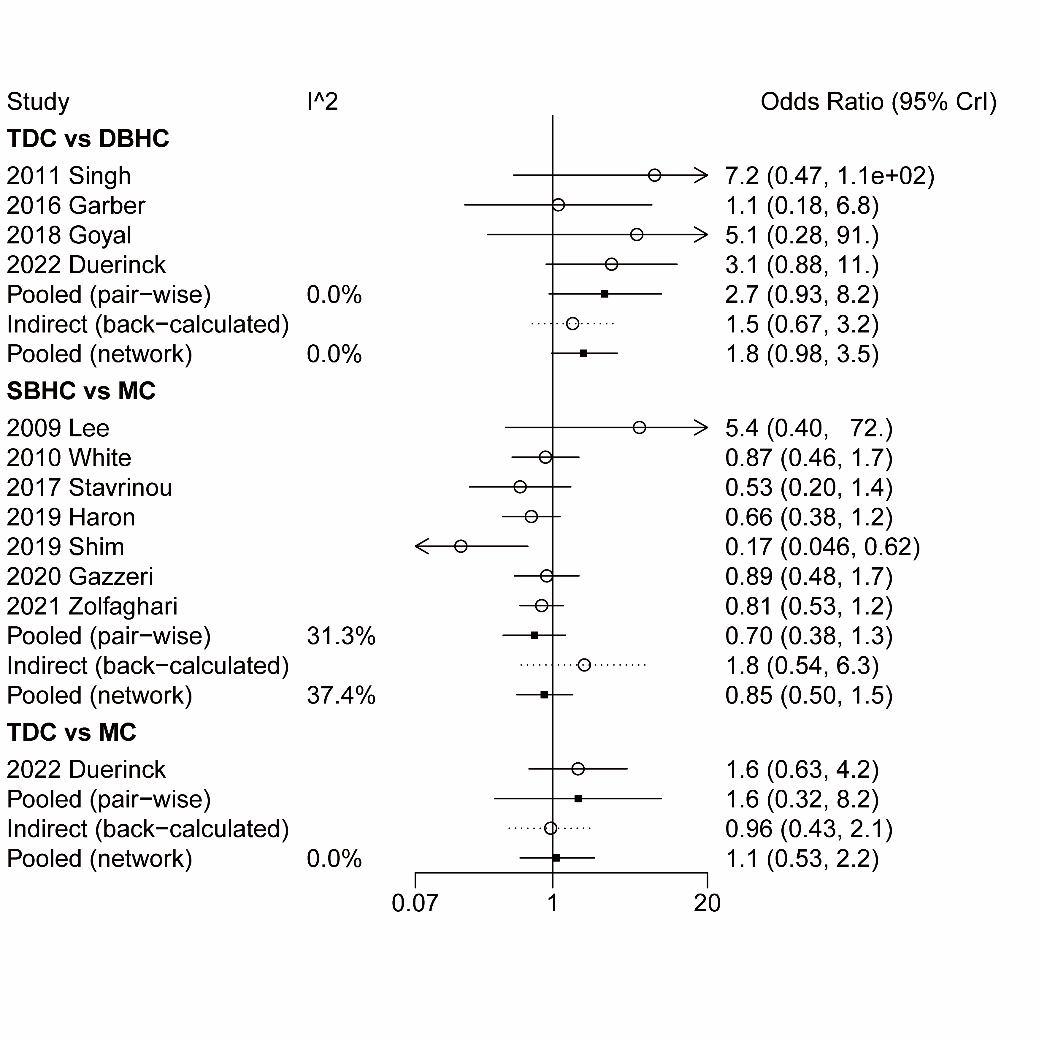


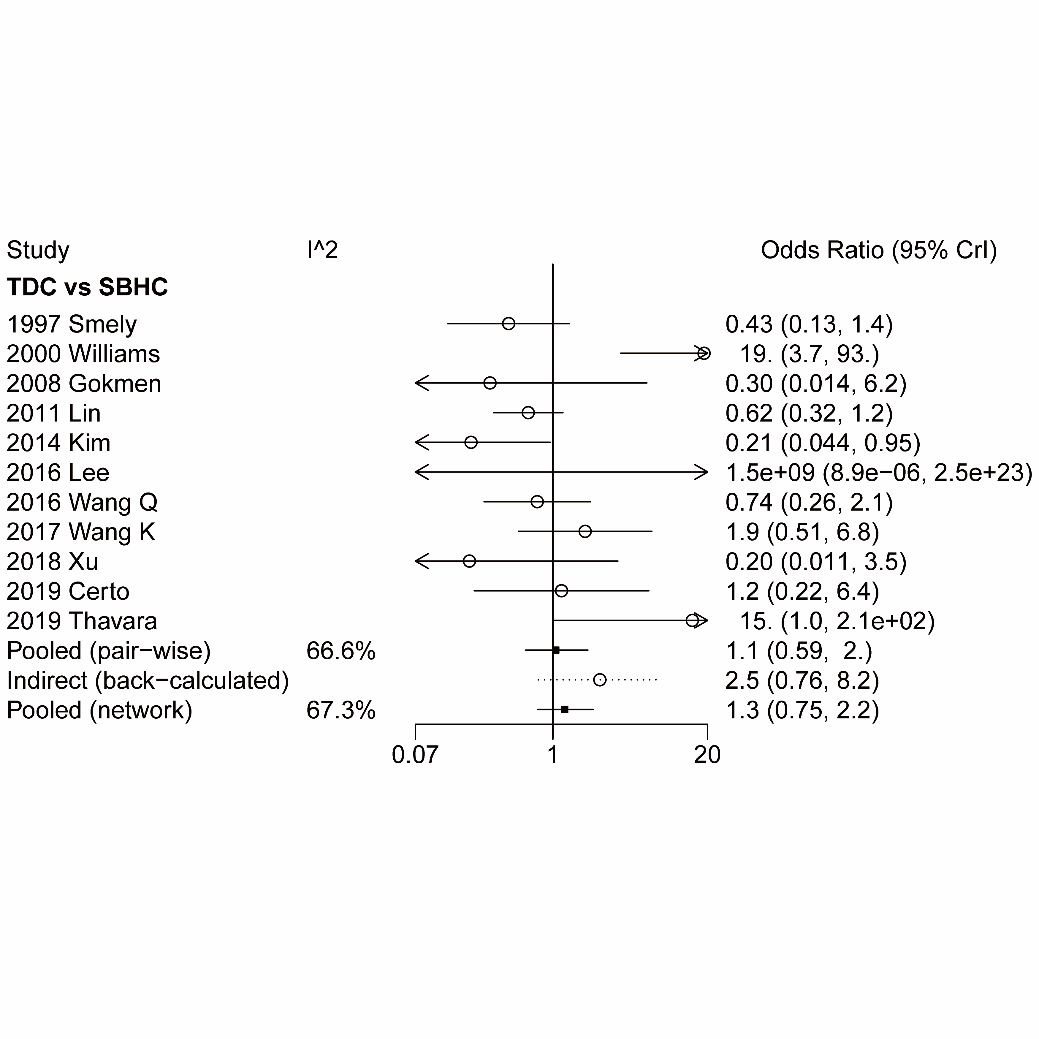


Figure S41: Forest plots for the heterogeneity: Reoperation.


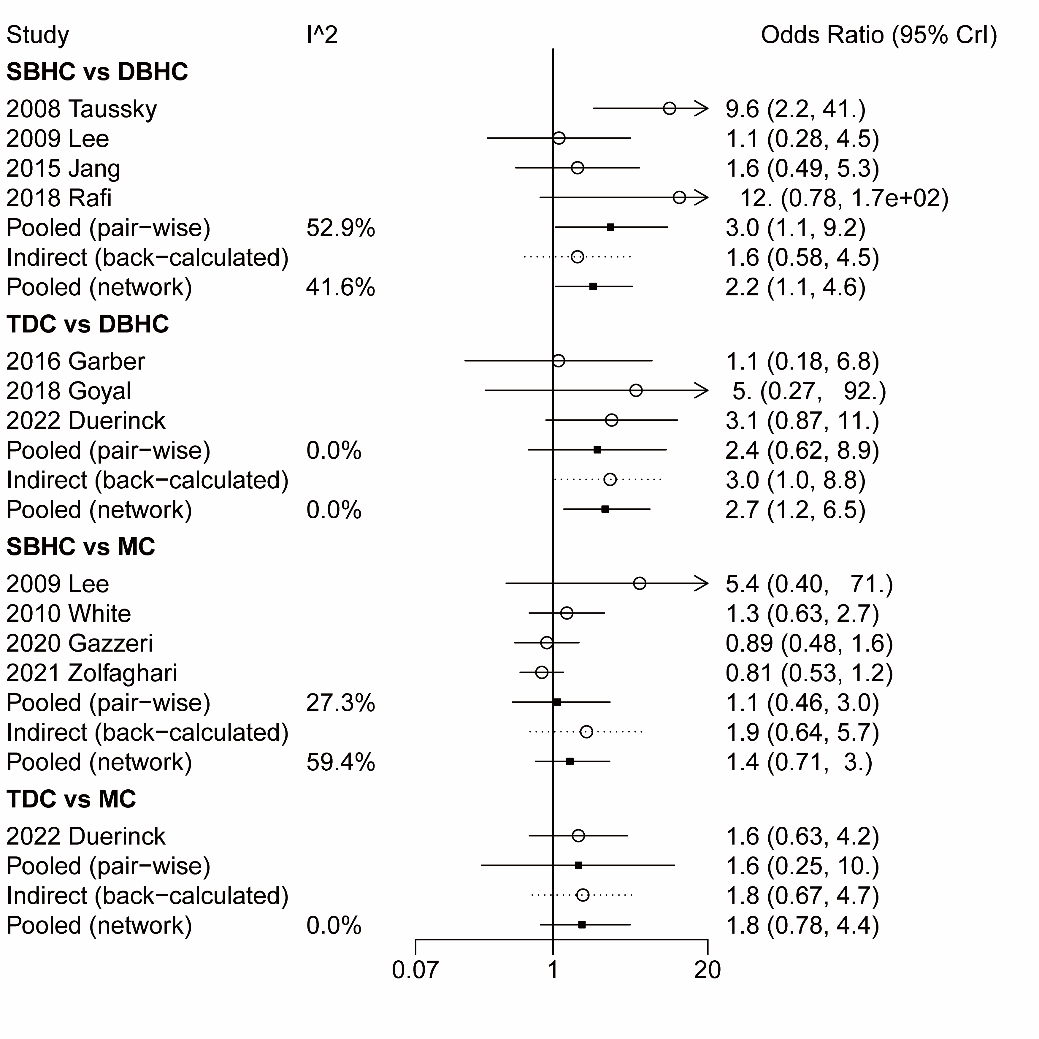

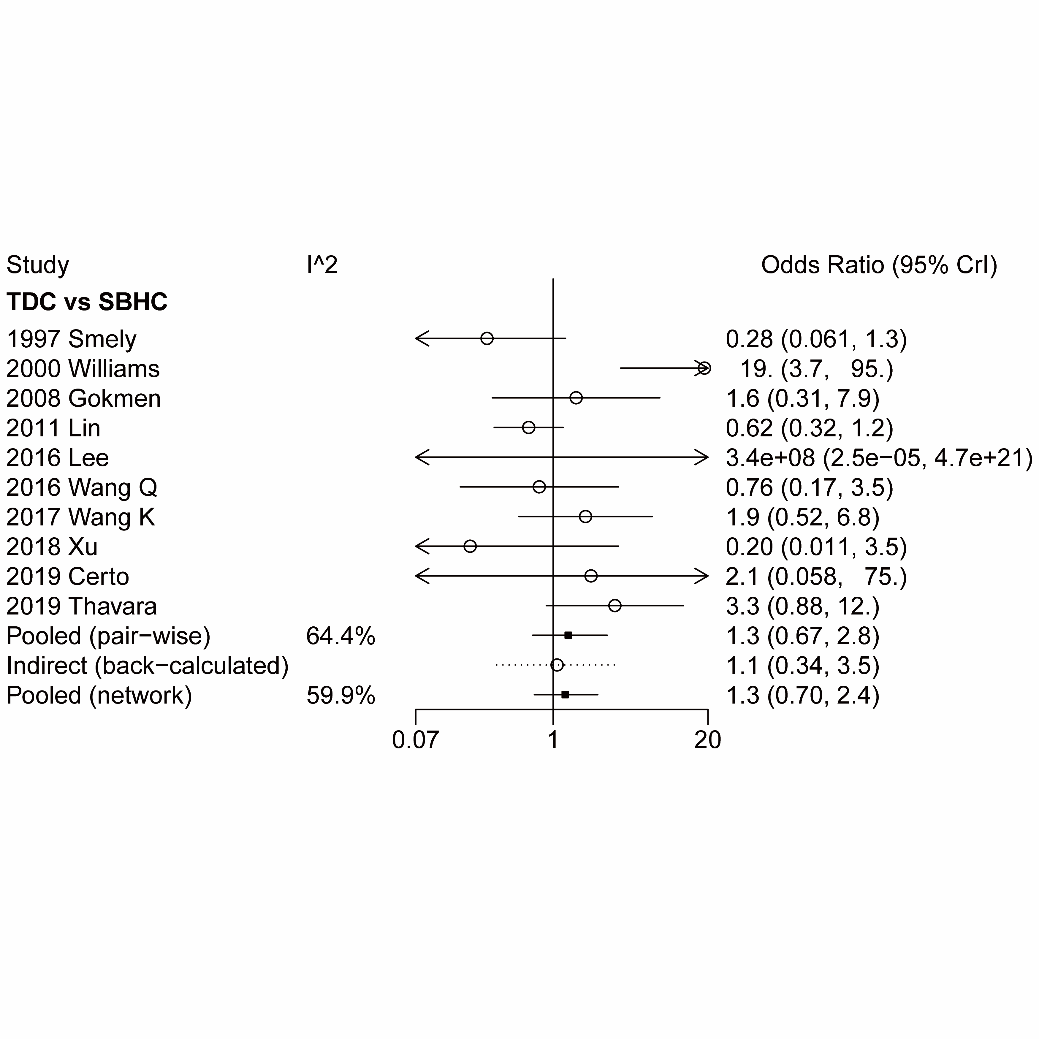


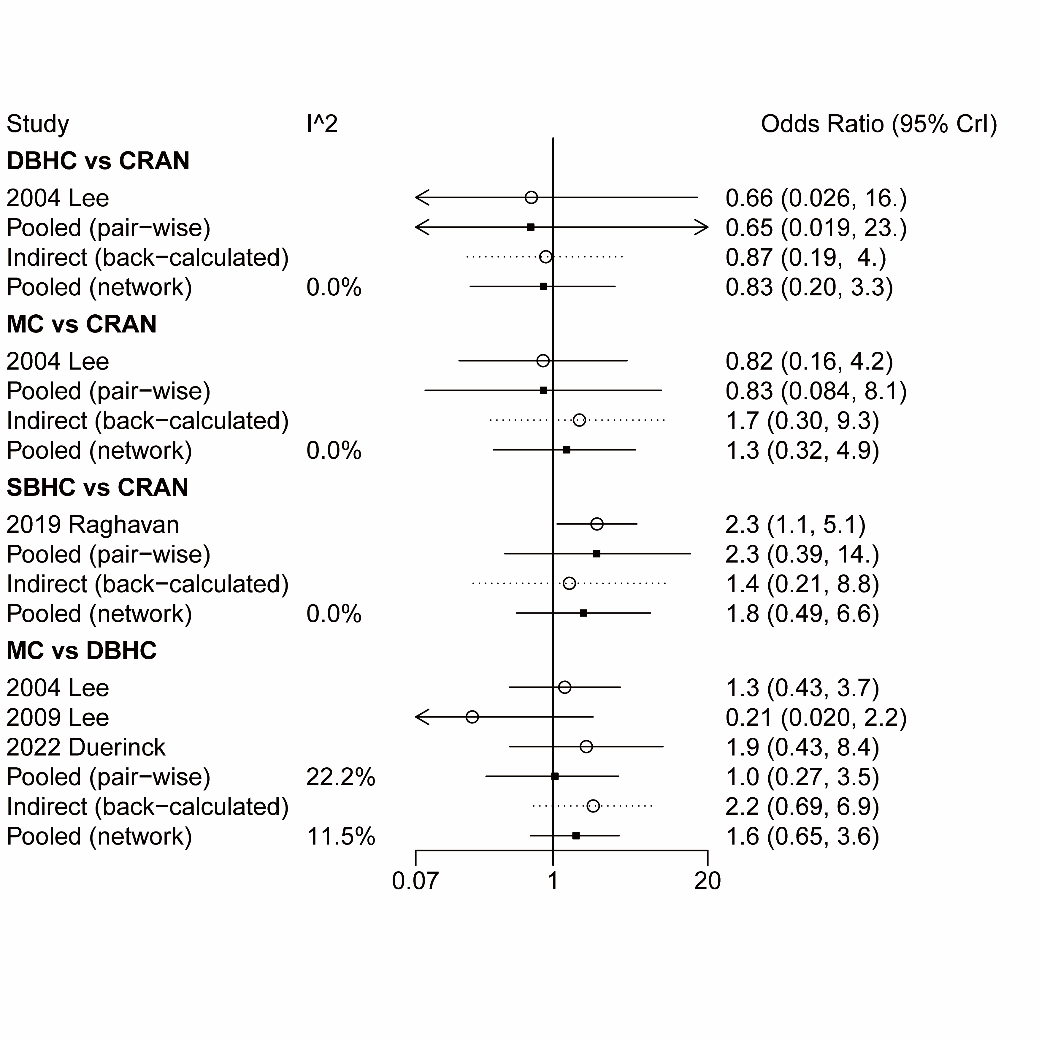


Figure S42: Forest plots for the heterogeneity: Favorable outcome.
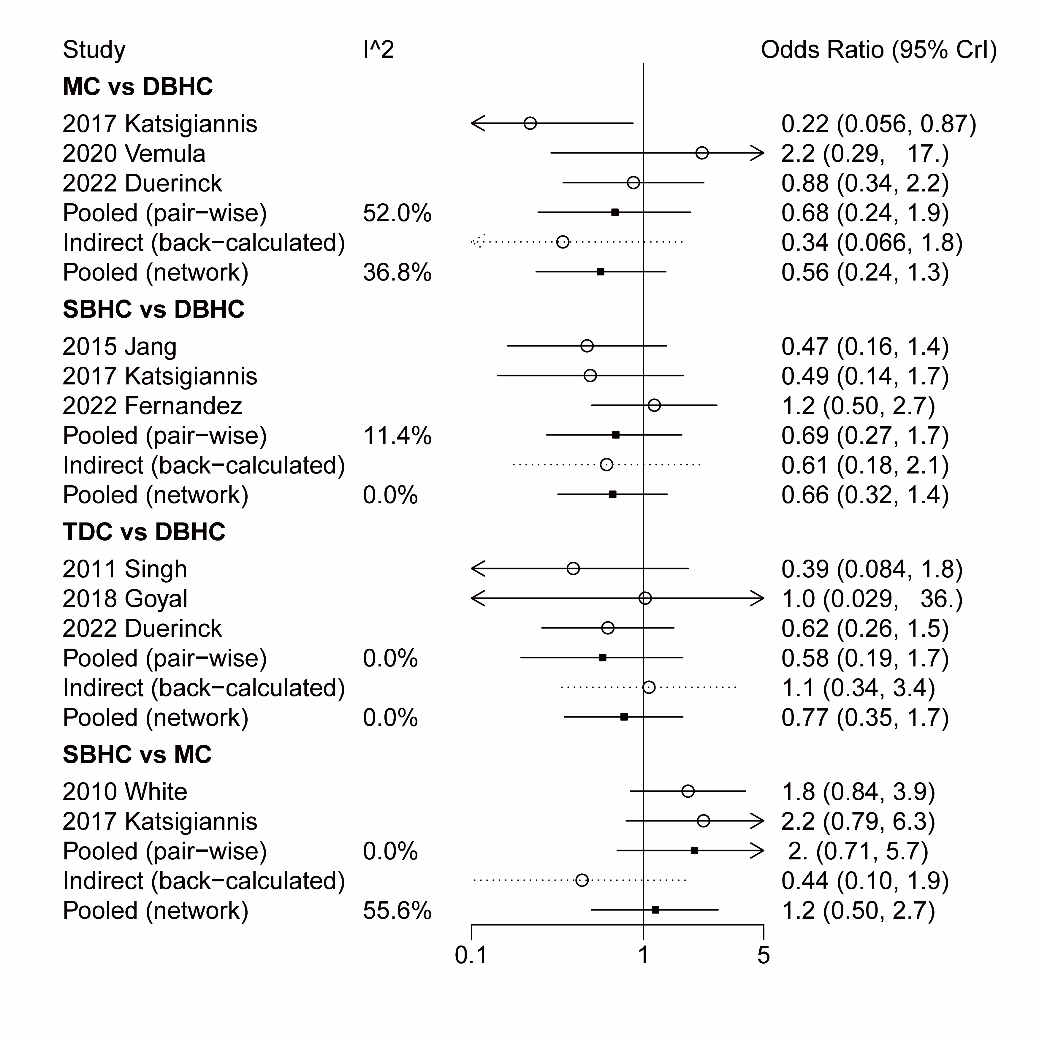

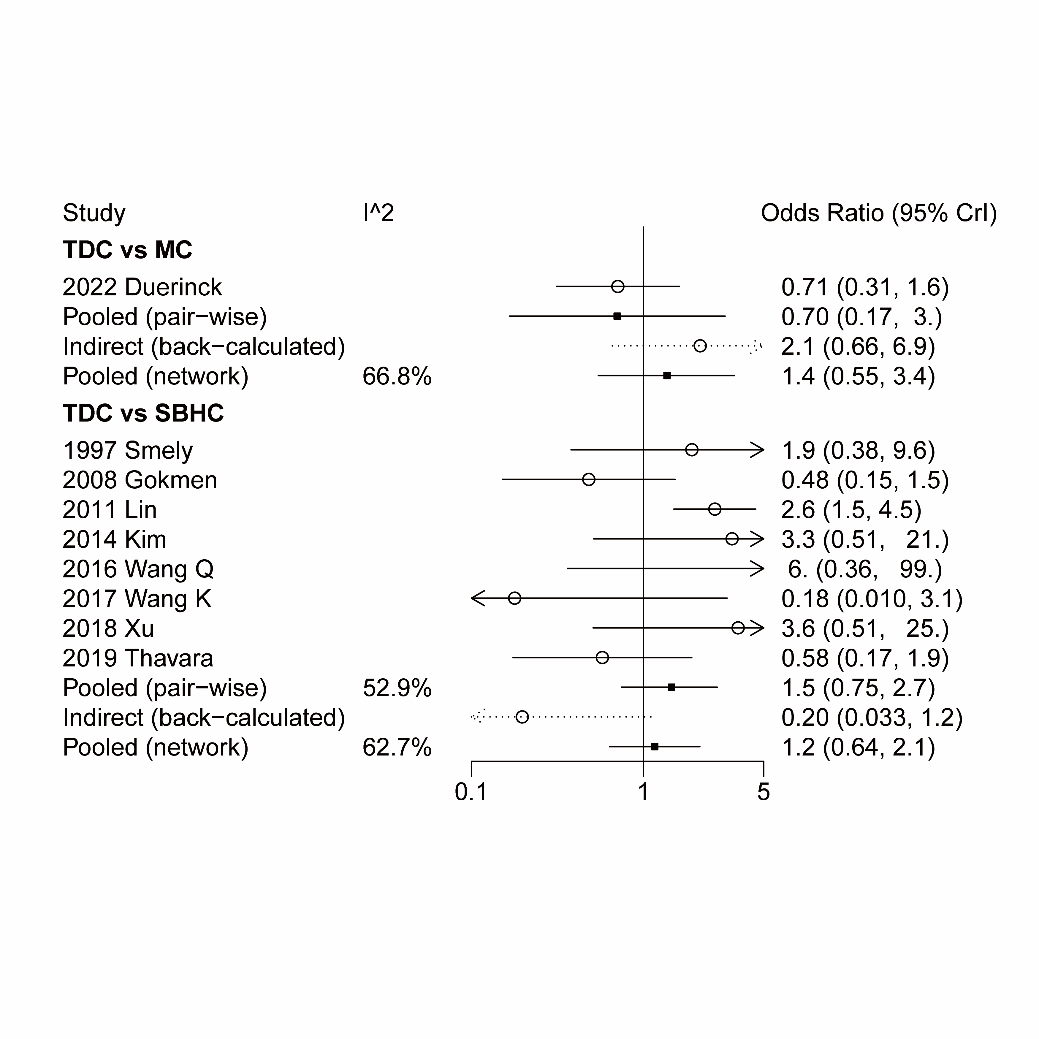


Figure S43: Forest plots for the heterogeneity: LOS.


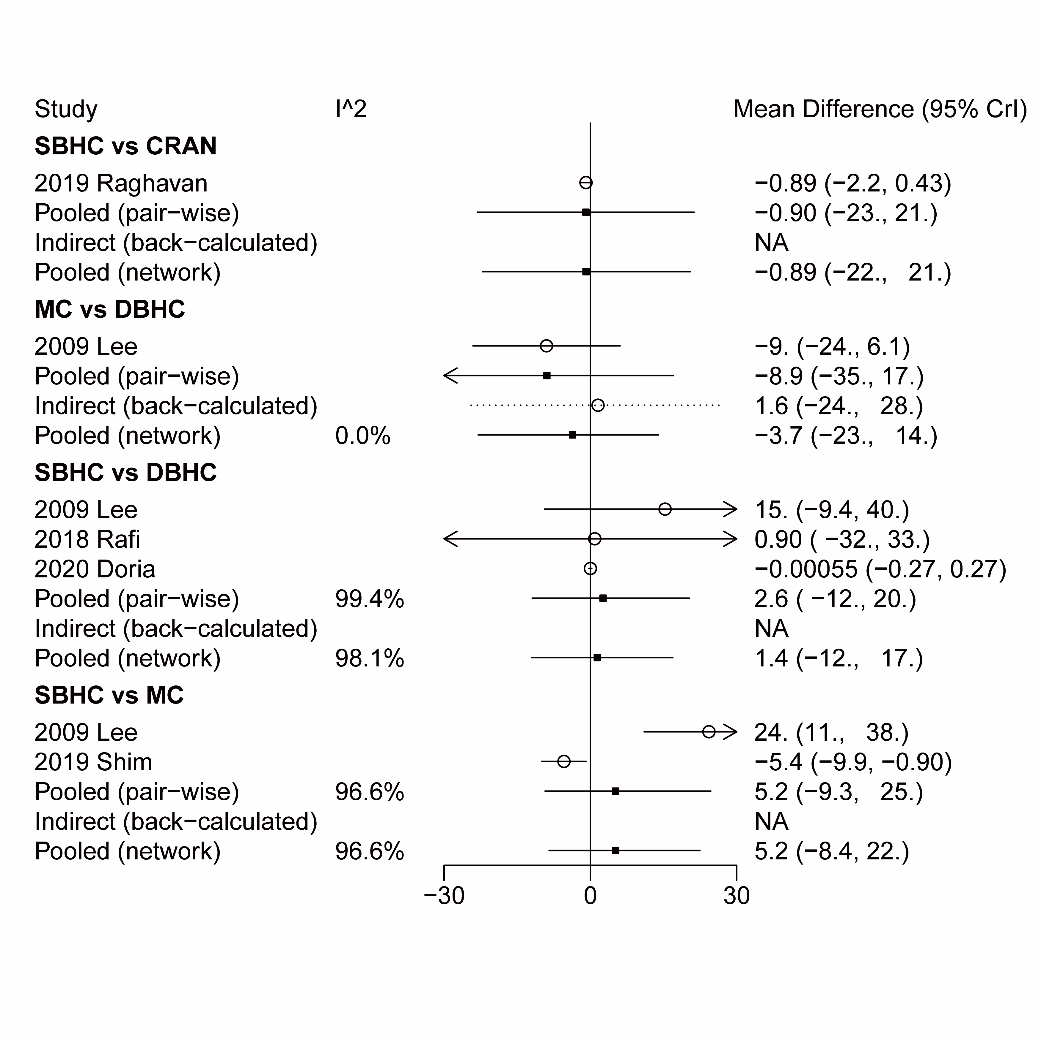


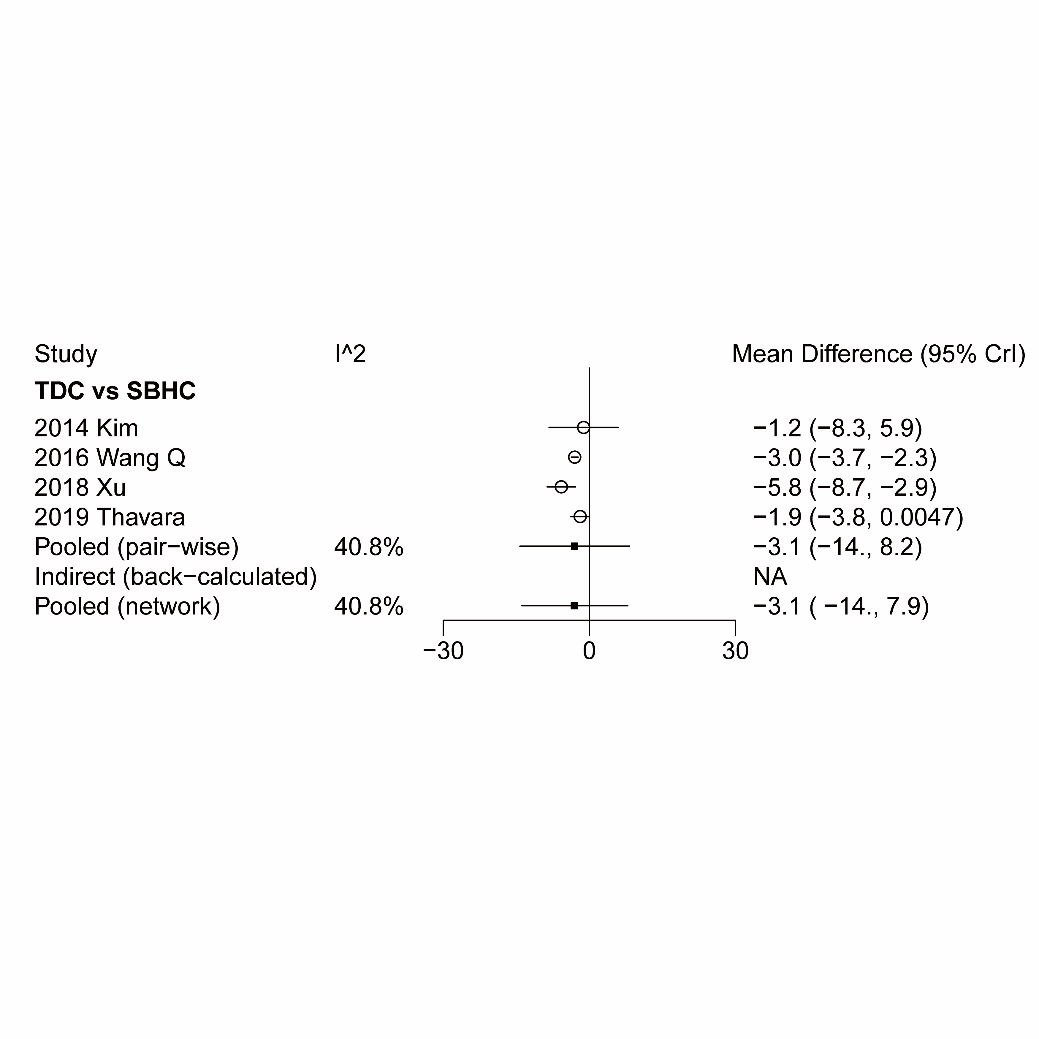


Figure S44: Forest plots for the heterogeneity: Operation time.


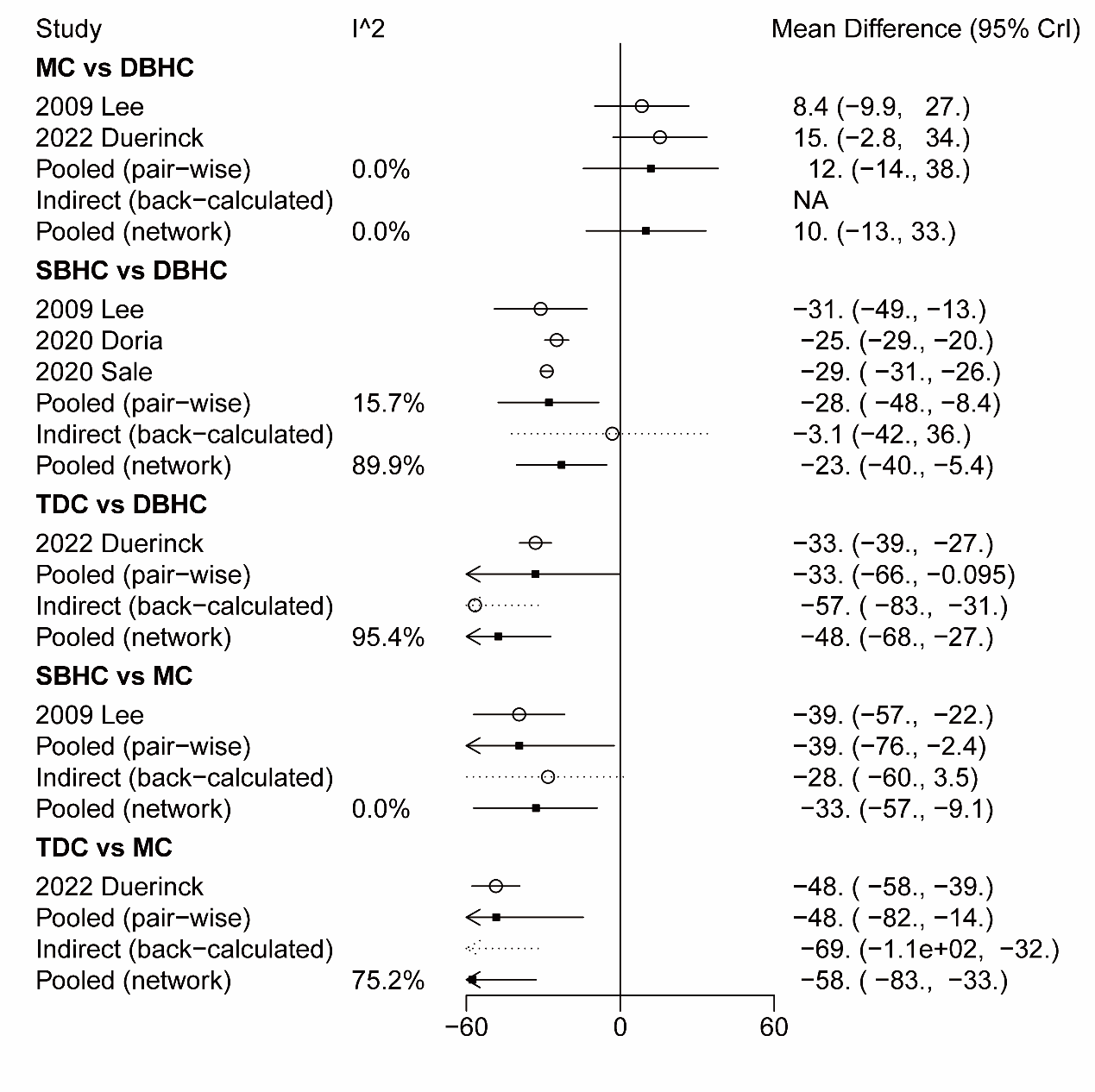

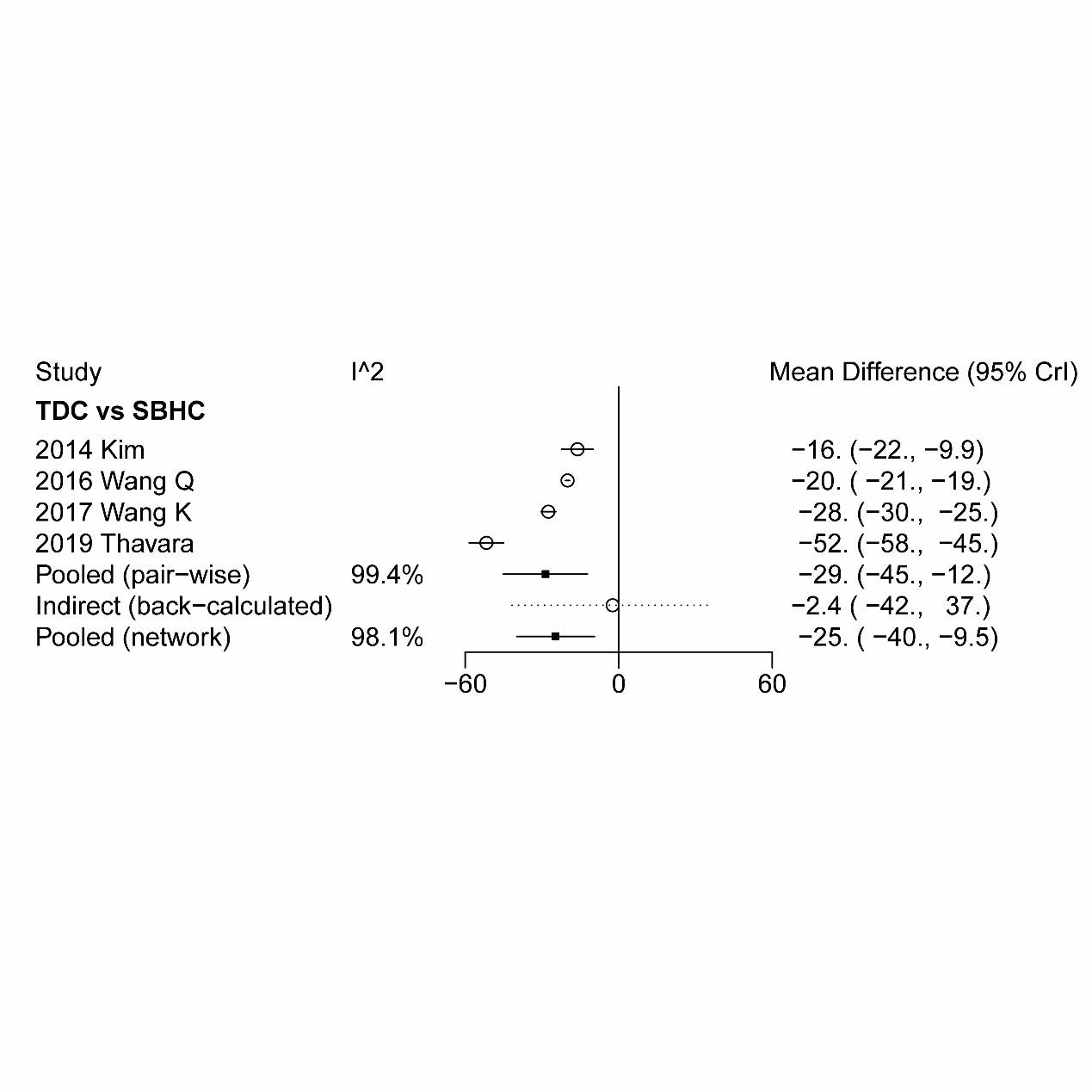


Figure S45: Forest plots for the heterogeneity: Complication.


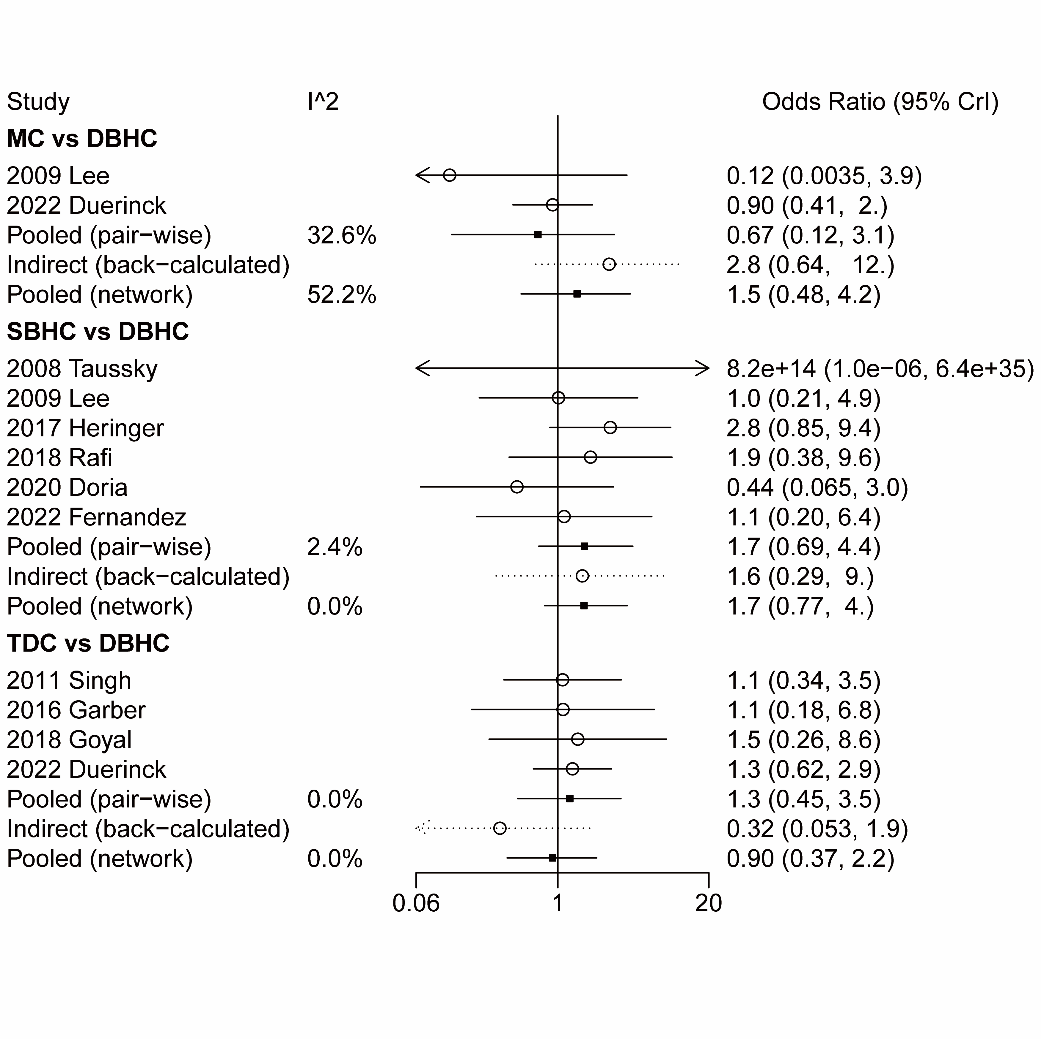


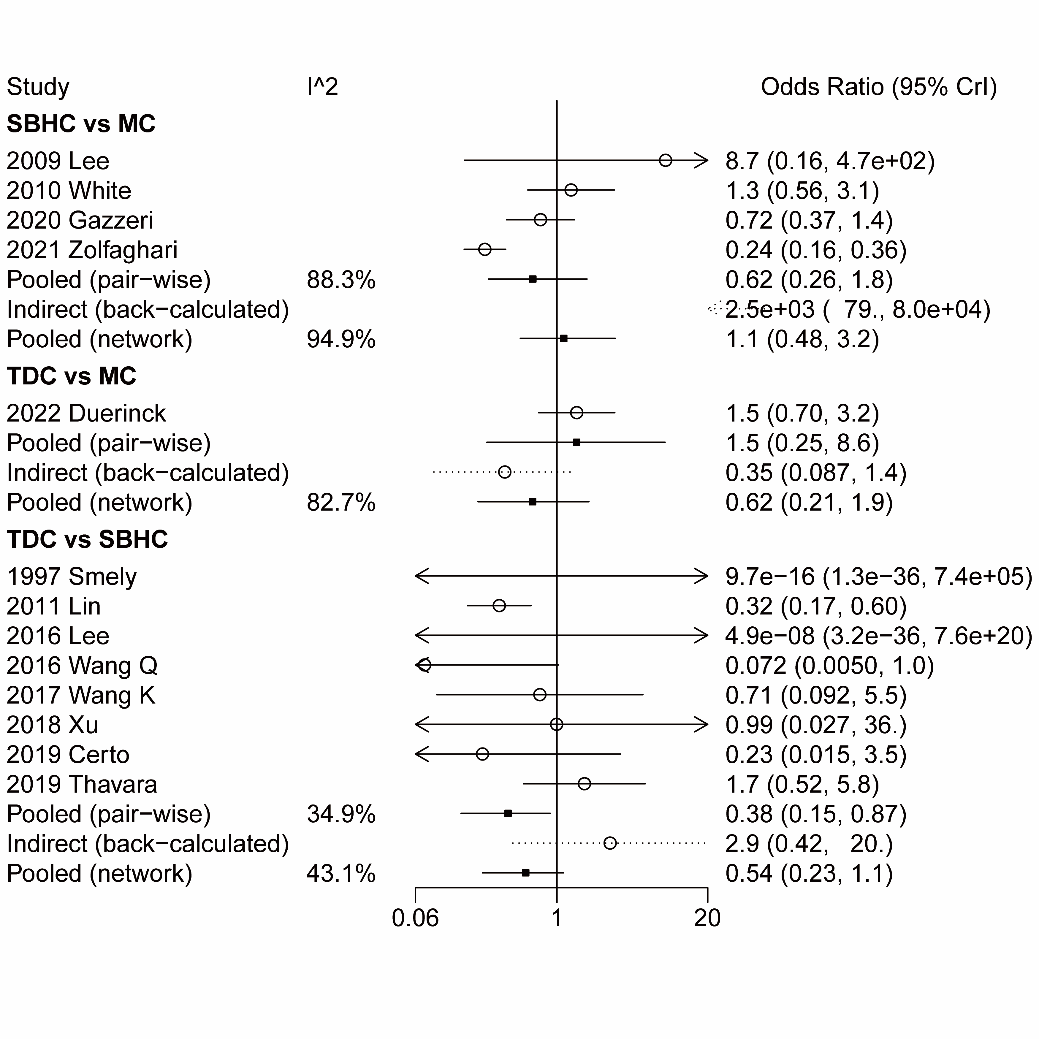


Figure S46: Forest plots for the heterogeneity: Mortality.


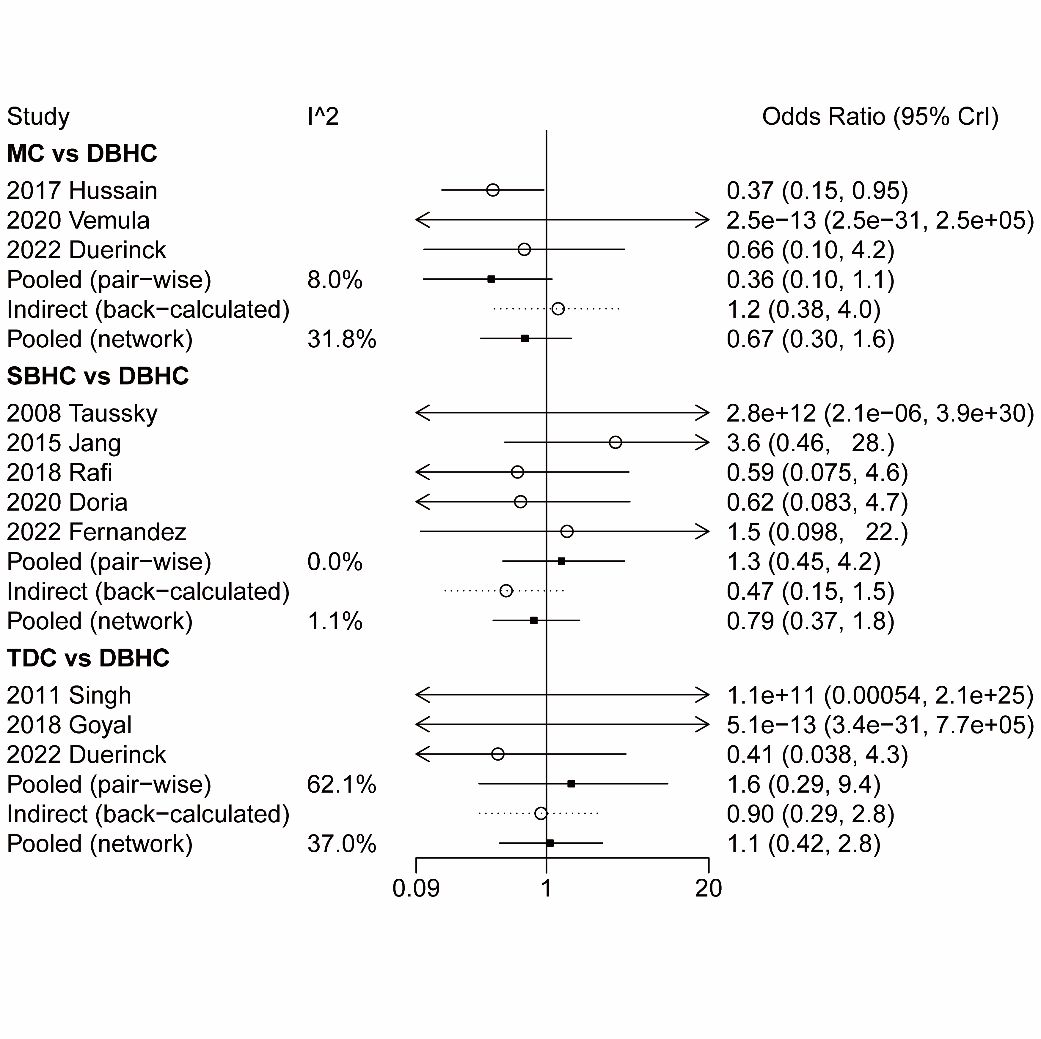

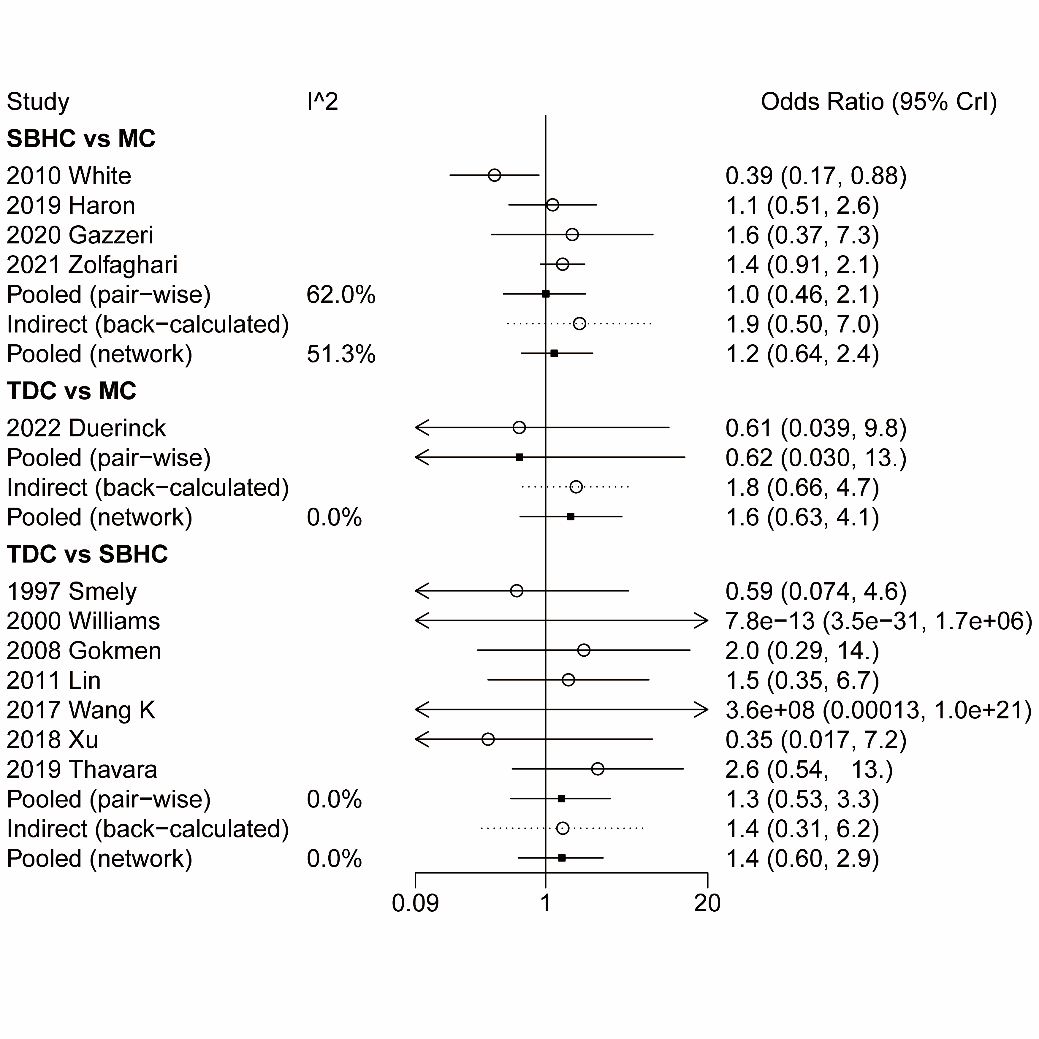


Figure S47: Network meta-analysis results of different Surgical Techniques with drainage for Chronic Subdural Hematoma treatments.


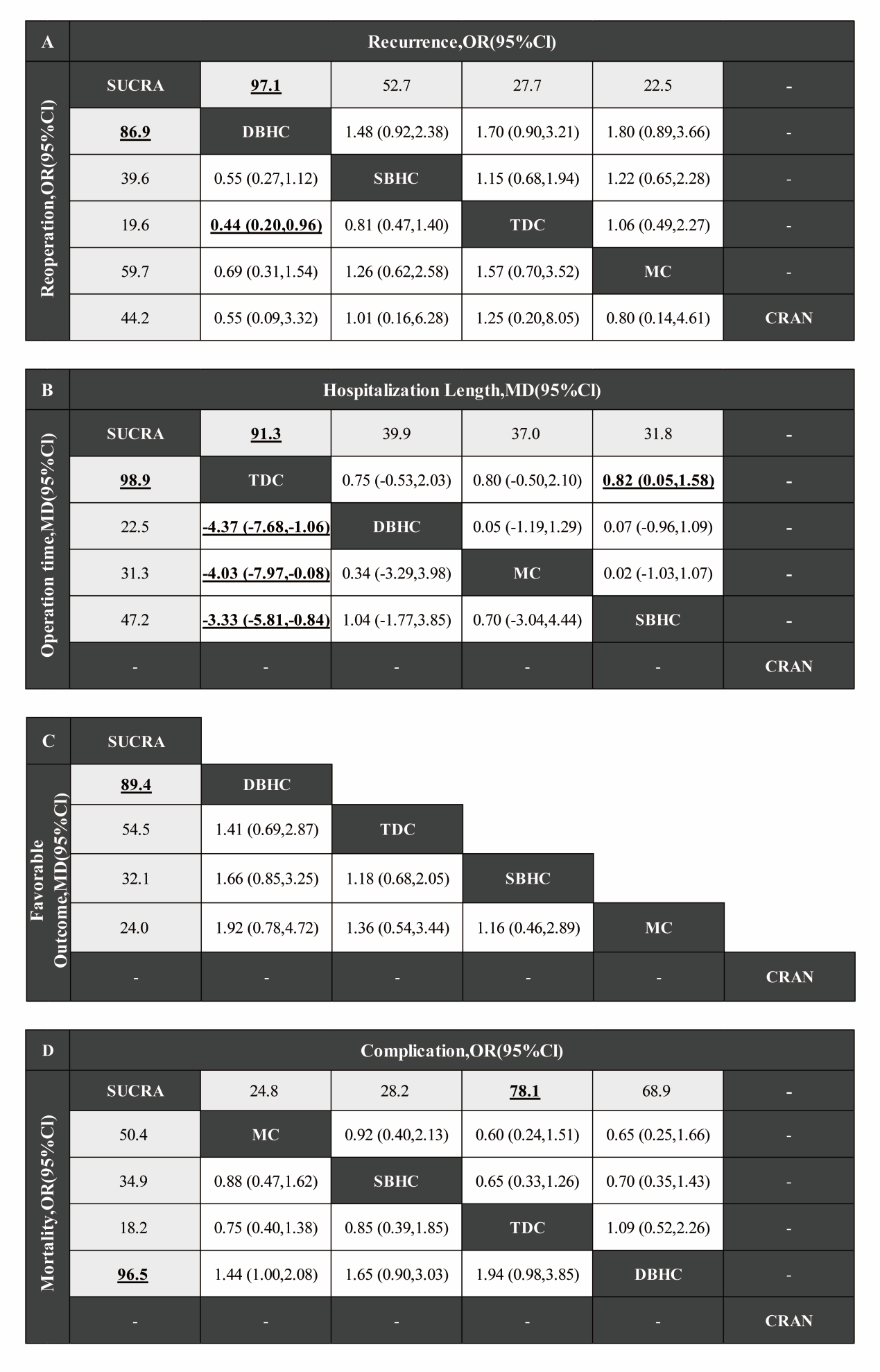


Figure S48: Result of PRISMA checklist.


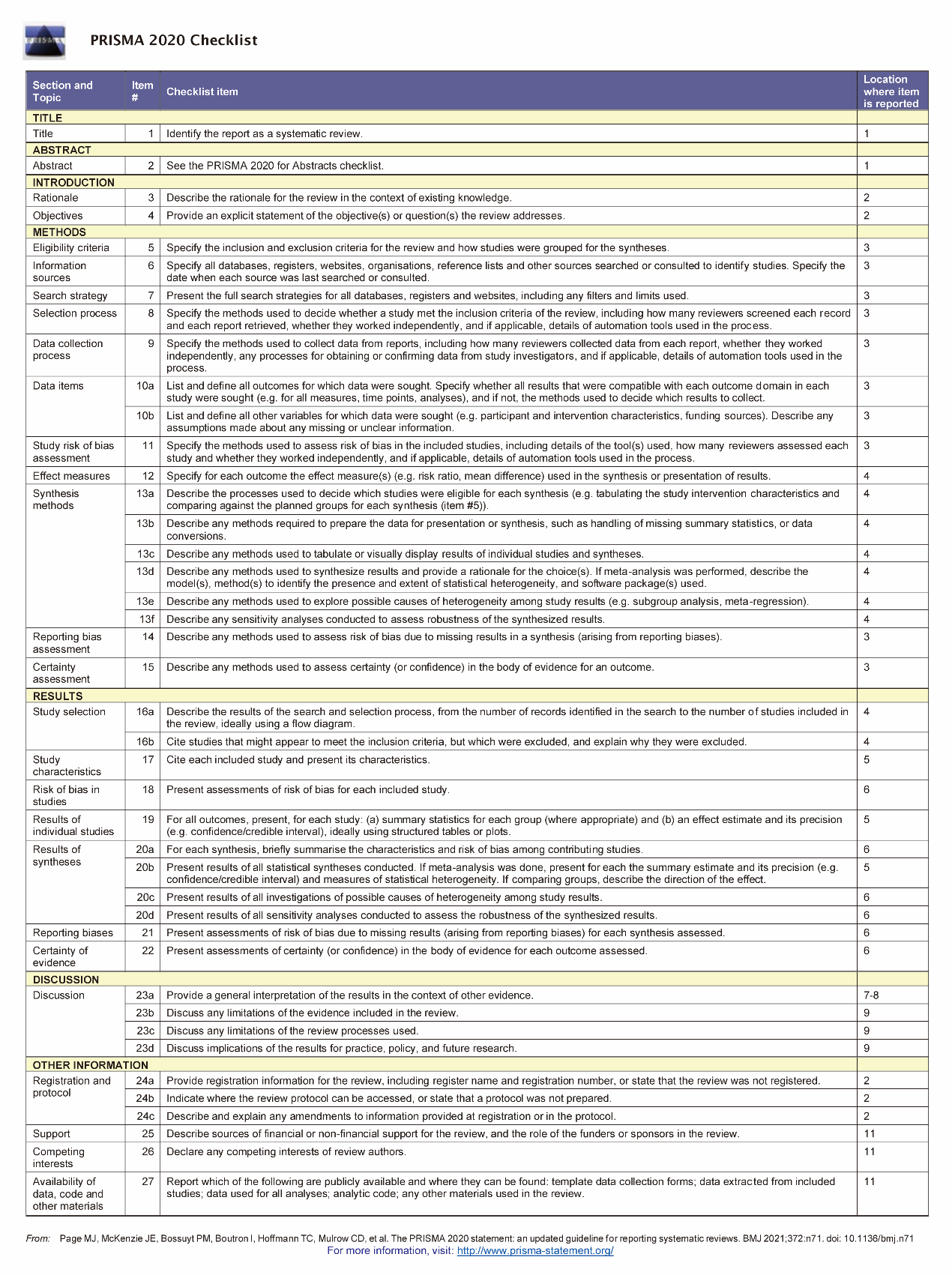

Supplement: Supplementary file 1 [file Data_Sheet_1.ZIP › supplementary material.docx]
